# Clinical importance of high-mannose, fucosylated, and complex N-glycans in breast cancer metastasis

**DOI:** 10.1172/jci.insight.146945

**Published:** 2021-12-22

**Authors:** Klára Ščupáková, Oluwatobi T. Adelaja, Benjamin Balluff, Vinay Ayyappan, Caitlin M. Tressler, Nicole M. Jenkinson, Britt S.R. Claes, Andrew P. Bowman, Ashley M. Cimino-Mathews, Marissa J. White, Pedram Argani, Ron M.A. Heeren, Kristine Glunde

**Affiliations:** 1 Maastricht MultiModal Molecular Imaging Institute (M4I), Maastricht University, Maastricht, The Netherlands.; 2 The Russell H. Morgan Department of Radiology and Radiological Science, Division of Cancer Imaging Research,; 3 Department of Pathology,; 4 The Sidney Kimmel Comprehensive Cancer Center, and; 5 Department of Biological Chemistry, The Johns Hopkins University School of Medicine, Baltimore, Maryland, USA.

**Keywords:** Oncology, Breast cancer, Glycobiology, Molecular pathology

## Abstract

BACKGROUND. Although aberrant glycosylation is recognized as a hallmark of cancer, glycosylation in clinical breast cancer (BC) metastasis has not yet been studied. While preclinical studies show that the glycocalyx coating of cancer cells is involved in adhesion, migration, and metastasis, glycosylation changes from primary tumor (PT) to various metastatic sites remain unknown in patients.

METHODS. We investigated N-glycosylation profiles in 17 metastatic BC patients from our rapid autopsy program. Primary breast tumor, lymph node metastases, multiple systemic metastases, and various normal tissue cores from each patient were arranged on unique single-patient tissue microarrays (TMAs). We performed mass spectrometry imaging (MSI) combined with extensive pathology annotation of these TMAs, and this process enabled spatially differentiated cell-based analysis of N-glycosylation patterns in metastatic BC.

RESULTS. N-glycan abundance increased during metastatic progression independently of BC subtype and treatment regimen, with high-mannose glycans most frequently elevated in BC metastases, followed by fucosylated and complex glycans. Bone metastasis, however, displayed increased core-fucosylation and decreased high-mannose glycans. Consistently, N-glycosylated proteins and N-glycan biosynthesis genes were differentially expressed during metastatic BC progression, with reduced expression of mannose-trimming enzymes and with elevated EpCAM, N-glycan branching, and sialyation enzymes in BC metastases versus PT.

CONCLUSION. We show in patients that N-glycosylation of breast cancer cells undergoing metastasis occurs in a metastatic site–specific manner, supporting the clinical importance of high-mannose, fucosylated, and complex N-glycans as future diagnostic markers and therapeutic targets in metastatic BC.

FUNDING. NIH grants R01CA213428, R01CA213492, R01CA264901, T32CA193145, Dutch Province Limburg “LINK”, European Union ERA-NET TRANSCAN2-643638.

## Graphical abstract

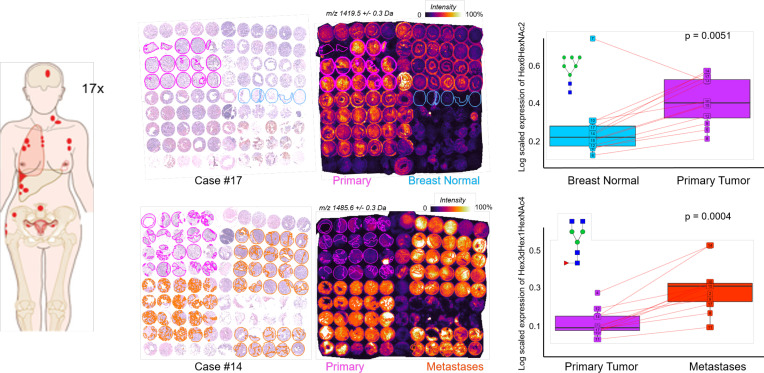

## Introduction

While current therapeutic strategies are successful at treating local primary tumors (PTs), distant metastatic tumors often become resistant to therapy. In fact, the 5-year survival rate for women with metastatic breast cancer (BC) has remained at ~25% for the past 30 years (1). Distant metastasis requires that cancer cells shed from the PT to enter the bloodstream or lymphatic system and attach, invade, and proliferate in distant organs, while evading the immune system (2). The metastatic path is temporally and spatially defined by initially locally confined growth of the PT, followed by spread to nearby sentinel lymph nodes, and finally — in stage IV metastatic BC — disseminated growth of metastatic tumors at distant sites throughout the body. Mounting evidence since the 1980s has demonstrated that this process is not random but, instead, that specific organs are targeted by metastatic BC, which is known as organotropism (3). The development of treatment-refractory metastatic disease has been linked to clonal selection of tumor cells and their altered molecular profiles. Several studies revealed that genetic heterogeneity exists in PT versus subsequent metastases (4), yet to date, the extent of molecular alterations in metastasis remains largely unknown.

Recent studies have demonstrated that glycans — and, in particular, the glycocalyx coating of cancer cells — regulate tumor proliferation, invasion, metastasis, and angiogenesis (5), establishing aberrant glycosylation as a hallmark of cancer (6). Glycosylation is a common form of posttranslational protein modification. It is a complex process in which enzymes such as glycosyltransferases and glycosidases attach or detach glycans (oligosaccharides or polysaccharides) to proteins or other organic molecules (7). Of the different types of glycosylation, O-linked and N-linked glycans are the 2 most common. N-linked glycans have important roles in protein folding, cell–cell recognition, adhesion, and signaling (8), all of which are important in cancer metastasis. Therefore, investigating N-glycans is of utmost clinical relevance, as they may serve as diagnostic markers or therapeutic targets.

Current widely used methods to detect and assess glycosylation changes require bulk extraction of glycans from tumor tissues for analyses that use liquid chromatography coupled to mass spectrometry (LC-MS) (9). While LC-MS approaches offer great sensitivity and specificity, they disrupt the local tissue architecture and, thus, lack spatial histological context. In the last decade, matrix-assisted laser desorption ionization–MS imaging (MALDI-MSI) has demonstrated its ability to locally detect N-glycans directly from the tissue surface while preserving the histological information of the tissue (10). MALDI-MSI studies have shown that aberrant N-glycan profiles are linked to BC subtype (11) and survival rate (12), displaying tissue-specific levels in tumor, stroma, and necrosis (13). The majority of this work has been performed on PTs. However, distant metastases are known to exhibit different gene and protein expression patterns compared with corresponding PTs, and this led us to hypothesize that N-glycosylation patterns may follow the same principle.

Challenges associated with tissue acquisition, especially repeated biopsies of multiple organs during disease progression, are the main reasons for the lack of studies investigating glycosylation changes in PTs versus subsequent metastatic tumors. In this study, we have analyzed the N-glycosylation profiles in a unique set of 17 single-patient tissue microarrays (TMAs) from a rapid autopsy program at The Johns Hopkins Medical Institutions, which ran from 2005 to 2009. These single-patient TMAs contained primary breast tumor, lymph node metastasis, and multiple systemic metastasis cores, as well as normal tissue cores from various organs as controls, all of which were assembled in 1 TMA from the same patient (14) (clinicopathological characteristics are summarized in Table 1).

Full pathology annotation of the entire patient cohort enabled spatially differentiated cell-based analysis. We performed MALDI-MSI analysis to compare N-glycosylation profiles of cancer cells and cancer-associated stroma as they progressed from the PT to different distant metastatic sites. We found that N-glycosylation increased in 71% of patients from PT to metastatic tumors along their metastatic paths, regardless of cancer subtypes and treatment regimens. IHC data from the same cohort of single-patient TMAs, as well as publicly available GE data, were integrated with our MSI-derived glycan data and further corroborate our findings.

## Results

### Patient cohort and study design.

The cohort of 17 metastatic BC patients in this study was represented by 17 single-patient TMAs with a total of 153 control core biopsies, 148 PT core biopsies, and 1399 metastasis core biopsies derived from, in total, 49 different organs. Detailed clinicopathological descriptions of each case are presented in Table 1.

**Figure 1:**
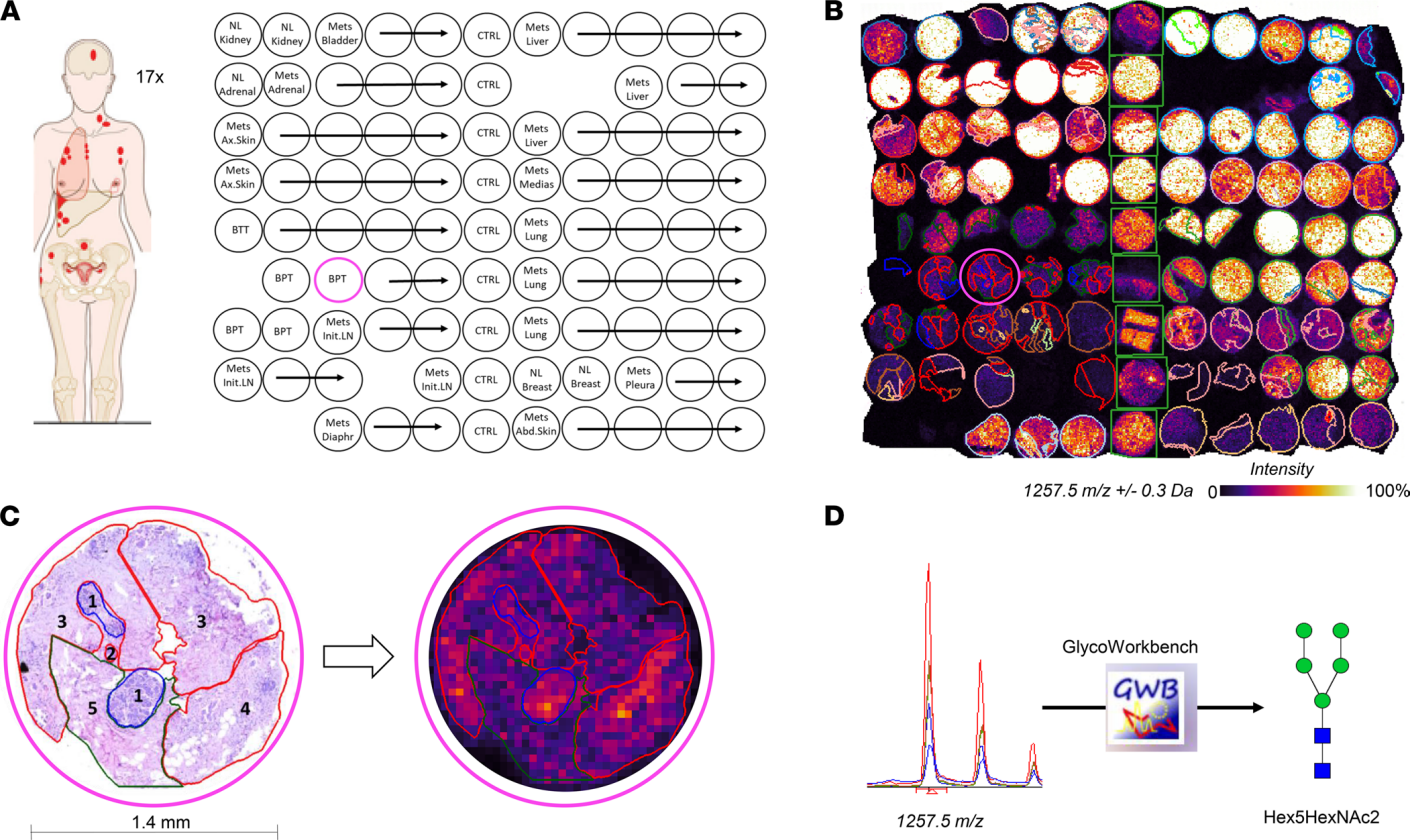
Overview of study design and analysis strategy. (**A**) Schematic layout of TMA #12. Each of the 17 TMAs consisted of 99 cores, with 9 cores of control tissue and 4–5 cores per tumor. BPT, breast primary tumor; BTT, breast treated tumor; CTRL, control; Mets, metastasis; NL, normal; Ax, axilliary; Abd, Abdominal; LN, lymph node; Diaphr, diaphragm; Medias, mediastinum. Pink circle denotes the sample shown in **C**. (**B**) MALDI-MSI data of TMA #12 at a spatial resolution of 50 μm per pixel. The *m/z* 1257.5 map is overlaid with pathological annotations, demonstrating differential abundance between different annotated organ sites. (**C**) Detailed histological annotation of representative breast PT core (left) coregistered with MSI data (right): 1, normal tissue; 2, cancer; 3, cancer mixed with cancer-associated stroma; 4, cancer mixed with lymphoplasmacytic infiltrate; and 5, cancer-associated stroma. Blue lines are for normal, red for cancerous tissue, and green for stroma. (**D**) Identification of *m/z* values of interest from all 17 TMAs was performed using GlycoWorkbench 2.1 software with GlycomeDB database. In this example, *m/z* 1257.56 ± 0.3 Da was identified as Hex5HexNAc2.

To evaluate glycosylation profile differences between healthy breast tissue, PT, and subsequent metastases, we analyzed all 17 TMAs with MALDI-MSI to measure spatially resolved N-glycan abundance. On average, 130,000 pixels (each 50-by-50 μm) were acquired per TMA, resulting in a total of 2,328,548 pixels for the entire data set. A total of 42 unique *m/z* values were observed. MALDI-MSI–measured samples were stained with H&E and annotated in detail by a board-certified pathologist. The main annotation categories were normal, cancer, cancer mixed with cancer-associated stroma, cancer-associated stroma, stroma, and necrosis. In total, 9055 annotations were created for this data set, with 530 annotations on average per TMA. Figure 1, A–C, shows our study design, highlighting the power of MALDI-MSI for delivering spatially resolved glycan abundance, which is complemented by the context of the underlying pathology annotations. The identification strategy is illustrated in Figure 1D.

### Tissue- and metastatic site-specific N-glycans.

We performed statistical testing to identify significant changes (*P* < 0.05) in N-glycans across all tissue type annotations to evaluate the effects of BC progression on N-glycan content of each tissue type and metastatic site. Paired comparisons were performed within each patient/TMA to avoid biological and technical variability between TMAs. To ensure well-powered statistical analysis, we only selected tissue type annotations and metastatic sites that were present in the majority of all TMAs. The frequency of each tissue type annotation and the full resulting list of significantly altered N-glycans are provided in Supplemental Tables 1 and 2 (supplemental material available online with this article; https://doi.org/10.1172/jci.insight.146945DS1), respectively. Section 1 of Supplemental Methods provides detailed descriptions of each pathology annotation category, and Section 2 explains the N-glycan nomenclature.

**Figure 2:**
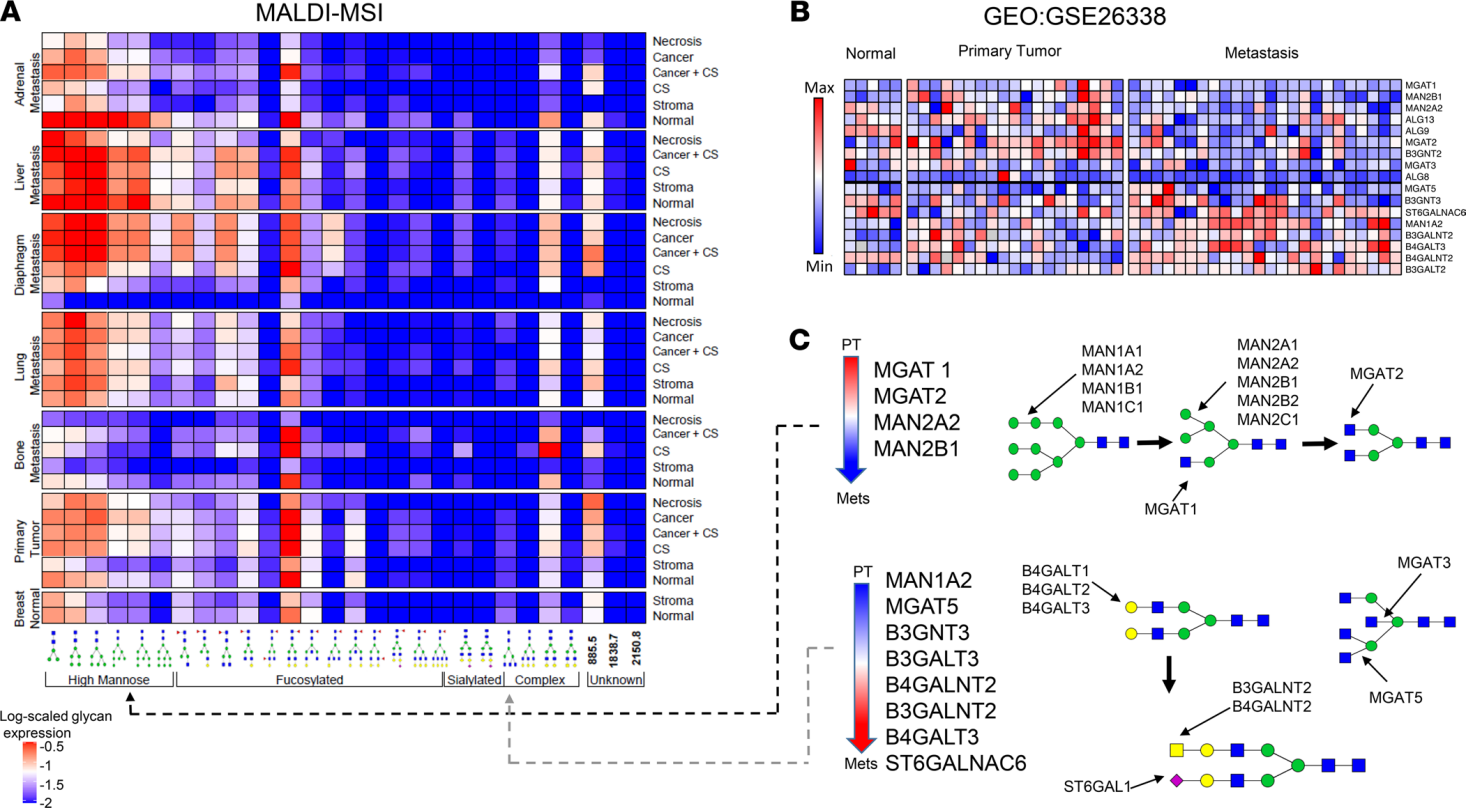
N-glycan abundances grouped by anatomical sites and histological tissue types. (**A**) The heatmap represents an overview of the 28 significantly differentially abundant N-glycans throughout 7 most common organ sites and 6 most common pathology annotations. Each entry represents the average level of a given N-glycan across all 17 TMAs for a specific site and annotation combination. The glycans are grouped based on glycan structure similarities (bottom). MALDI-MSI data are displayed log-scaled. CS, cancer-associated stroma. Bone metastasis consists of pooled bone, spine, vertebra, and rib metastases. (**B**) Data set GEO GSE26338 analyzed for N-glycosylation genes. Heatmap showing significantly altered genes between PT and metastatic tumors (*P* < 0.05, FDR < 0.15). (**C**) Differentially expressed genes shown schematically within the N-glycan biosynthesis pathway. Dashed lines indicate consistent observations in GE and MSI analyses.

We discovered a total of 28 *m/*z values that were significantly altered between metastatic organ sites and tissue type annotations, of which 25 could be assigned to distinct N-glycan identities. The average abundances of these significantly altered N-glycans in the most represented organ sites (normal breast, PT, bone [pooled from bone, rib, spine, and vertebra], lung, diaphragm, liver, and adrenal metastases]) and tissue type annotations (normal, stroma, cancer-associated stroma, cancer mixed with cancer-associated stroma, cancer, and necrosis) are shown in Figure 2A. Our data confirm distinct N-glycosylation patterns in different tissue regions — i.e., normal, cancer, and necrosis as previously reported by Scott et al. (13) — and demonstrate for the first time to our knowledge N-glycosylation specificity in various metastatic sites. Additionally, the cell-specific details of the annotations allowed us to explore glycosylation differences beyond the 3 aforementioned tissue regions. In this study, we observed differences in N-glycan abundance between individual morphologies, including stroma and cancer-associated stroma in PT, diaphragm, lung, and bone metastasis, where cancer-associated stroma showed an N-glycan profile similar to that of cancerous tissue (elevated Hex3dHex1HexNAc4 and Hex5HexNAc4).

Furthermore, we grouped significantly altered N-glycans based on structural features, reflecting the enzymatic steps of N-glycan biosynthesis (8). The resulting groups were high-mannose, complex fucosylated, complex sialylated, and tri-/tetra-antennary complex branched N-glycans. Our data clearly reveal that high-mannose N-glycans were more frequently elevated in metastases compared with PTs and normal breast than the other types of glycans (Figure 2A), with the exception of bone metastasis, which displayed higher levels of fucosylated N-glycans. Several complex branched N-glycans were differentially abundant in specific tissue morphologies. For example, Hex3dHex1HexNAc4 and Hex5HexNAc4 were highly abundant in cancer and cancer-associated stroma but low in physiologically normal stroma (Figure 2A).

To further examine these structural N-glycan differences between PT and distant metastases, we analyzed publicly available gene expression (GE) microarray data (Gene Expression Omnibus [GEO] GSE26338) consisting of > 1000 human breast tumor, metastasis, and normal tissue samples (15). We focused our analysis on genes involved in the N-glycosylation biosynthesis pathway (8). A heatmap was drawn to display genes in GSE26338 whose expression differed significantly between PT and metastases (*P* < 0.05, FDR < 0.15) (Figure 2B), revealing that *MGAT1*, *MGAT2*, *MAN2A2*, and *MAN2B1* were significantly decreased in distant metastases compared with PTs. The enzymes encoded by these genes are involved in the early phase of N-glycan processing with the purpose of trimming down high-mannose N-glycans (Figure 2C), committing them to transformation into hybrid type N-glycans (16). Low expression of these genes would result in high-mannose N-glycan accumulation in distant metastases, which is what we observed with our MALDI-MSI data. Furthermore, *MGAT5*, *B3GNT3*, *B3GALT3*, *B4GALNT2*, *B3GALNT2*, *B4GALT3*, and *ST6GALNAC6* were upregulated in metastasis as compared with PT (Figure 2B). These genes are involved in late complex N-glycans branching and sialylation (Figure 2C) (16), and their products were more abundant in distant metastases, as detected by MALDI-MSI.

### N-glycans increase along the metastatic path.

**Figure 3:**
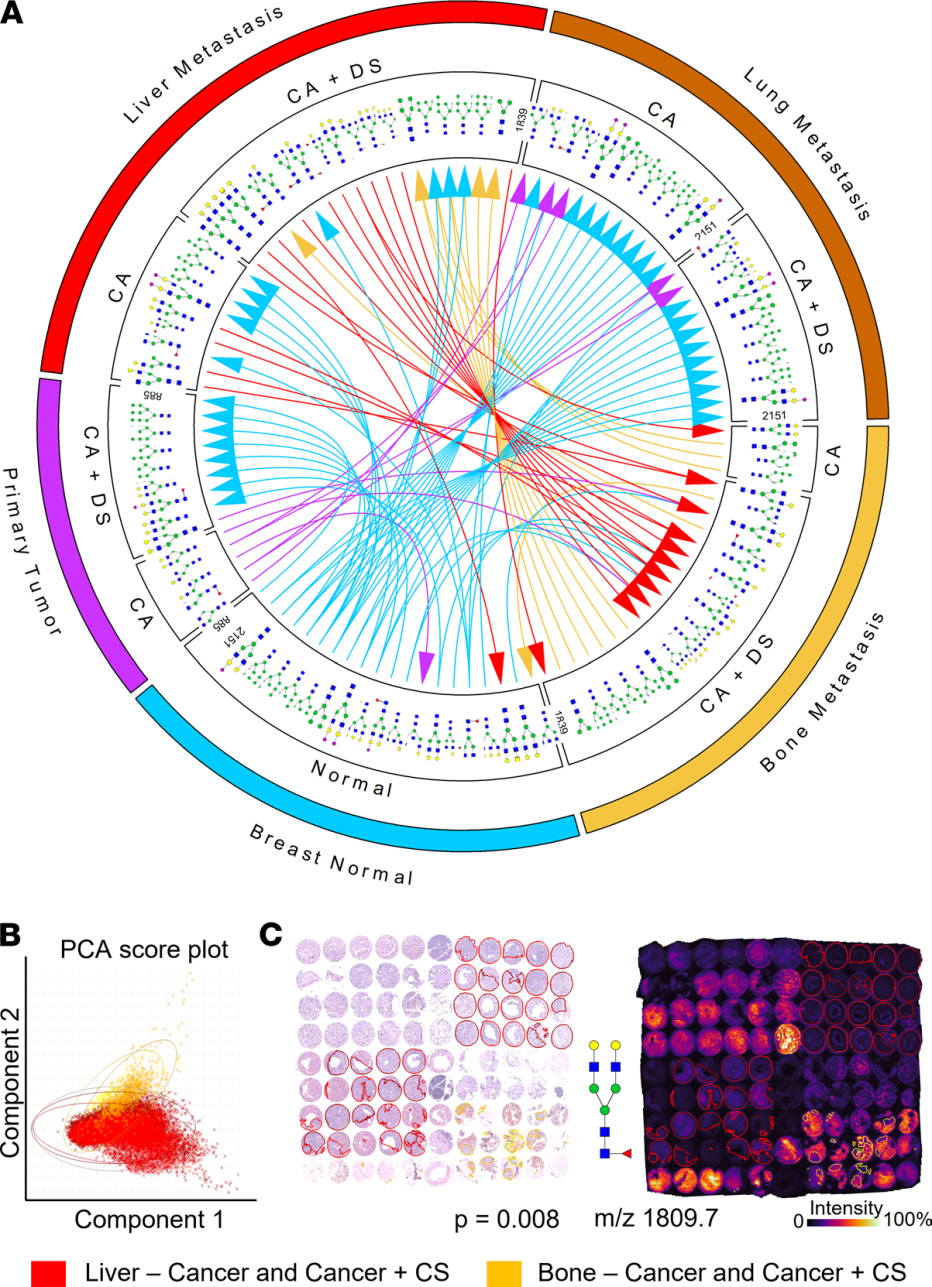
Summary of significantly differentially abundant N-glycans in distant metastatic sites and tissue-type annotations with a focus on bone and liver metastases. (**A**) Comparisons of N-glycan abundance between normal breast and PT, and between the metastatic sites of bone, lung, and liver. The arrows indicate increases in abundance in the direction of the arrow, color-coded in the color of the tissue with lower N-glycan levels. CA, cancer; CA+CS, cancer with cancer-associated stroma. Bone metastasis consists of pooled bone, spine, vertebra, and rib metastases. (**B**) Principal component analysis of TMA #17 showing N-glycan bone (yellow) and liver (red) metastases differences. Score plot for all individual pixels belonging to annotations of CA or CA+CS. PC-1 and -2 show differentiation in N-glycan profile of these 2 metastatic sites. The oval lines indicate 95% CI. (**C**) (Left) H&E staining of TMA #17 metastases with annotations of CA or CA+CS in bone and liver metastases. (Right) MALDI-MSI visualization of one of the most significantly altered N-glycans Hex5dHex1HexNAc4, *m/z* 1809.7 for bone (yellow) or liver (red) metastases with annotations of CA or CA+CS.

The primary goal of this study was to examine N-glycosylation changes during BC progression and development of metastatic disease. Therefore, we focused our analysis on pathology annotations of cancer and cancer mixed with cancer-associated stroma in lung, liver, and bone metastases (Figure 3A), and we found several N-glycans significantly increased in distant metastases versus PTs. Next, we used mRNA-Seq data for PT and organ-specific metastases from the same patient (GEO GSE110590) (17) to analyze genes involved in N-glycan biosynthesis. Similarly, as with the previous GE analysis, *MAN2A2*, *MAN2B1*, *MGAT1*, and *MGAT2* displayed reduced expression, whereas *B4GALT3* and *ST6GALNAC6* were elevated in metastases as compared with the PT (Supplemental Figure 1).

The majority of N-glycans was consistently increased in lung metastasis compared with the PT (Figure 3A), with the underlying N-glycan biosynthesis genes changing accordingly (Supplemental Figure 1). Consistent with the different N-glycan structure abundance observed in bone metastases (Figure 2A), we found several differentially abundant N-glycans between bone and liver metastases, which were not observed across the other annotated metastatic sites (Figure 3A). To investigate these N-glycan differences in bone versus liver metastases, we performed principal component analysis (PCA) (Figure 3B), which revealed 17 significantly altered N-glycans (Supplemental Table 3) between bone and liver metastases, with the greatest difference from Hex5dHex1HexNAc4 (*P* = 0.008; Figure 3C). Most of the N-glycans with higher abundance in bone as compared with liver were fucosylated (Supplemental Figure 2A). Core-fucosylation is a result of 1 sole enzyme — i.e., α-(1,6)-fucosyltransferase (FUT8) (8). FUT8 expression levels examined in GEO GSE110590 (Supplemental Figure 2B) showed higher median value in bone compared with liver metastases. To assess cell type–specific native expression of FUT8 in the bone microenvironment, the Human Cell Atlas (18) bone marrow tissue project was analyzed together with a single-cell RNA-Seq data set (GSE144568) (19). These GE analyses showed that platelets, stromal cells, and megakaryocytes (MKs) express FUT8 at higher levels than any other cell type present in bone tissue (Supplemental Figure 2, C and D). We also observed high levels of BM elements including RBCs (and their precursors) and prominent MKs in regions of native BM immediately adjacent to the cancer in bone metastasis (Supplemental Figure 2E).

**Figure 4:**
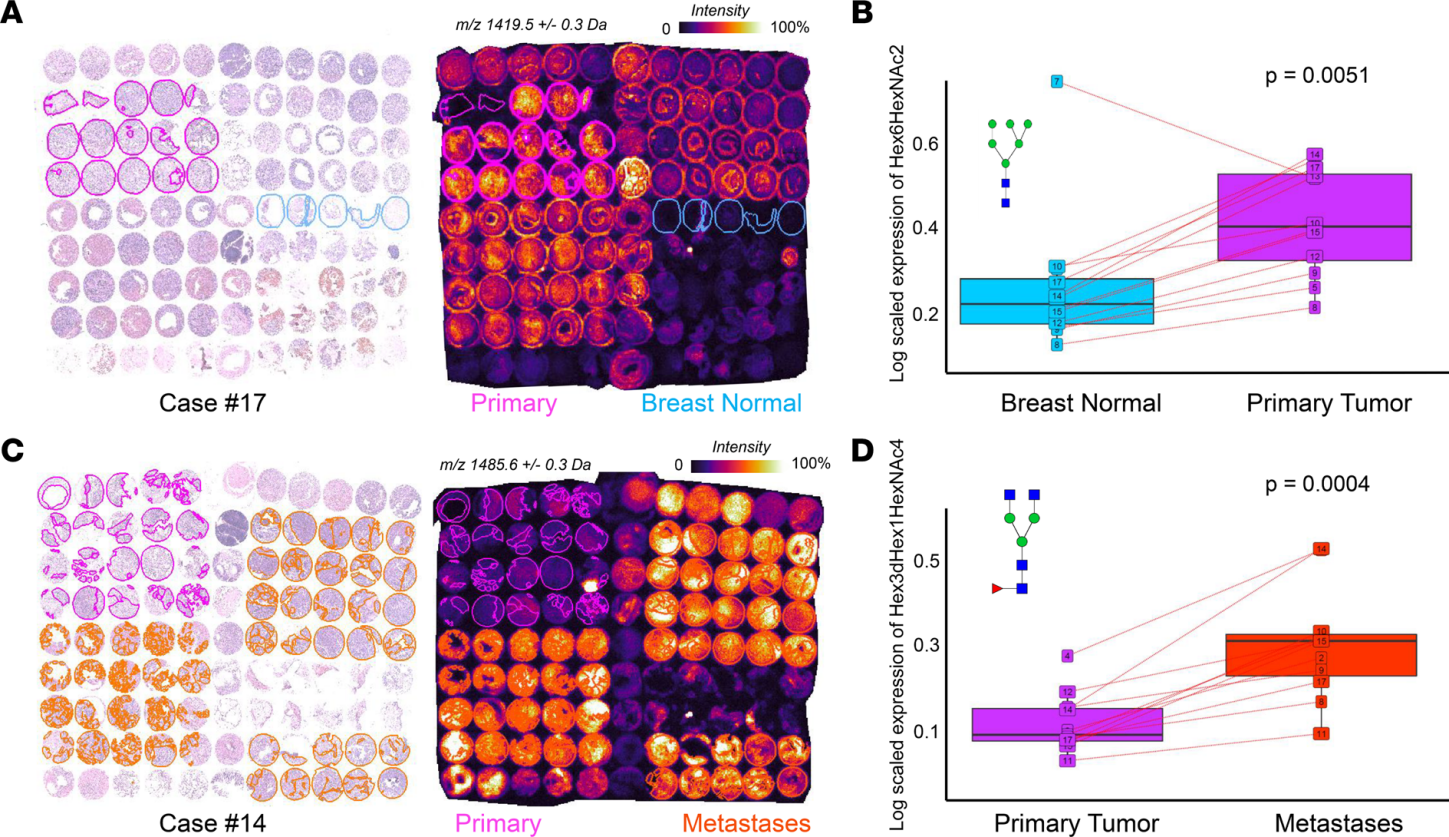
Increased abundance of 2 sample N-glycans from normal breast to PT to metastases. (**A** and **B**) Hex6HexNAc2 was significantly increased in the PT (pink) compared with normal breast (blue) tissue in case #17 (**A**) and across the entire cohort (**B**, *P* = 0.005). (**C** and **D**) Hex3dHex1HexNAc4 was significantly increased compared with PT (pink) metastases (orange) in case #14 (**C**) and across the entire cohort (**D**, *P* = 0.0004). In **A** and **C**, the respective TMA is stained with H&E (left panels) and analyzed by MALDI-MSI (right panels). In **B** and **D**, individual lines show the respective N-glycan expressed in the same patient.

Generally, we observed that the majority of N-glycans increased in PT and metastases compared with normal breast tissue, as illustrated with the visualization of a representative N-glycan in Figure 4A. The statistical evidence for the increase of Hex6HexNAc2 across the entire patient cohort was corroborated in the box plot in Figure 4B. N-glycans significantly increased in distant metastases as compared with PT (Figure 4C), which is evident for the representative N-glycan Hex3dHex1HexNAc4 in the TMA of case #14. This increase was confirmed for the entire patient cohort by statistical testing (*P* = 0.004) (Figure 4D).

**Figure 5:**
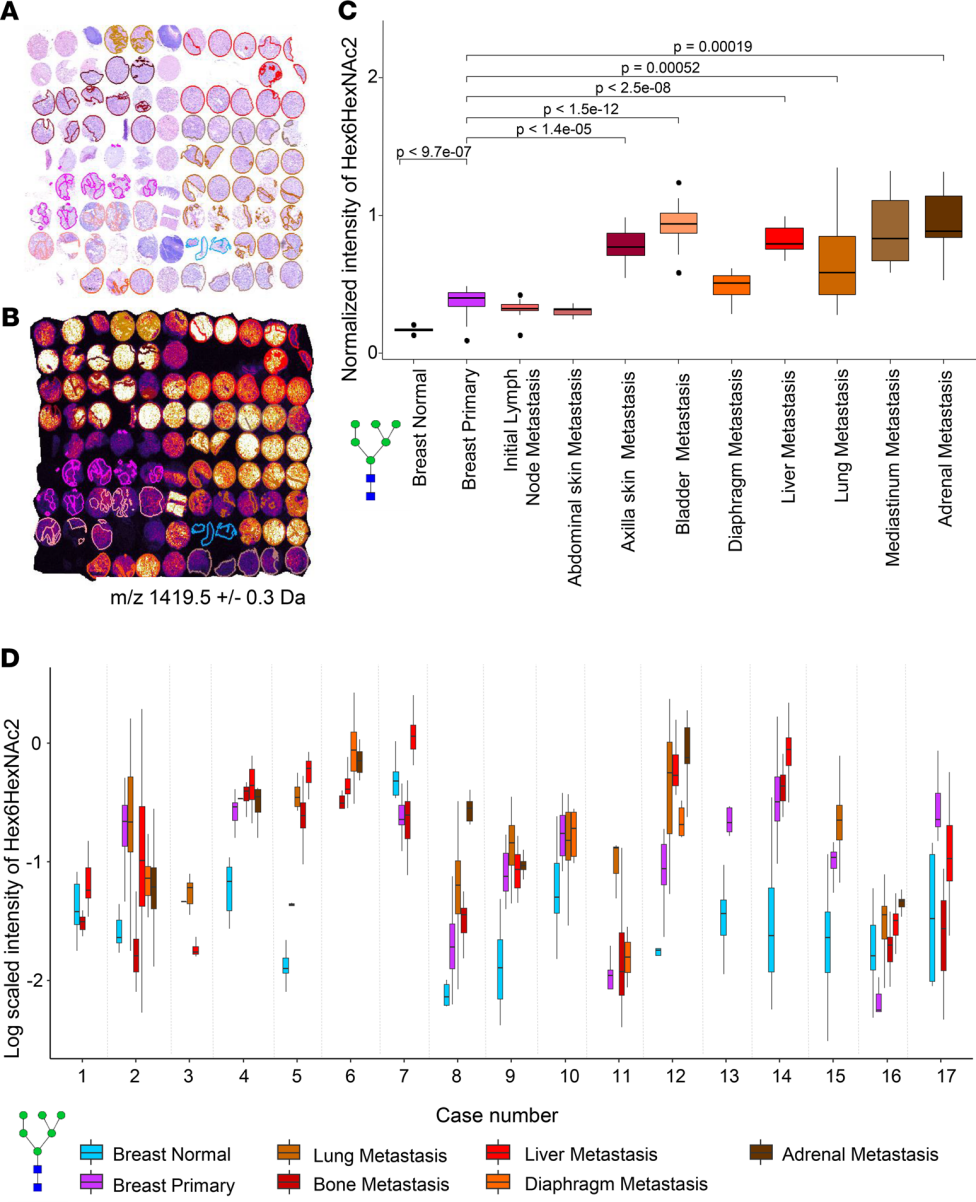
Consistent increase in Hex6HexNAc2 abundance along the metastatic path. (**A**) H&E of TMA #12 showing all annotations of cancer and cancer mixed with cancer-associated stroma for PT and all metastases used for data in **B** and **C**. (**B**) MALDI-MSI intensity distribution of *m/z* 1419.5 (Hex6HexNAc2) in TMA #12. (**C**) Box plots display the average intensity of *m/z* 1419.5 (Hex6HexNAc2) in normal breast, breast PT, and all metastatic sites present in case #12. N-glycan intensities were normalized to normal breast. Bars represent tested pairs and their *P* value after correction for multiple testing. (**D**) Consistent increases in levels of N-glycan Hex6HexNAc2 with cancer progression in 71% of patients. Box plots display the average intensity of *m/z* 1419.5 (Hex6HexNAc2) in normal breast, breast PT, and the 5 most common metastatic sites present in the entire cohort.

Given the significant increase of Hex6HexNAc2, we examined its abundance in other metastatic sites — i.e., initial lymph node, abdominal skin, axilla skin, bladder, diaphragm, mediastinum, and adrenal metastases (Figure 5, A and B). The TMA of case #12 demonstrated a clear, statistically significant increase in Hex6HexNAc2 N-glycan abundance from normal breast to PT to subsequent distant metastatic sites (Figure 5C). In the first metastatic site, the sentinel lymph node, we observed Hex6HexNAc2 levels that were comparable with PT. The intratumor abundance of this N-glycan was quite heterogeneous, as shown by the whisker plots for each tissue (Figure 5C). However, this intratumor heterogeneity of Hex6HexNAc2 was smaller than the overall trend of increasing abundance along the metastatic path. Based on these results, we investigated if the observed trends were consistent throughout the entire cohort of 17 patients. We selected tissue type annotations that were well represented across the data set and plotted the Hex6HexNAc2 abundance for all 17 TMAs across 7 different tissue types (Figure 5D).

Despite the fact that each TMA had its own baseline due to interindividual variation, we found increased Hex6HexNAc2 levels in distant metastatic sites compared with PT and normal breast tissue for the majority of the patients/TMAs. Hex6HexNAc2 at the intermediate stages, from normal to PT and from PT to metastatic sites, generally increased for the cohort with a few exceptions. Two cases (#7 and #16, both of the luminal [Lum] BC subtype) showed a decrease in this particular N-glycan in PT versus normal breast, followed by increased levels in metastases. From PT to metastases, cases #2 (basal-like cancer [BLC]), #3 (Lum/loss), and #17 (Lum/loss) showed lower or equal intensity of Hex6HexNAc2 (Supplemental Methods, Section 3).

Importantly, 12 of 17 patients showed a discernible increase of Hex6HexNAc2 from PT along the metastatic path, which was consistent across patients, BC subtypes (Supplemental Methods, Section 3), and treatment regimens (Supplemental Table 4). The 12 cases that showed this consistent upward trend consisted of 1 HER2+, 1 HER2+/loss, 4 BLC, 4 Lum/loss, and 2 Lum BC subtypes, whereas the 5 inconsistent cases were 1 BLC (case #2), 2 Lum/loss (cases #3 and #17), and 2 Lum BC subtypes (cases #7 and #16). These BC molecular subtypes are explained in Supplemental Methods, Section 3. The majority of these 17 patients underwent mastectomy and/or lumpectomy and were treated with standard radio-, chemo- and/or hormone therapy (e.g., tamoxifen, adriamycin, taxol). Treatment regimens for each patient are listed in Supplemental Table 4.

### N-glycan increase correlates with EpCAM and N-glycosylated proteome in metastatic BC.

Glycosylation of the epithelial cell adhesion molecule (EpCAM) is well documented (20) and has been proposed as promising therapeutic target (21). EpCAM is also involved in a signaling pathway that activates the expression of c-MYC and other oncogenes (22). Therefore, we sought to investigate the link between our N-glycosylation data and EpCAM and c-MYC expression data that were already available from the same patient cohort. For this purpose, we extracted EpCAM IHC data from Cimino et al. (23) and fluorescence in situ hybridization (FISH) data for amplification of c-MYC oncogene from Singhi et al. (24).

**Figure 6:**
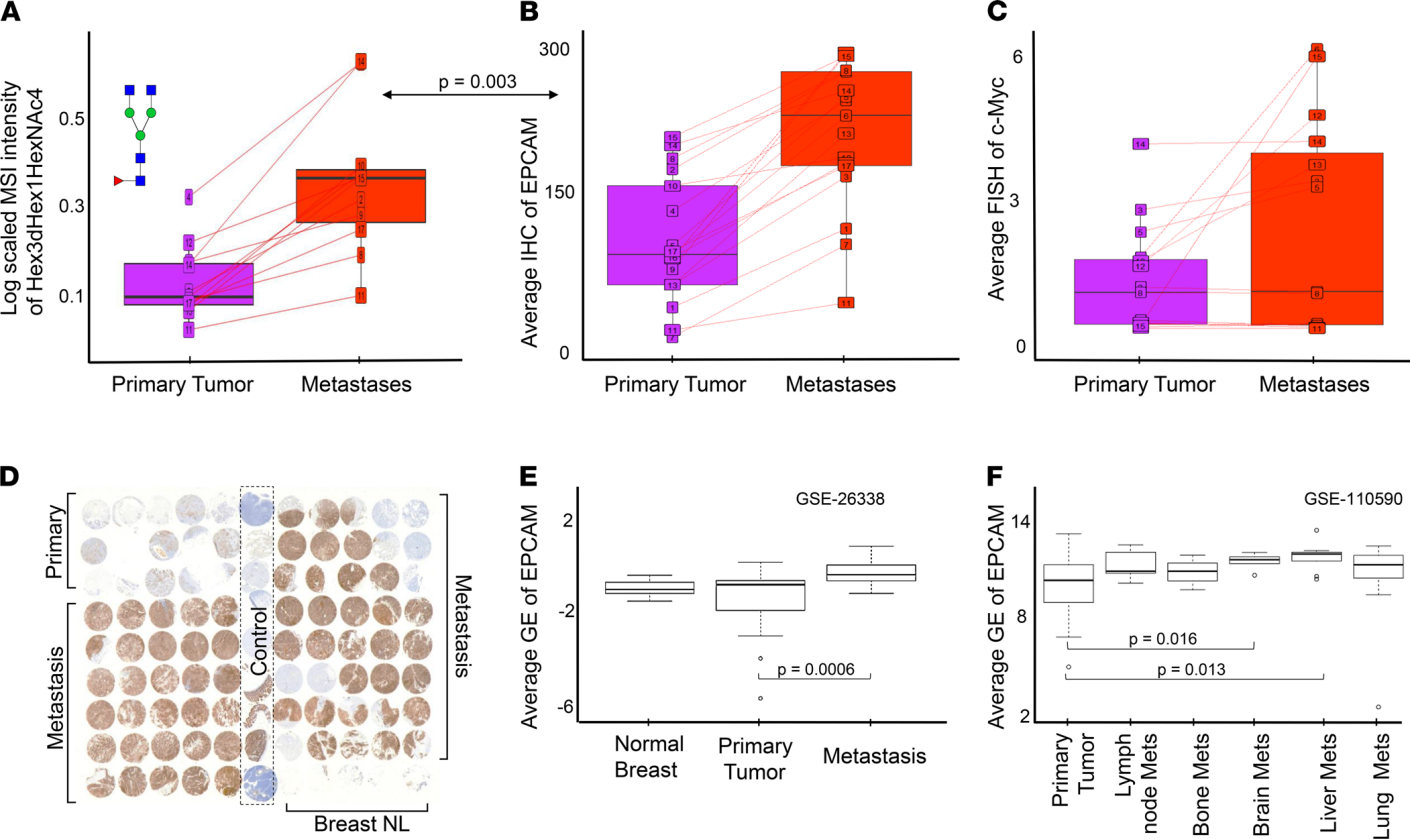
Expression of N-glycans by MSI correlated with EpCAM levels by IHC and GE in primary tumors (pink) and metastases (red) for the patient cohort. (**A**) Abundance of MALDI-MSI of N-glycan Hex3dHex1HexNAc4; the arrow denotes corrected *P* value for correlation of Hex3dHex1HexNAc4 N-glycan levels with EpCAM. (**B**) IHC of EpCAM expression. (**C**) FISH of c-MYC gene amplification. For **A**–**C**, lines connect expression in PT and metastases for individual patients. (**D**) Anti-EpCAM staining of one exemplary TMA #9. (**E**) EpCAM GE expression between normal breast, PT, and metastasis. (**F**) EpCAM GE expression in PT and site-specific metastasis. For **E** and **F**, bars represent tested pairs and their *P* value.

Each of the 42 N-glycan *m/z* was averaged per patient/TMA for annotations of cancer or cancer mixed with cancer-associated stroma for PT and for all metastases pooled together. Similarly, the average expression of EpCAM protein and the average FISH amplification readout for c-MYC were calculated per patient for PT and for all metastases. A significant correlation between EpCAM expression and increase of N-glycan levels in metastases compared with PT was observed in 7 of the 42 *m/z* values tested (Supplemental Table 5). The overall abundance of the most statistically significant N-glycan Hex3dHex1HexNAc4 and EpCAM GE displayed in Figure 6, A and B, showed a correlated upward trend in metastases, followed by slightly elevated FISH c-MYC gene amplification (Figure 6C). Anti-EpCAM staining for a representative TMA (case #9; Figure 6D) showed higher levels of EpCAM expression in metastases versus PT. Using the same GSE data sets as before (15, 17), we observed significantly increased (*P* = 0.0006) EpCAM expression levels in metastasis versus PT (Figure 6, E and F, and Supplemental Figure 3), corroborating the IHC results. In contrast to EpCAM expression, c-MYC amplification did not statistically significantly correlate with the MSI data (Supplemental Table 5).

We set out to further explore the enrichment of BC-related genes encoding proteins within the N-glycosylated proteome. The TCGA BC (25) data set was mined for known genes encoding N-glycosylated proteins from the dbPTM database (https://awi.cuhk.edu.cn/dbPTM/) that are also differentially expressed (FDR < 0.15) in BC (Supplemental Figure 5A and Supplemental Table 6). From these 876 genes encoding N-glycosylated proteins that were differentially expressed in BC, 224 were associated with gene ontology terms related to metastasis (Supplemental Figure 5B and Supplemental Table 7). Next, we analyzed the GSE26338 data set (15) for genes encoding N-glycosylated, metastasis-associated proteins from the dbPTM database (Supplemental Figure 5C and Supplemental Table 8). Supplemental Figure 5, D and E, shows the intercept of differentially expressed genes encoding N-glycosylated, metastasis-associated proteins for both GE data sets. Among these, adhesion molecules including EpCAM and motility proteins including CNTN1 were increased in metastases compared with PT (Supplemental Figure 5E). Supplemental Figure 4 displays full GE data for the reported genes, highlighting the importance of N-glycosylated adhesion proteins similar to EpCAM in BC metastasis.

## Discussion

Aberrant N-glycosylation has been previously reported to occur in BC (11, 12, 26, 27); however, to date, no prior study has assessed N-glycosylation in multiple BC metastases relative to PT from the same patient. Here, we have evaluated 17 single-patient TMAs and demonstrated an N-glycan increase along the metastatic path to be consistent across patients, BC subtypes, and treatment regimens in this patient cohort.

Our data demonstrate that metastatic BC cells have elevated levels of complex branched N-glycans compared with PT (Figure 2). Likewise, we report increased expression of enzymes involved in late complex N-glycan branching (*MGAT5*) and sialylation in distant metastases (Figure 2). GE analysis furthermore showed that 29 N-glycosylated, metastasis-associated proteins, including the adhesion molecules EpCAM, VCAM1, ICAM3, SELE, ITGB2, ITGAL, ITGAX, were differentially expressed in BC metastases versus PT (Supplemental Figure 5). These adhesion molecules confer cell–cell adhesion and cell–extracellular matrix (cell–ECM) adhesion (20, 28). They are frequently glycosylated, which facilitates the affinity binding that makes cells and ECM adhere to each other (29). Dysregulation of N-glycosylation leads to functional disruption of adhesion molecules, which expedites metastasis (5, 29–31). Consistent with our findings, several studies (30, 31) have reported that elevated *MGAT5* expression can increase branched complex N-glycan attachment to cadherins and integrins, which in turn modulate tumor adhesion. This adaptation of BC cells to modify their N-glycosylation profile was not detected in the PT of the same patient in our data. This led us to hypothesize that clonal selection of metastatic cells with complex branched N-glycans resulted in survival of these cells during metastasis and growth at distant sites.

Our study revealed that high-mannose glycans were most frequently elevated in BC metastases, while GE analysis showed lower levels of α-Mannosidase enzymes in distant metastases compared with PT (Figure 2). This is in agreement with a recent finding by Legler et al. (32) that reduced *MAN1A1* expression or mannosidase inhibition in vitro led to significantly increased adhesion of BC cells to endothelial cells. Presence of high-mannose N-glycans on cell adhesion molecules such as ICAM-1 was reported by Scott et al. (33) who concluded that the presence of high-mannose glycans on the ICAM-1 molecule led to increased monocyte rolling and adhesion. In this context, it is reasonable to infer that the high levels of high-mannose N-glycans detected by MALDI-MSI in distant metastatic BC sites served a similar purpose. BC cells likely used their aberrant high-mannose presence to enhance their adhesion to vascular walls, thereby promoting extravasation and subsequent invasion of distant tissues.

The increased expression of EpCAM in PT versus normal breast and metastasis versus PT (Figure 6) is in agreement with our finding of significantly increased N-glycans in distant metastases, as N-glycosylation of the EpCAM protein is well documented (20). Several studies have shown that glycosylation of EpCAM provides stability for this protein (20), whereas deglycosylation leads to EpCAM degradation. Munz et al. (34) even reported hyperglycosylation of EpCAM in malignant tumors compared with normal tissue epithelia of the upper aerodigestive tract. Our MALDI-MSI N-glycan data support these findings. Moreover, we report a consistent increase of N-glycan levels from normal breast to PT to subsequent metastases (Figure 5). While normally EpCAM is a proadhesion molecule, studies have shown that, in cancer, EpCAM can actually loosen the cell–cell interaction (35), thereby conferring advantages for metastasis. Finally, EpCAM is also part of a signaling cascade, during which cleavage of its intracellular domain of EpCAM (EpICD) acts as mitogenic signal transducer as it induces transcription of target genes such as c-MYC, cyclins A and E, and others (20, 22, 34). The FISH c-MYC gene amplification data in our patient cohort also shows an upward trend, following the trends of elevated N-glycans and EpCAM protein in distant metastases. Taken together, our data point toward a potential metastasis-promoting axis, where elevated N-glycosylation, increased EpCAM expression, and c-MYC activation synergize. It is possible that elevated N-glycosylation stabilizes the EpCAM protein, which then stimulates the signaling cascade that results in c-MYC oncogene activation, leading to oncogenic transformation of cells. This is further supported by Cimino et al. (23), who concluded that the overexpression of EpCAM in metastases is likely due to posttranslational regulation such as glycosylation. The obtained MALDI-MSI data do not allow us to directly link the observed increases in N-glycans to the proteins to which they were attached, as on-tissue digest with PNGaseF removed all N-glycans from their respective proteins. Furthermore, the structural identity of the N-glycans attached to EpCAM have not yet been elucidated. Future experiments should investigate the details of EpCAM N-glycosylation and their molecular function in BC metastasis, which is beyond the scope of the present study.

We detected distinctly different N-glycosylation patterns in bone metastases, characterized by relatively lower levels of high-mannose glycans and increased core-fucosylation conferred by increased expression of the FUT8 enzyme. One explanation for this bone metastasis–specific N-glycan profile could be the high abundance of RBCs, platelets, and MKs in core biopsies of bone metastasis. We detected enrichment of core-fucosylated glycans in regions of cancer mixed with cancer-associated stroma in bone metastasis, where high levels of RBCs and MKs were present, and this was not the case in liver metastasis cores. Bone cell–specific expressions analyses (18, 19) confirmed that platelets, stromal cells, and MKs express FUT8 at higher levels than any other cell type present in bone tissue. Taken together, our data support that the highly core-fucosylated N-glycan profile of bone metastasis was likely driven by a local increase of RBCs, MKs, platelets, and stromal cells in bone metastases, all of which highly express FUT8, the enzyme conferring core-fucosylation. Other fucosyltransferases, including FUT1 and FUT2, also play important roles in regulating growth, adhesion, and BC migration (36) and will be explored in future studies.

Our data reveal that several N-glycans increased successively along the metastatic path in each patient, starting with low levels in normal breast epithelium, slightly elevated levels in PT and lymph node metastases, and highly elevated levels in distant metastases. Each primary and metastatic tumor site was represented on average by 5 individual biopsy cores per patient to prevent sampling bias in our data. In addition, all cores in all TMAs were carefully annotated by a board-certified pathologist to enable detailed, cell-based analysis. We were therefore able to assess the intratumor heterogeneity of N-glycan abundance in each patient in our cohort, and it was quite extensive in most patients, as is evident from the large range of the individual box plots (Figure 5). This heterogeneity originates from clonal selection of individual genetically and molecularly distinct cancer cells in response to environmental pressures, resulting in subpopulations of cancer cells able to thrive under unfavorable conditions such as hypoxia or chemotherapy (37). Therefore, intratumor heterogeneity is often associated with poor prognosis and treatment resistance (38). Our patient cohort consisted of 17 terminal metastatic BC patients who had undergone various standard surgical and therapeutic interventions aimed at slowing down disease progression. The aggressive heterogeneous nature of these tumors made them difficult to treat and ultimately led to metastatic disease and death of these patients. The BC subtypes represented in our patient cohort were: 5 luminal types, 5 luminal/loss meaning that the metastatic sites lost their estrogen or progesterone receptor expression, 5 basal-like types, 1 HER2+ and 1 HER2+/loss (Supplemental Methods, Section 3). Our study demonstrates for the first time to our knowledge that N-glycans are robustly elevated in distant metastases over PT over normal breast epithelium, which holds true despite the large intratumor heterogeneity (Figure 5). Furthermore, we show that this N-glycan increase along the metastatic path is consistent across 71% of the patients, despite their BC subtype diversity, different medical and clinical histories, and variable therapeutic interventions. These findings support that N-glycosylation increased in distant metastatic nodules at lethal stages of BC independently of the underlying BC subtypes of PTs. Except for 1 patient (case #12) who had a longer postmortem interval for her autopsy, all other 16 cases experienced a 1- to 4-hour postmortem interval for their autopsy. Since the reported N-glycan findings were consistent within all 17 patients with no outliers, we included all 17 patients in this study. However, it is possible that glycosylation could be altered by the length of the postmortem interval.

Our observation of consistently elevated N-glycosylation of primary breast tumor and distant metastases makes N-glycosylation a potential therapeutic target. Several therapies targeting or inhibiting N-glycosylation have undergone clinical evaluation (39). Our data suggest that it might be worthwhile investigating therapeutics that enhance the activity of the mannosidase I group of enzymes, given the accumulation of high-mannose N-glycans in PT and metastatic breast tumors in our study. Targeting N-glycans could also improve the efficacy of immunotherapy. Recently, Li et al. (40) discovered that N-glycosylation, specifically poly-LacNAc presence catalyzed by B3GNT3, is required for programmed death ligand-1(PD-L1) and receptor programmed cell death protein 1 (PD-1) interactions and suppression of T cell activity. Their findings advocate for targeting protein glycosylation as a potential strategy to enhance immune checkpoint therapy (41). Poly-LacNAcs were found to be elevated in advanced HER2^+^ and BCL metastatic BC tissues in a recent MSI study (11). While our MSI data were acquired in a mass range lower than the mass of poly-LacNAc glycans preventing their detection, our GE analysis revealed higher levels of B3GNT3 and MGAT5 in metastases, demonstrating elevated activity of enzymes needed for poly-LacNAc production. Further investigations are necessary to evaluate the potential benefits of N-glycan modulation in immunotherapy on BC progression. One primary concern for pursuing N-glycans as cancer treatment targets is drug safety and possible side effects, since N-glycosylation is a common form of posttranslational modification in many normal tissues throughout the human body.

For the first time to our knowledge, our data firmly establish upward abundance of N-glycans in tissues along the metastatic path (Figure 5). In agreement with our study, de Leoz et al. (26) have observed that patients with BC have elevated levels of high-mannose N-glycans in their blood serum as compared with healthy subjects, and that these high-mannose N-glycan levels continued to rise with disease progression. Our MALDI-MSI findings reinforce the results from de Leoz et al. (26) as we show increased abundance of high-mannose N-glycans in cancerous tissues in the PT and distant metastatic sites, with an additional increase along the metastatic path (Figures 2 and 5). It is well accepted that circulating tumor cells (CTCs) that have shed from the PT are faced with several stressors such as hemodynamic shear stress and immune attack as they travel in the bloodstream; these stressors are aimed at destroying these CTCs before they can extravasate and establish metastatic growth (42). It is therefore possible that increased high-mannose N-glycan content in the blood sera of BC patients, as detected by de Leoz et al. (26), resulted from CTC cell death in the bloodstream; the cell death would release their cellular glycoproteins into the bloodstream. Serum-based analyses, such as presented by de Leoz et al. (26), extract these glycoproteins from blood serum and treat them with PNGaseF enzyme to release the N-glycans from their respective proteins, yielding the observed high-mannose N-glycan results. To overcome PNGaseF-induced limitations of linking the observed N-glycan signatures to their carrier proteins, Black et al. (43) recently developed an antibody panel–based MS platform for the multiplexed detection of N-glycans in a protein-specific manner from body fluids, including serum. This new platform technology allows for detection of N-glycan signatures with respect to their antibody-detected carrier proteins. As our patient study of metastatic BC clearly shows that elevated high-mannose N-glycans are an important and highly robust biomarker of BC metastasis at the tissue level, it elevates the impact and validity of serum-based diagnostic approaches of high-mannose N-glycans as reported in de Leoz et al. (26) and Black et al. (43). While detection of high-mannose N-glycans in blood serum holds diagnostic potential for early detection of metastatic BC, in particular because it is noninvasive, it has to rely on solid evidence that high-mannose N-glycans are indeed elevated in metastatic BC cells; we clearly demonstrated this in our study. Since our study and de Leoz et al. (26) consistently report an increase in high-mannose N-glycan as BC develops and progresses, novel treatment-responsiveness assays could be designed based on monitoring levels of high-mannose N-glycans in blood serum and/or from tissue biopsies.

Our extensively annotated MALDI-MSI data reveal that cancer-associated stroma exhibits an N-glycan profile that is similar to that of cancerous tissue but not normal physiological stroma. For example, N-glycans Hex3dHex1HexNAc4 and Hex5HexNAc4 exhibited increasing abundance in diaphragm, lung, and bone metastasis starting from low levels in physiological stroma to higher levels in cancer-associated stroma and cancer (Figure 2). Therefore, it would be of interest to further examine the differences between physiologically normal stroma versus cancer-associated stroma at an early stage and late stage of malignancy. The important role of the tumor stroma in cancer progression is clearly emerging, leading to suggestions that targeting the stromal component may improve response to therapy (44). It is well known that normal stroma is essential for the maintenance and integrity of epithelial tissues, containing a multitude of cells that collaborate to sustain normal tissue homeostasis. Genetic and molecular adaptations during carcinogenesis were shown to ultimately change the stromal component of the host tissue, thereby establishing a supportive environment to enable cancer cell growth (45). Furthermore, abnormal glycosylation of cell–ECM adhesion molecules (31, 46) and other glycoproteins that are part of the ECM was found to occur in highly aggressive cancer. Investigating whether the differences in N-glycosylation in cancer-associated stroma versus normal stroma are due to changes in cell types and/or changes in ECM production is necessary. Our 17-patient cohort did not present sufficient numbers of normal and cancer-associated stromal annotations to make statistically supported comparisons. Future studies should explore these important stromal N-glycan changes to further enhance our understanding of N-glycosylation in BC metastasis; these changes could be combined with complementary on-tissue digestion protocols using trypsin and collagenase with MALDI-MSI to evaluate the ECM composition (47).

In summary, MALDI-MSI combined with deep pathology analysis enabled extensive evaluation of N-glycosylation patterns in metastatic BC. Together, these data demonstrate that MALDI-MSI is a powerful molecular discovery tool for identifying therapeutic targets, validating diagnostic assays, and enhancing our basic biological understanding of disease processes. MALDI-MSI is also emerging as a valuable tool for molecular pathology evaluation, which delivers chemical sensitivity and specificity combined with spatial analysis of tissue sections for unprecedented translational research opportunities.

## Methods

### Samples.

Rapid autopsies were performed on 17 patients with terminal metastatic BC (Table 1). Nomenclature for BC type and stage are provided in the Supplemental Methods, Section 3 and 4. Written consent was obtained at the time of death from the patient’s designated next of kin. Seventeen single-patient TMAs were created from paraffin tissue blocks of the patient’s archived PT and from both normal tissue and multiple metastatic tissues sampled at autopsy. These TMAs consisted of 99 cores, each measuring 1.4 mm in diameter. The structure of a typical TMA in our study is shown in Figure 1. Four to 5 cores (mean of 4.5) per tumor sample were placed on each TMA to minimize sampling error. These 17 TMAs contained a total of 153 control core biopsies, 148 PT core biopsies, and 1399 metastasis core biopsies derived from, in total, 49 different organs. The number of metastatic sites per patient ranged from 8 to 16.

### Sample preparation.

Tissue sections (5 μm thick) were cut from each TMA blocks and mounted onto an ITO-coated glass slide (Delta Technologies). Slides were stored at –80°C. Sample preparation entailed deparaffinization, rehydration, antigen retrieval, PNGaseF enzyme application, incubation, and α-Cyano-4-hydroxycinnamic acid matrix deposition. Detailed protocol is available in the Supplemental Methods, Section 5.

### MALDI-MSI acquisition.

MALDI-MSI of the TMAs in this study was acquired with a rapifleX MALDI TOF instrument (Bruker Daltonik) in reflectron positive ion mode in the mass range of *m/z* 700–3000 Da. The pixel size was set to 50 μm, and 300 spectra were averaged per pixel with a laser frequency of 5 KHz. We also acquired high mass resolution MALDI–Fourier transform ion cyclotron resonance (MALDI-FTICR) imaging data of a representative TMA (case #3, see Table 1) using a custom-designed 21T FTICR instrument (48) build around Velos Pro linear ion trap (Thermo Fisher Scientific) with MALDI imaging source (Spectroglyph) (49), which included MS/MS data-dependent acquisition (DDA). Detailed acquisition protocols are described in Supplemental Methods, Section 6.

### Pathological annotation and its integration with MSI data set.

After MSI, all slides were washed in 100% methanol for 2 minutes (Biosolve Chimie SARL) to remove the matrix and subjected to conventional H&E staining (for detailed protocol, see Supplemental Methods, Section 7). After drying, each slide was scanned by a digital slide scanner (Mirax) at 20× magnification. All H&E images were annotated by a board-certified pathologist in CaseViewer (v2.3) software (3DHISTECH). Detailed pathological explanation of each annotation category is presented in the Supplemental Methods, Section 1. In total, 9055 annotations were created for this data set, with 530 annotations on average per TMA. Then, for each TMA’s H&E image, all annotations were exported as XML files from CaseViewer and imported to MATLAB for coregistration with the MSI data set. Detailed step-by-step description of image coregistration between MALDI-MSI and H&E, and subsequent transfer of annotations from H&E files onto the corresponding MALDI-MSI data sets, can be found in the Supplemental Methods, Section 8.

### Data preprocessing.

All individual raw MALDI-MSI data sets (on average, 130,000 pixels) were imported from FlexImaging software (Bruker Daltonik) .mis files into SCiLS Lab software (SCiLS, Bruker Daltonik) with convolution (width 20) baseline correction resulting in 1 comprehensive SCiLS file containing all 17 data sets (2,328,548 pixels). Each pixel was normalized to its total ion count. The overall mean spectrum from all pixels was calculated and subjected to peak picking using the mMass software (50). First, Gaussian smoothing was applied to the spectrum and then deisotoping and peak-picking were applied with signal-to-noise intensity threshold of 3. This resulted in a peak list containing 42 *m/z*. This peak list was then imported into SCiLS and used for the entire 17 TMA data set. An attribute table containing TMA number, BC subtype, tissue, and annotation type was appended to every pixel/data point. An overview of the frequency of each tissue and annotation in the cohort is presented in Supplemental Table 1. Identification of glycans was performed using accurate mass measured in additional MALDI-MSI experiments from a representative TMA (case #3, see Table 1) using 21T MALDI-FTICR DDA imaging as described above. The GlycoWorkbench (v2.1) software was used to search the GlycomeDB database with default settings and to assign *m/z* values to their corresponding N-glycan identifications (51). The structures for each m/z of interest were then fragmented, and the MS/MS spectra were annotated with these fragments using GlycoWorkbench at a tolerance of 1.0 Da. Additionally, all identified glycans were cross-examined by searching the recent glycan literature (13, 52, 53). The consolidated annotation reports are available in Supplemental Tables 9–21. Annotated spectra were generated on GlycoWorkbench using the reporting tool and are available in Supplemental Figures 6–17. Detailed analysis protocols are available in Supplemental Methods, Section 6.

### GE analyses.

Data sets (GSE26338 and GSE110590; refs. 15, 17) were analyzed, comparing GE among normal, primary, and metastatic tissues. A heatmap was drawn to show the genes in GSE26338 whose expression differed significantly between PT and metastases (*P* < 0.05, FDR < 0.15). To analyze enrichment of cancer-related proteins among the N-glycosylated proteome, the dbPTM database (https://awi.cuhk.edu.cn/dbPTM/) was used to extract ~1300 unique human proteins that undergo N-glycosylation. Using the GSE26338 and the TCGA BC data set (25), we identified genes differentially expressed (FDR < 0.15) between normal breast and PT (TCGA) and PT and metastasis (GSE26338). Functional annotation was performed using the Enrichr R-package of the list of N-glycosylated proteins, with the WikiPathways database used as a library of pathway annotations and an enrichment FDR > 0.25 considered significant. The Human Cell Atlas (18) bone project and a single-cell RNA-Seq data set (GSE144568) were analyzed to identify cell-specific FUT8 expression. As described by Psaila et al. (19), after identification of highly variable genes, PCA and uniform manifold approximation and projection were performed. The expression of FUT8 in each cell type was identified. More details on the GE analyses are presented in the Supplemental Methods, Section 9.

### Integration of MALDI-MSI data with published IHC data.

IHC data of EPCAM from Cimino et al. (23) and FISH of cMYC amplification data from Singhi et al. (24) were retrieved. All data sets provided the average expression of a given molecule of interest in PT and in metastases for each patient. Average N-glycan profiles of metastasis and PT were created for each patient using the annotations “cancer” and “cancer with cancer-associated stroma”. For both the IHC and MSI data, differences between metastasis and PT for each patient were dichotomized using zero as a threshold. Differences between binary profiles of IHC markers and any single N-glycan were counted and tested for significance using a binomial test. *P* values were corrected using the Benjamini-Hochberg procedure.

### Statistics.

We exported the tissue and annotation types, which were contained in at least 5 patients/TMAs to ensure sufficient data coverage, to R (version 4.0.0) and averaged all regions of annotation *x* and tissue *y* (e.g., cancer, liver metastasis) to 1 value per TMA. Next, since less than 1% of all annotations contained < 150 spectra (pixels, data points) per annotation, they were removed to ensure high confidence. We counted how many TMAs contained each tissue-annotation combination, and we discarded combinations contained in < 5 patients/TMAs. Finally, we created all possible tissue-annotation combinations passing the number of spectra and TMAs required. Next, we looped through all 42 *m/z* values belonging to glycans and performed a 2-tailed paired *t* test for every *m/z*. The *P* value was subsequently adjusted for multiple testing by the Benjamini-Hochberg procedure. Adjusted *P* < 0.05 was considered significant. This loop was repeated for every tissue-annotation combination. PCA was performed within SCiLS. The input for the PCA were individual normalized and peak-picked MALDI-MSI spectra. The PCA was scaled using the unit variance method. Visualizations were created using either graphics within SCiLS or the circlize, ComplexHeatmap, and ggplot2 packages in R.

### Study approval.

Rapid autopsies were performed on 17 patients with terminal metastatic BC (Table 1). This study was approved by the appropriate IRB of the Johns Hopkins Hospital. Written consent was obtained at the time of death from the patient’s designated next of kin.

## Author contributions

KS contributed formal analysis, investigation, writing (original draft preparation), and visualization; OTA contributed data curation and writing (review and editing); BB contributed methodology, software, supervision, and writing (review and editing); VA contributed formal analysis and visualization; CMT contributed resources and writing (review and editing); NMJ contributed formal analysis and visualization; BSRC contributed formal analysis and visualization; APB contributed resources and investigation; AMCM contributed data curation and writing (review and editing); MJW contributed data curation and writing (review and editing); PA contributed conceptualization and writing (review and editing); RMAH contributed conceptualization, writing (review and editing), supervision, and funding acquisition; and KG contributed conceptualization, writing (review and editing), supervision, and funding acquisition.

## Supplementary Material

Supplemental data

Supplemental tables 1-9

## Figures and Tables

**Table 1:**
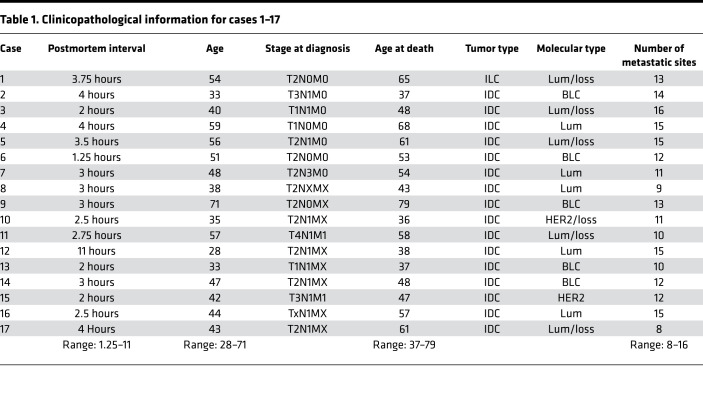
Clinicopathological information for cases 1–17

## Version 1 11/09/2021

In-Press Preview

## Version 2 12/22/2021

Electronic publication
