# Supplementary material for: Clinical importance of high-mannose, fucosylated, and complex N-glycans in breast cancer metastasis
Source: JCI Insight. 2021 Dec 22;6(24):e146945. doi: 10.1172/jci.insight.146945 (PMC8783675; doi:10.1172/jci.insight.146945)
Supplement: Supplemental data [file jciinsight-6-146945-s172.pdf]

Supplemental Information

Clinical importance of high-mannose, fucosylated, and complex N-glycans in breast cancer metastasis

Authors: Klára Ščupáková[1], Oluwatobi T. Adelaja[2], Benjamin Balluff[1], Vinay Ayyappan[2], Caitlin M. Tressler[2], Nicole M. Jenkinson[2], Britt S.R. Claes[1], Andrew P. Bowman[1]Ashley M. Cimino-Mathews[3,4], Marissa J. White[3], Pedram Argani[3,4], Ron M.A. Heeren[1], and Kristine Glunde[2,4]*

Affiliations:

[1]Maastricht MultiModal Molecular Imaging Institute (M4I), Maastricht University, The Netherlands

[2]The Russell H. Morgan Department of Radiology and Radiological Science, Division of Cancer Imaging Research, The Johns Hopkins School of Medicine, Baltimore, Maryland, United States

[3]Department of Pathology, The Johns Hopkins School of Medicine, Baltimore, Maryland, United States

[4]The Sidney Kimmel Comprehensive Cancer Center, The Johns Hopkins School of Medicine, Baltimore, Maryland, United States

[5]Department of Biological Chemistry, The Johns Hopkins School of Medicine, Baltimore, Maryland, United States

<u>* Corresponding authors:</u>
Kristine Glunde, Ph.D.
The Johns Hopkins University School of Medicine
Russell H. Morgan Department of Radiology and Radiological Science
Division of Cancer Imaging Research
720 Rutland Avenue
Traylor Building, Room 203
Baltimore, MD 21205
U.S.A.
Phone: (410) 614-2705
Email: kglunde@mri.jhu.edu

R.M.A. Heeren, Ph.D.
Director M4I
Division Head Imaging Mass Spectrometry
Minderbroedersberg 4-6
6211 LK Maastricht
The Netherlands
Phone: +31 43 388 2222
Email: r.heeren@maastrichtuniversity.nl

Keywords: Breast cancer, glycosylation, molecular imaging, molecular pathology, mass spectrometry imaging

Running Title: Clinical importance of N-glycans in metastatic breast cancer

Conflict of Interest: The authors have declared that no conflict of interest exists.

Supplemental Methods

1. Pathology characterization of annotation categories

Annotation	Description
1. Normal	Normal histological variant; non-neoplastic and native to the anatomic site
2. Stroma	Hematopoietic cells + fibroblasts + blood vessels; Non-neoplastic and native to the anatomic site
3. Cancer-associated stroma	Extracellular matrix + fibroblasts + blood vessels; Type of Stroma: desmoplastic, fibrous, or hyalinized
4. Cancer	Glandular pattern carcinoma; Neoplastic
5. Cancer mixed with cancer-associated stroma	Glandular pattern carcinoma; Neoplasm + Extracellular matrix + fibroblasts + blood vessels<br>Type of Stroma: desmoplastic, fibrous, or hyalinized
6. Necrosis	Irreversible cell injury

Note: H&E images below show examples of each category for a breast tissue. Other tissues within this multi-organ cohort were annotated similarly.

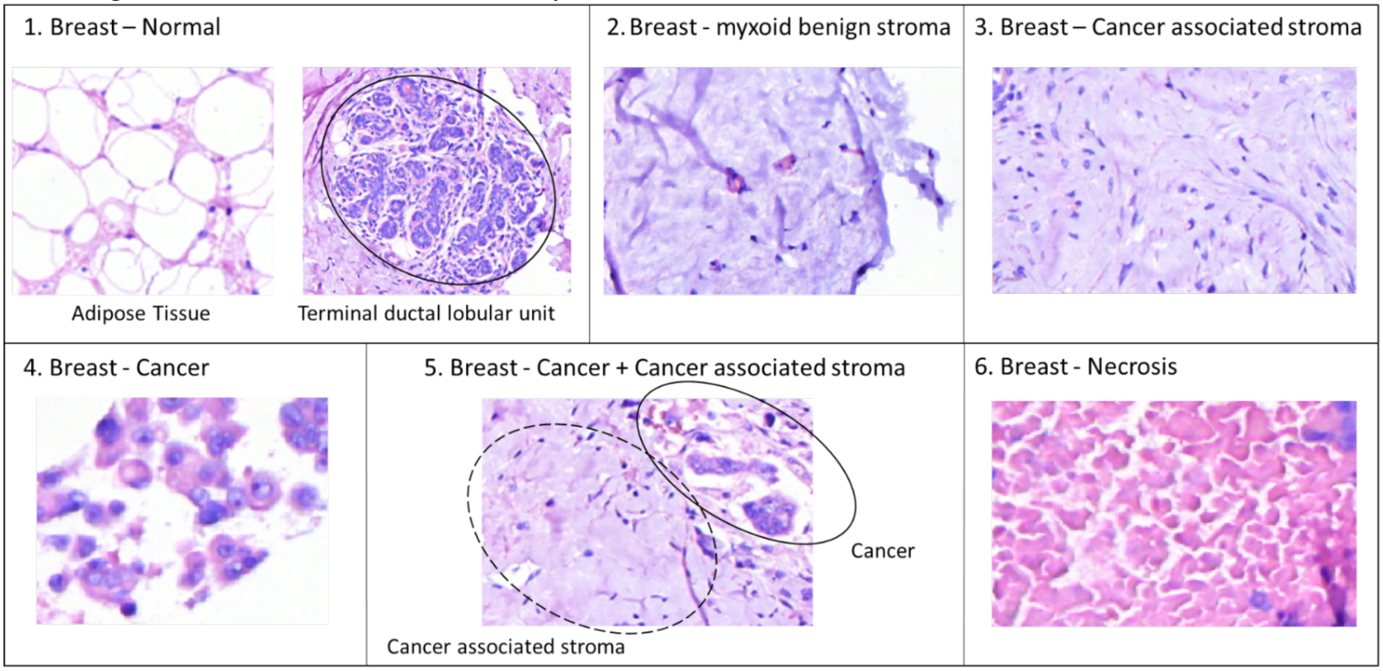

2. N-glycan nomenclature and symbolism

Throughout our publication, we used the standardized nomenclature and symbolism for glycans (1, 2). In the following, we present a brief summary of the terminology and symbols.

Supplemental scheme: Overview of generic and specific monosaccharide names and symbols.

General sugar structure	Specific sugar structure				
	Glucose	Mannose	Galactose	Fucose	Sialic acid
Hexose	Glc	Man	Gal		
HexNAc (N-Acetylhexosamine)	GlcNAc	ManNAc	GalNAc		
Deoxyhexose				Fuc	
Di-deoxynonulosonate					Neu5Ac

An example of N-glycan nomenclature:

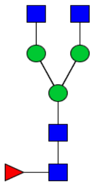

Hex3dHex1HexNAc4 – this naming reflects the general monosaccharide content, i.e., 3 hexoses, 1 deoxyhexose and 4 N-acetyl hexosamines.

3. BC molecular subtypes

Breast cancers (BC) are classified in several ways. The most common subtyping of clinical BC is based on a few key genes that the cancer does or does not express (3). The main subtypes are:

Luminal (Lum)
Luminal tumors are estrogen receptor–positive (ER-positive), and often progesterone receptor–positive (PR-positive), but HER2 receptor–negative (HER2-negative). Since we are studying primary tumor as well as distant metastases, we further distinguish the following 2 notations: Lum and Lum/loss. Lum means that the primary as well as the distant metastatic tumors are ER-/PR-positive, whereas Lum/loss signifies that the primary tumor had ER-/PR-positive status, but the distant metastases lost ER/PR positivity.

HER2-positive (HER2+)
HER2-positive breast cancer is hormone-receptor negative (ER- and PR-negative) and HER2-positive. Similarly, we also have Her2+ and Her2+/loss types, where Her2+ means ER- and PR-negative and Her2+ in primary as well as metastatic tumors, while Her2+/loss indicates the loss of Her2+ in distant metastases.

Basal-like (BLC)
The last molecular subtype is basal-like or so-called triple-negative breast tumors. These tumors are negative for hormone-receptors (ER and PR) and HER2.

4. BC stage and tumor type

Stage at diagnosis determined by TNM scoring
Clinical assessment of the tumor size and spread is reported using the TNM (**T**umor, lymph **N**ode, **M**etastasis) scoring system. We used this classification in Supplemental Table 1 detailing the clinicopathological information for each case (main text).

The number following the letter T stands for the size of the tumor.	
T1	≤2 cm across
T2	2–5 cm across
T3	>5 cm across
T4	Tumor of any size with direct extension to the chest wall and/or to the skin
TX	Size could not be assessed
The number following N describes the degree of cancer spreading to the lymph nodes.	
N0	No lymph nodes affected
N	Cancer cells are in the lymph nodes in the armpit but the nodes are not attached to surrounding tissues
N2	Cancer cells in the lymph nodes in the armpit, which are attached to each other and to other structures
N3	Cancer cells in the lymph nodes below/above the collarbone and/or armpit lymph node and/or behind the breastbone
NX	Lymph nodes could not be assessed for cancer cells
The number following the letter M indicates the spread of cancer cells to distant organs.	
M0	No distant organs involved
M1	Cancer has spread to another part of the body
MX	The spread of cancer cells to distant organs could not be assessed

Tumor type (4)
In the studied patient cohort, two types of primary breast cancer (Supplemental Table 1) occurred: infiltrating lobular carcinoma (ILC) and infiltrating ductal carcinoma (IDC). ILC starts in the lobules (milk glands) of the breast and often metastasizes to other organs. ILC accounts for 10% to 15% of all breast cancers. IDC begins in the milk ducts of the breast, then penetrates the ductal wall, invades the surrounding fatty breast tissue, and eventually may spread to other parts of the body. IDC is the most common type of breast cancer, accounting for 80% of breast cancer diagnoses.

5. Sample preparation for N-glycan MALDI-MSI analysis

For analysis, the glass slide was removed from the -80 °C freezer and dried in a vacuum desiccator for 30 min. The slide was subsequently placed on a heating plate set to 60 °C for 60 min. Next, the slide was deparaffinized by dipping it twice into 100% xylene (Biosolve Chimie SARL) for 10 min each, followed by a rehydration series of two 100% ethanol (Biosolve Chimie SARL,) washes for 2 min each and two milliQ water washes for 5 min each. The following step was antigen retrieval to reverse the protein cross-linking. For that purpose, the slide was submerged into 10 mM citric acid buffer at pH 6.0 and heated under pressure for 20 min using the Antigen Retriever 2100 (Aptum Biologics). After antigen retrieval, the slide was rinsed twice in milliQ water for 1 min each and dried using nitrogen gas. Next, 15 layers of

200 ng/µL water-dissolved PNGaseF (N-Zyme Scientifics) were applied to the slide using a SunCollect pneumatic sprayer (SunChrom GmbH) with a constant 10 µL/min flowrate, track spacing of 1 mm, nozzle height of 25 mm, and a nozzle speed of 900 mm/min. After enzyme application, the slide was incubated for 3 h at 37 °C in a humid chamber. Then, water-based Tipp-Ex (BIC S.A.) fiducial markers were placed on the glass slide and the slide was scanned at 2000 dpi resolution with desktop slide scanner Super CoolScan 5000 (Nikon). Afterwards, 8 layers of 5 mg/mL alpha-cyano-4-hydroxycinnamic acid (CHCA, Sigma-Aldrich) in 50% acetonitrile (Biosolve Chimie SARL) and 0.2% trifluoroacetic acid (Sigma-Aldrich, The Netherlands) were applied using HTX TM Sprayer (HTX Technologies) with 0.05 ml/min flowrate, 1100 mm/min velocity, 30 °C temperature, and 2 mm track spacing.

6. Acquisition and analysis of high mass resolution MALDI imaging data

We acquired high mass resolution MALDI imaging data on a Velos Pro linear ion trap (Thermo Scientific, San Jose, USA) equipped with a National High Magnetic Field Laboratory designed external linear quadrupole ion trap, quadrupole ion transfer optics, and a novel dynamically harmonized ion cyclotron resonance (ICR) cell, which is operated at 7.5 V trapping potential (5). The commercial ion source and stacked ring ion guide were replaced with an elevated-pressure MALDI ion source incorporating a dual-ion funnel interface (Spectroglyph LLC, Kennewick, USA) as previously described (6). The mass spectrometer was operated with an ion injection time of 250 ms and automatic gain control (AGC) was turned off. A transient duration of 3.1 s was used for ultra-high mass resolving power analyses, resulting in a 4 s acquisition time per-pixel. Tandem MS data-dependent acquisition (DDA) of the samples were acquired on sequential pixels, with both operated at 3.1 s transients. MS^1 mass spectra were captured between 500-2000 *m/z* in positive ion mode, with MS^2 spectra acquired from 200-2000 *m/z*. Samples were post-acquisition recalibrated in the MS^1 domain, using the known CHCA peak clusters available within this spectral window, while MS^2 spectra were not recalibrated. To enable ease of use during data interpretation, MS^1 *m/*z windows were shortened to 850-2000 after recalibration to significantly decrease data size. Data were peak picked in mMass (mmass.org) and loaded into Glycoworkbench for further analysis. The GlycoWorkbench (v2.1) software was used to search the GlycomeDB database with default settings and assign m/z values to their corresponding N-glycan identifications. Default settings were as follows: derivatization - underivatized (und), reducing end - freeEnd, fragment types – B, C, Y, Z, maximum number of cleavages – 2, maximum number of cross rings – 1, derive options from parent ions – yes, maximum number of charges – 1, accuracy – 1.0 Da. The structures for each m/z of interest were then fragmented, and the MS2 spectra were annotated with these fragments using GlycoWorkbench at a tolerance of 1.0 Da. The consolidated annotation reports are available in Supplemental Tables 10-21. Annotated spectra were generated on GlycoWorkbench using the reporting tool and are available in Supplemental Figures 6-17.

7. H&E staining

Following MALDI-MSI measurement, the slides were submerged for 3 min each in a series of ethanol (Biosolve Chimie SARL) in water solutions in the order listed: 100% twice, 96% twice, and 70% twice. Next, the slides were washed for 3 min in water and submerged for 3 min in hematoxylin (Biosolve Chimie SARL). Next, the hematoxylin solution was washed off under running water and the slides were submersed in eosin (Merck KGaA) for 30 sec. The eosin was washed off under running water and the slides were submerged in 100% ethanol for 1 min and 100% xylene (Biosolve Chimie SARL) for 5 min. The final step was mounting of a glass cover slip using Entellan mounting medium (Merck KGaA) followed by drying for at least 12 hours.

8. Co-registration of MALDI-MSI and annotations

Prior to each MSI experiment, an optical image of the specimen was taken and used to define the measurement area by loading it into the FlexImaging software (v 5.0, Bruker Daltonik) where a rough co-registration was performed between the optical image and the sample stage coordinate system of the mass spectrometer. Three manually selected control points provided by the fiducial markers visible both in a camera within the mass spectrometer accessed via the FlexControl software (v 5.0, Bruker Daltonik) and the optical image in FlexImaging, were employed for co-registration.

The optical image taken prior to acquisition thus defines the coordinate space for every MALDI-MSI dataset. Next, to facilitate the co-registration of the MSI data with the H&E image, both the optical image as well as the corresponding H&E image were imported into MATLAB R2018b (MathWorks). There, the high-resolution H&E image was co-registered to the optical image by using at least three manually selected control points based on prominent morphological features of the tissue that were visible in both, H&E and optical scanner image (MATLAB command cpselect), resulting in the affine geometric transformation **Δ**HE-optical (MATLAB command fitgeotransform). Then, the annotations from CaseViewer were imported from the previously exported and corresponding XML file. The coordinates of each annotation were then transformed by applying the geometric transformation **Δ**HE-optical to the coordinate space of the MALDI-MSI dataset. These were finally written into the .mis file (Bruker data file) as "Region of interest".

9. Details of GE analyses

Gene expression of N-glycan biosynthesis genes

To determine whether expression profiles among genes associated with N-glycosylation were consistent with the glycosylation profiles observed by MALDI-MSI, two publicly available datasets (GSE26338 (7), GSE110590 (8)) were analyzed, comparing gene expression among normal, primary, and metastatic tissues. In GSE26338 (7), expression profiling was done by Agilent-012097 Human 1A Microarray (V2) G4110B of unmatched breast tissue, primary tumor, and metastatic tissue. In GSE110590 (8), expression profiling was done by high-throughput sequencing (Illumina HiSeq 2000) of matched primary breast tumor and metastatic tissue. Expression of aforementioned genes were analyzed in lymph, adrenal, brain, bone, liver, lung metastases, and primary tumor from 15 patients. The following samples were analyzed from GSE26338, including 5 normal breast, 18 primary tumor, and 25 metastases: GSM34427, GSM34440, GSM34441, GSM34465, GSM34490, GSM34476, GSM34477, GSM34491, GSM34492, GSM34504, GSM34505, GSM34506, GSM34508, GSM34512, GSM34514, GSM34523, GSM34527, GSM34528, GSM50154, GSM53475, GSM53476, GSM53477, GSM80245, GSM80246, GSM34442, GSM34464, GSM50147, GSM50148, GSM52910, GSM52911, GSM52912, GSM52914, GSM52915, GSM52917, GSM52919, GSM52920, GSM52921, GSM52922, GSM80213, GSM81165, GSM635382, GSM635389, GSM635391, GSM635398, GSM635403, GSM635406, GSM635407, GSM635409.

Cell specific gene expression of FUT8

To assess the native expression of FUT8 in the bone marrow microenvironment, the Human Cell Atlas (9) bone marrow tissue project, which comprises >100,000 hematopoietic cells sourced from eight healthy donors, was analyzed to identify the distribution of FUT8 expression across cell-types. In addition, a second single-cell RNA sequencing dataset (GSE144568) wherein 10 × scRNA sequencing had been performed on 135,929 CD34+ lineage hematopoietic stem and progenitor cells was analyzed. As described by Psaila *et al.* (10), after identification of highly variable genes by fitting a gamma-generalized linear model and correction of batch effects via the sva package in R (v4.0.0), principal component analysis and uniform manifold approximation and projection (UMAP) were performed. The expression of FUT8 in each cell type was identified and superimposed on a plot of the UMAP embedding. Annotations of cell

type were determined by labels established by Psaila *et al.* (10). Analyses of these datasets were performed using R, with the Seurat package used for analysis of single cell data.

Functional characterization of the N-glycosylated proteome

To analyze enrichment of cancer-related proteins among the N-glycosylated proteome, the dbPTM database was used to extract ~1300 unique human proteins that display N-glycosylation. Using the aforementioned datasets from the GEO, as well as the TCGA Breast Cancer dataset (11), genes differentially expressed (FDR < 0.15) between normal breast and primary tumor (TCGA) and between primary tumor and metastasis (GSE26338) were identified. A list of N-glycosylated proteins encoded by those differentially expressed genes was subsequently compiled. Moreover, functional annotation of the list of N-glycosylated proteins was performed using the Enrichr package in R, with the WikiPathways (WP) database used as a library of pathway annotations and an enrichment FDR > 0.25 considered significant. For analysis of the N-glycosylated proteome, dbPTM was used to obtain a curated list of proteins with available evidence of N-glycosylation. A second database, GlycoProtDB, was used to obtain information pertaining to the lectins to which each protein binds. Analysis of TCGA samples was performed by comparing expression of genes encoding N-glycosylated proteins between normal tissue and primary tumor samples from the TCGA Breast Cancer dataset (n=1211, including 114 normal breast and 1097 primary tumor samples). The differentially expressed genes that overlapped with the following WP datasets and gene ontology (GO) terms was selected for further analysis: Amplification and Expansion of Oncogenic Pathways as Metastatic Traits (WP), Matrix Metalloproteinases (WP), TGF-B Signaling in Thyroid Cells for Epithelial-Mesenchymal Transition (WP), Integrin-mediated Cell Adhesion (WP), Focal Adhesion (WP), Focal Adhesion-PI3K-Akt-mTOR-signaling pathway (WP), Extracellular Matrix Organization (GO), Positive Regulation of Cell Proliferation (GO), Regulation of Cell Migration (GO), Regulation of Cell Proliferation (GO), Extracellular Matrix Disassembly (GO), Positive Regulation of Cell Migration (GO), Cell-Cell Adhesion via Plasma-Membrane Adhesion Molecules (GO), Homophilic Cell Adhesion via Plasma Membrane Adhesion Molecules (GO), Heterotypic Cell-Cell Adhesion (GO), Regulation of Focal Adhesion Assembly (GO), Negative Regulation of Cell Motility (GO), Angiogenesis Involved in Would Healing (GO), Positive Regulation of Cell-Matrix Adhesion (GO), Positive Regulation of Adherens Junction Organization (GO), Positive Regulation of Epithelial Cell Migration (GO), Regulation of Extracellular Matrix Organization (GO), yielding 224 genes. Genes among this list represented among the differentially expressed genes in GSE26338 were considered for subsequent analysis. Kaplan-Meier curves were drawn using data from TCGA samples hosted via the open-source functional genomics explorer, Xena (12). Among the N-glycosylated proteins encoded by genes upregulated in metastasis, EPCAM was selected for further analysis. Boxplots were drawn to display differences in expression of EPCAM among normal tissue, primary tumor, and metastasis (GSE26338), and specifically among primary tumor and lymph, bone, brain, liver, and lung metastases (GSE110590).

References

1. Varki A, et al. Symbol Nomenclature for Graphical Representations of Glycans. *Glycobiology.* 2015;25(12):1323-4.
2. Neelamegham S, et al. Updates to the Symbol Nomenclature for Glycans guidelines. *Glycobiology.* 2019;29(9):620-4.
3. Dai X, et al. Breast cancer intrinsic subtype classification, clinical use and future trends. *American Journal of Cancer Research.* 2015;5(10):2929-43.
4. Sharma GN, et al. VARIOUS TYPES AND MANAGEMENT OF BREAST CANCER: AN OVERVIEW. *Journal of Advanced Pharmaceutical Technology & Research.* 2010;1(2):109-26.
5. Hendrickson CL, et al. 21 Tesla Fourier Transform Ion Cyclotron Resonance Mass Spectrometer: A National Resource for Ultrahigh Resolution Mass Analysis. *J Am Soc Mass Spectrom.* 2015;26(9):1626-32.

6. Bowman AP, et al. Ultra-High Mass Resolving Power, Mass Accuracy, and Dynamic Range MALDI Mass Spectrometry Imaging by 21-T FT-ICR MS. *Anal Chem.* 2020;92(4):3133-42.
7. Harrell JC, et al. Genomic analysis identifies unique signatures predictive of brain, lung, and liver relapse. *Breast Cancer Res Treat.* 2012;132(2):523-35.
8. Siegel MB, et al. Integrated RNA and DNA sequencing reveals early drivers of metastatic breast cancer. *J Clin Investig.* 2018;128(4):1371-83.
9. Hay SB, et al. The Human Cell Atlas bone marrow single-cell interactive web portal. *Exp Hematol.* 2018;68:51-61.
10. Psaila B, et al. Single-Cell Analyses Reveal Megakaryocyte-Biased Hematopoiesis in Myelofibrosis and Identify Mutant Clone-Specific Targets. *Mol Cell.* 2020;78(3):477-92.e8.
11. The Cancer Genome Atlas Program - National Cancer Institute. https://wwwcancergov/tcga.
12. Goldman MJ, et al. Visualizing and interpreting cancer genomics data via the Xena platform. *Nat Biotechnol.* 2020;38(6):675-8.

Supplemental Figures

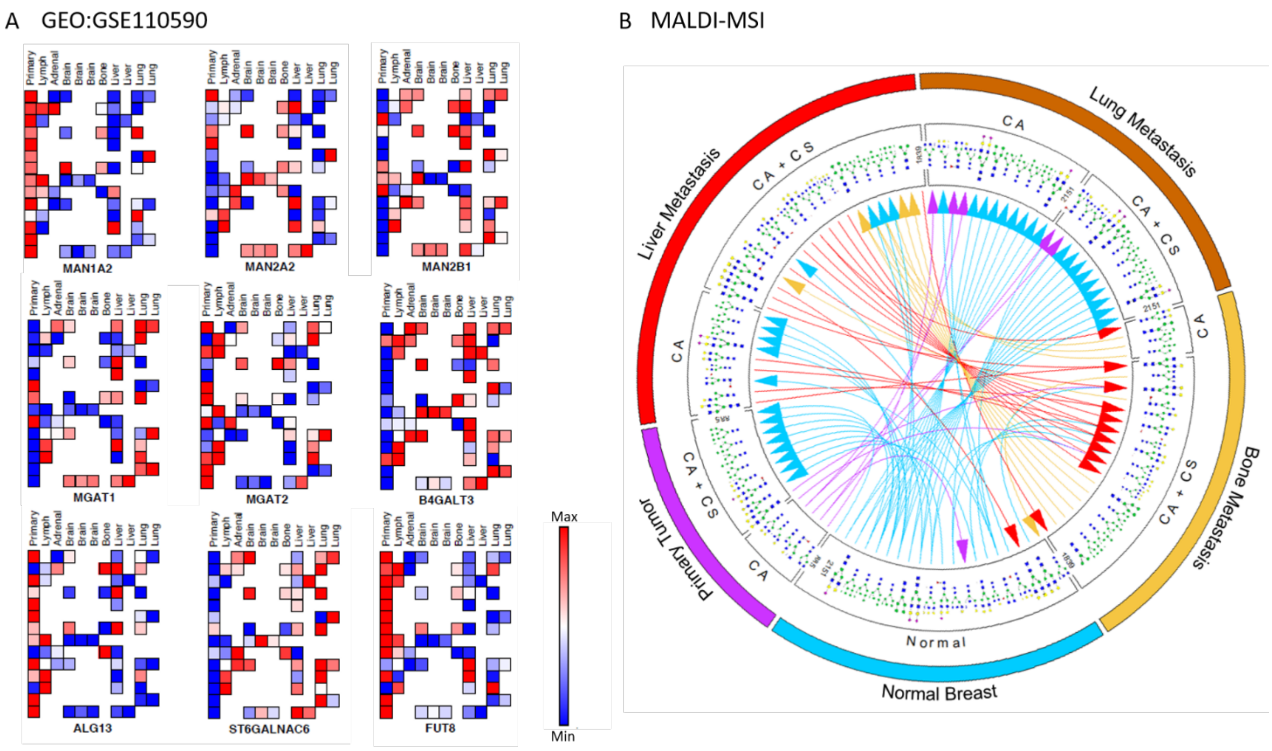

Supplemental Figure 1: Paired PT and organ-specific metastases mRNA sequencing data further supports gene alterations. (A) Publicly available dataset GEO:GSE110590 re-analyzed for N-glycosylation genes. Heat maps showing significantly altered genes between primary and metastatic tumors matched per patient ($p<0.05$, FDR < 0.15). **(B)** MALDI-MSI comparisons of N-glycan abundances between normal breast & primary tumor, and between bone, lung, and liver metastatic sites. Bone metastasis consists of pooled bone, spine, vertebra, and rib metastases. The arrows indicate increases in abundance relative to the color-coded tissue. CA – cancer, CA+CS – cancer with cancer-associated stroma.

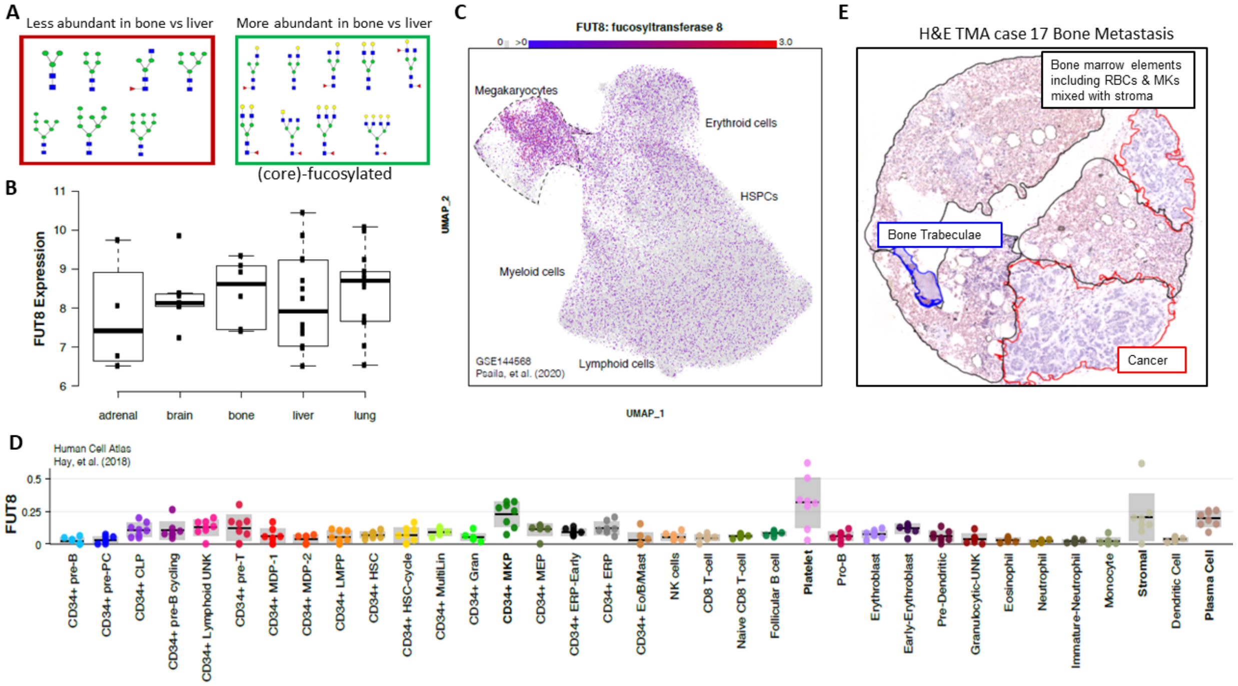

Supplemental Figure 2: Cell-type specific bone expression reveals preferential FUT8 expression by bone marrow elements. (A) N-glycans were significantly lower (red) or higher (green) in bone compared to liver metastases. Most of the abundant N-glycans in bone were fucosylated. **(B)** Publicly available dataset GEO:GSE110590 containing matched primary breast tumors with site-specific metastases re-analyzed for FUT8 expression per metastases. **(C)** Publicly available single-cell RNA sequencing dataset (GEO:GSE144568) analyzed to identify the distribution of FUT8 expression across cell types. Uniform manifold approximation and projection (UMAP) was performed and plotted to display the FUT8 cell type expression, with megakaryocytes having the highest one. **(D)** Human Cell Atlas and its bone marrow tissue project, containing >100,000 hematopoietic cells sourced from 8 healthy donors was analyzed to identify the distribution of FUT8 expression across cell types. **(E)** H&E staining of TMA #17 from our MALDI-MSI cohort, showing increased presence of megakaryocytes and red blood cells.

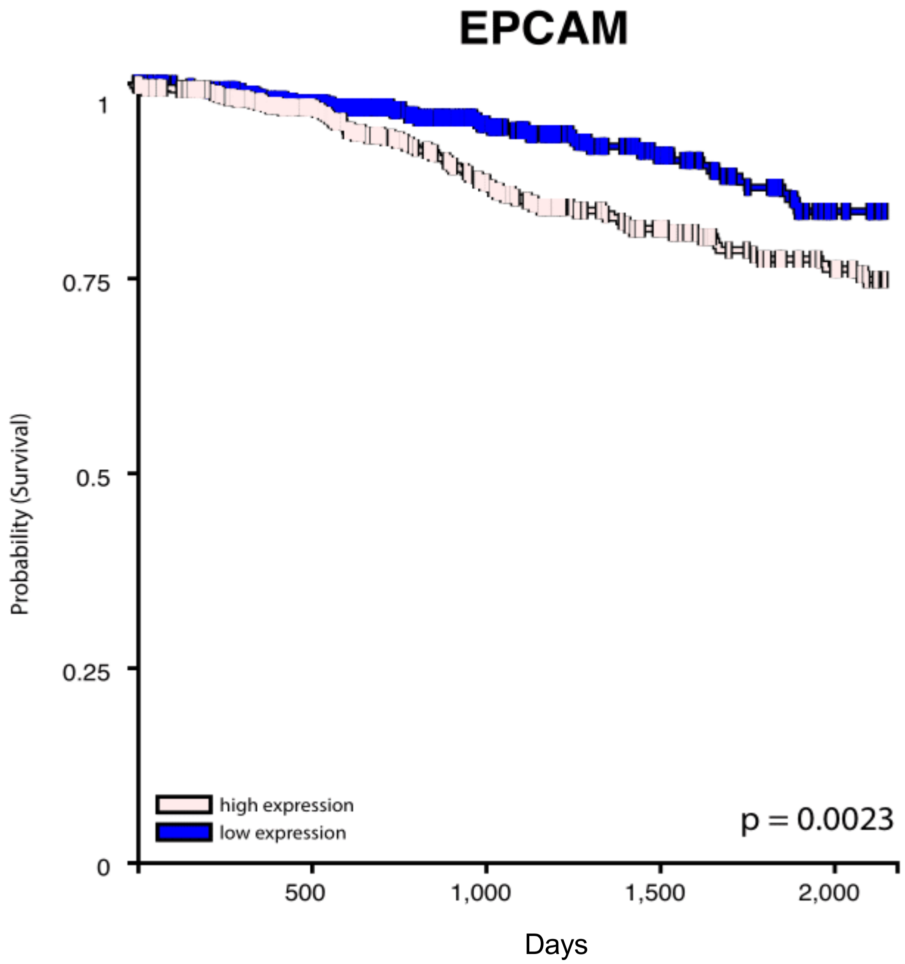

Supplemental Figure 3: Kaplan-Meier curve using the TCGA datatset of EpCAM (blue – lower expression) showing improved probability of survival with low EpCAM expression.

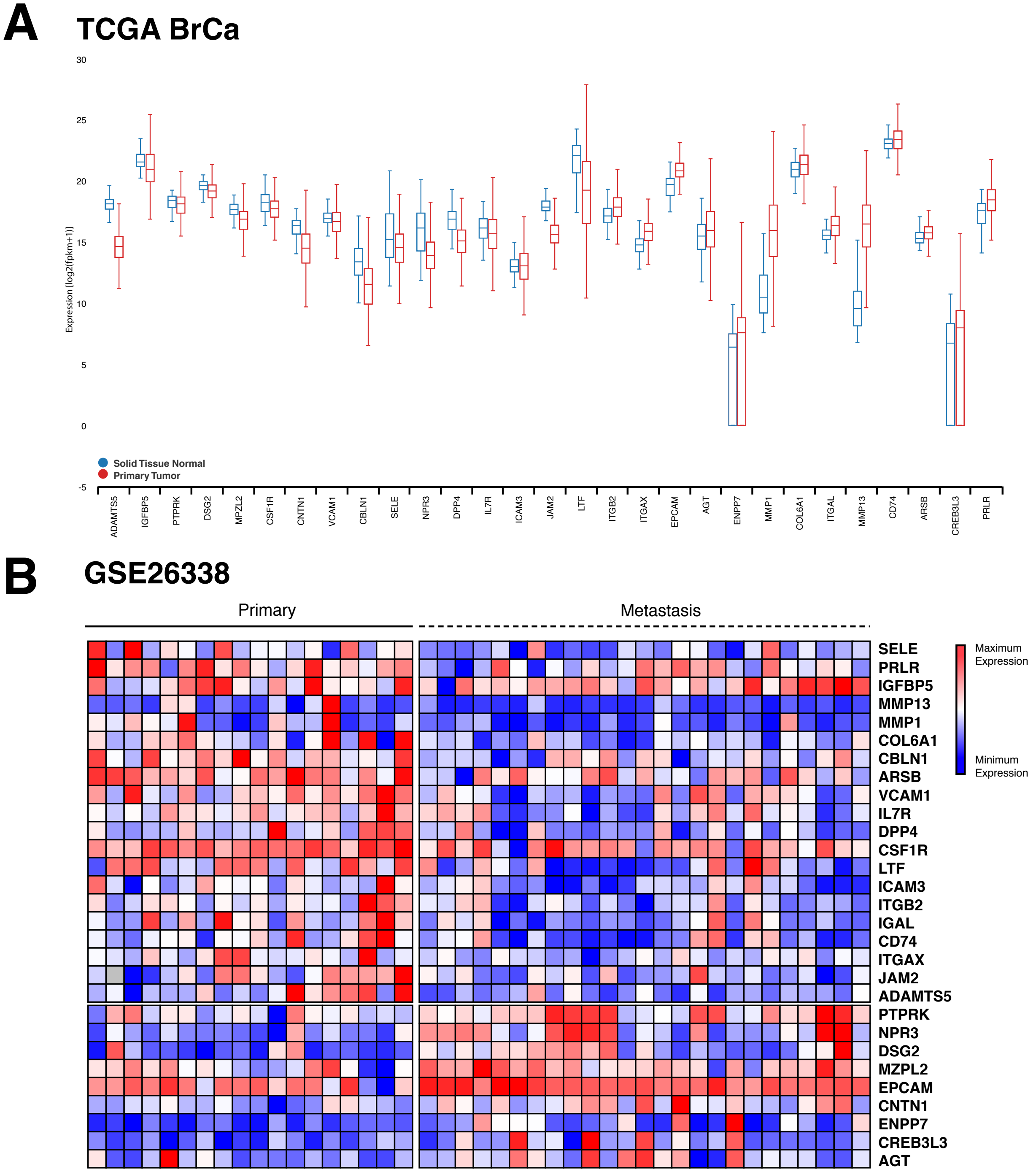

Supplemental Figure 4: Detailed GE of 29 metastasis-associated N-glycosylated and DE genes in BC. (A) GE of TCGA dataset, normal breast compared to PT and **(B)** GE of GSE26338 dataset, PT compared to metastasis.

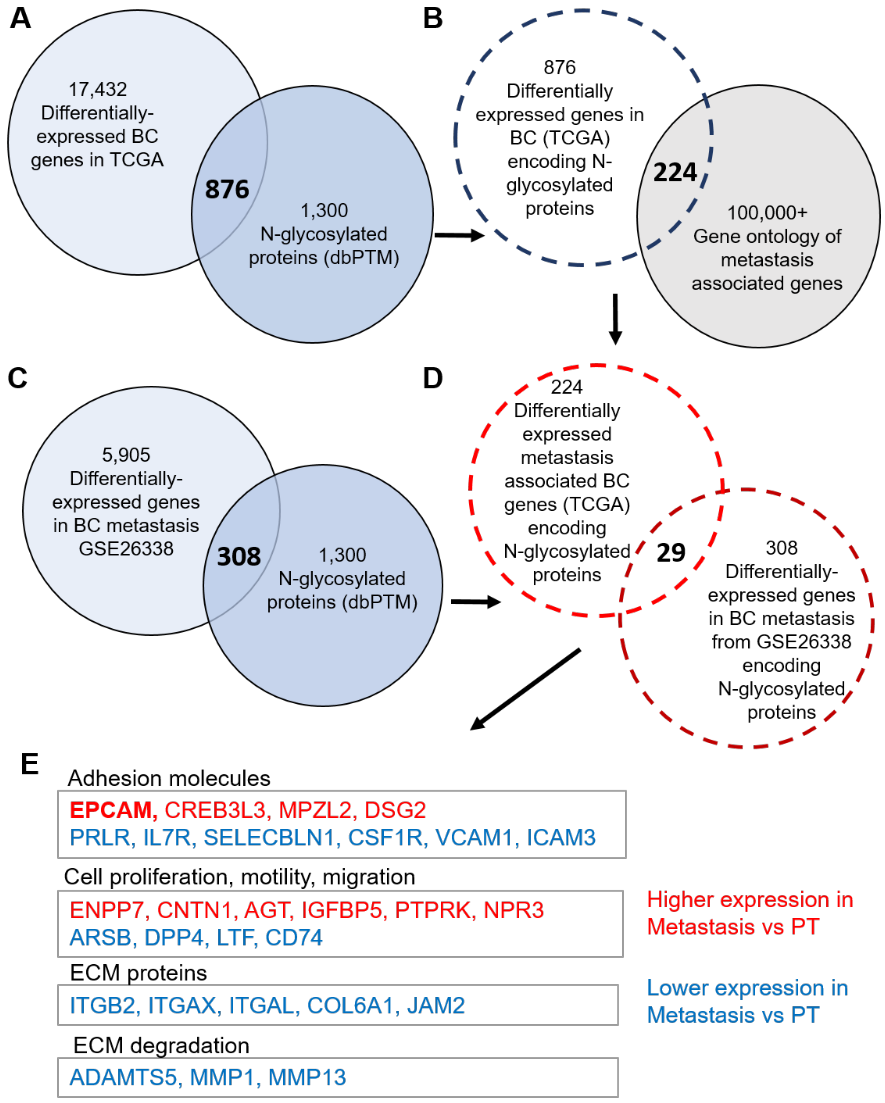

Supplemental Figure 5: Genes encoding N-glycosylated proteins are frequently differentially expressed (DE) in metastasis. (A) Overlap between DE genes in the TCGA breast cancer dataset and known N-glycosylated proteins from dbPTM. **(B)** Overlap between the DE genes encoding N-glycosylated proteins from A with the gene ontology term "metastasis". **(C)** Overlap between DE genes in GSE26338 dataset and known N-glycosylated proteins from dbPTM. **(D)** Overlap between DE genes encoding N-glycosylated proteins in GSE26338 in C and TCGA metastasis-associated genes encoding N-glycosylated proteins in B. **(E)** List of the overlapping genes encoding N-glycosylated proteins in D according to their function and color-coded by expression levels in metastasis compared to PT: red – higher, blue – lower.

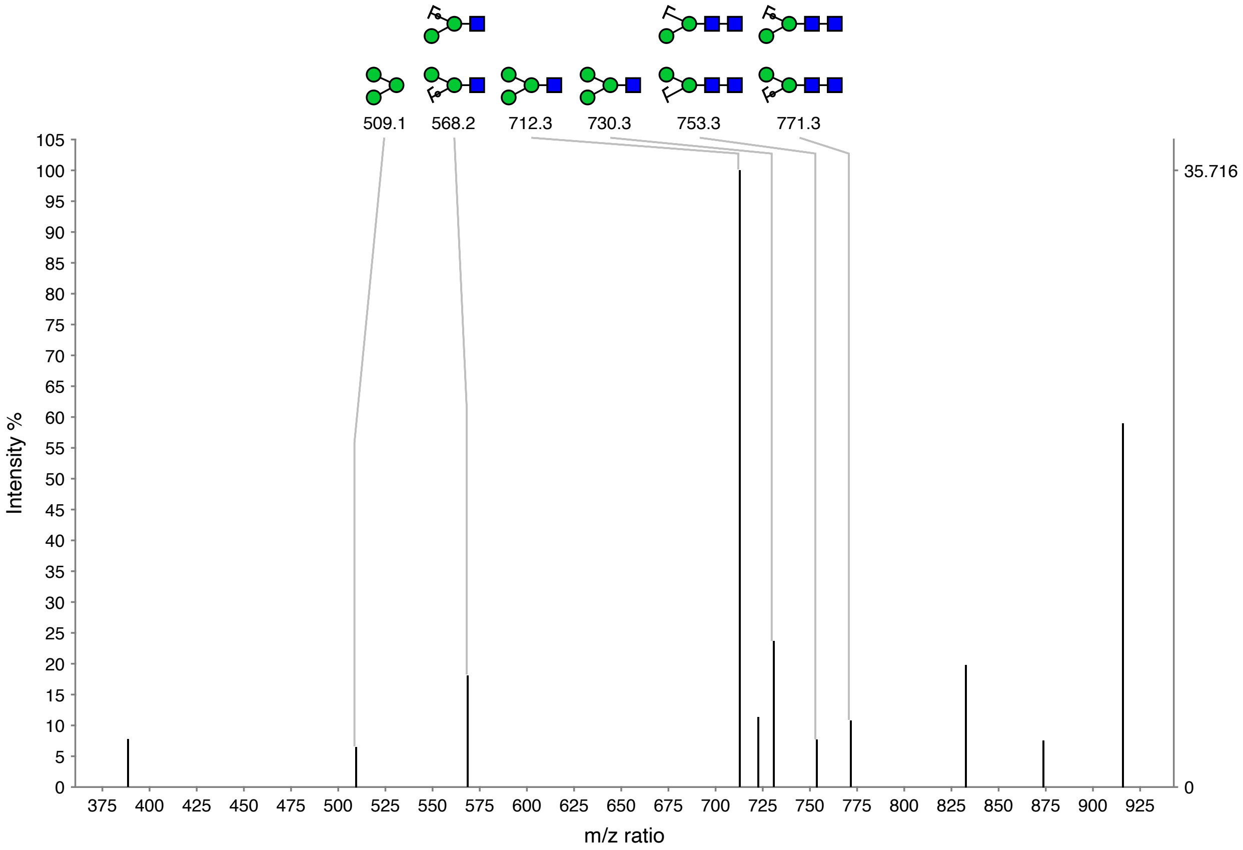

Supplemental Figure 6: Glycoworkbench generated annotation of 21T MALDI FTICR DDA fragmentation spectra of molecular ion m/z 933.31688. Fragmentation spectra was annotated based on the most relevant glycoform as circled in red in Supplemental Table 10, which was identified from our data and additional literature review.

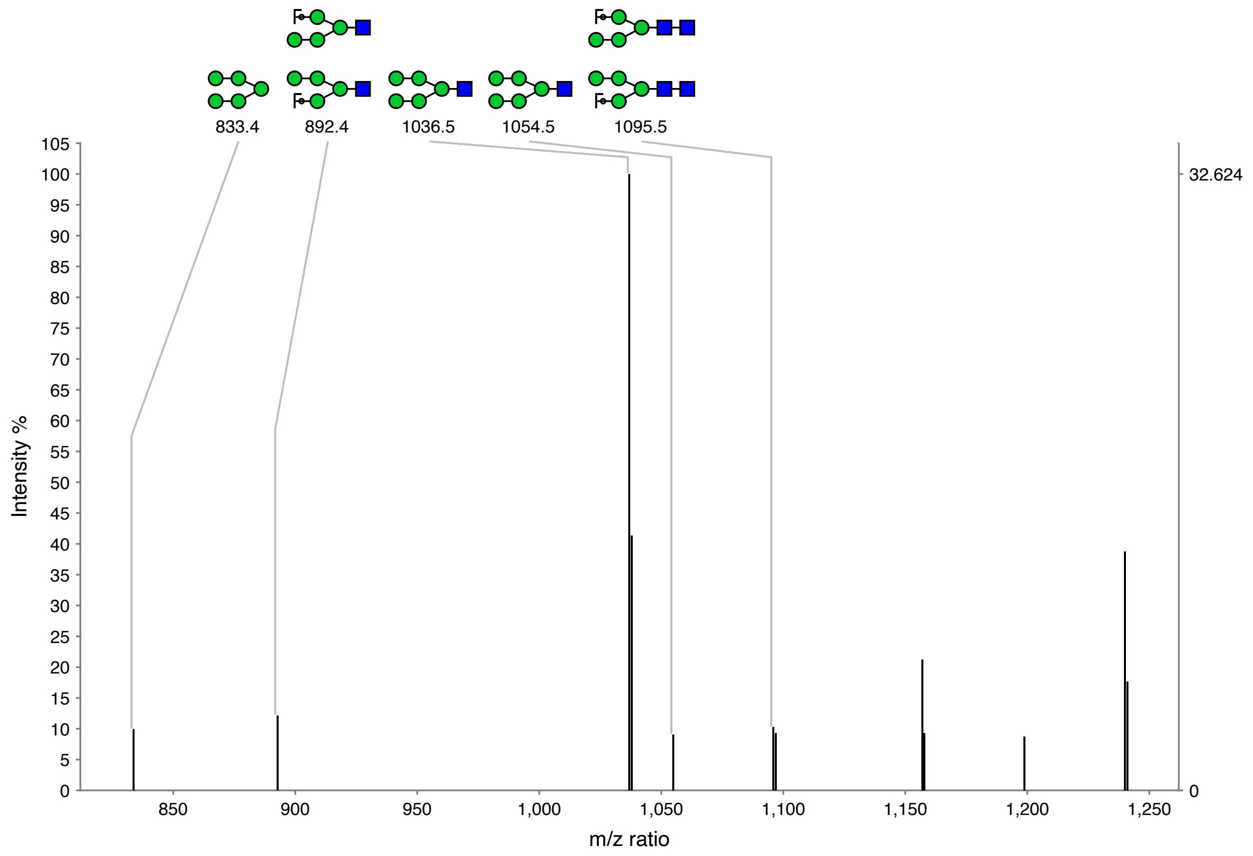

Supplemental Figure 7: Glycoworkbench generated annotation of 21T MALDI FTICR DDA fragmentation spectra of molecular ion m/z 1257.42247. Fragmentation spectra was annotated based on the most relevant glycoform as circled in red in Supplemental Table 11, which was identified from our data and additional literature review.

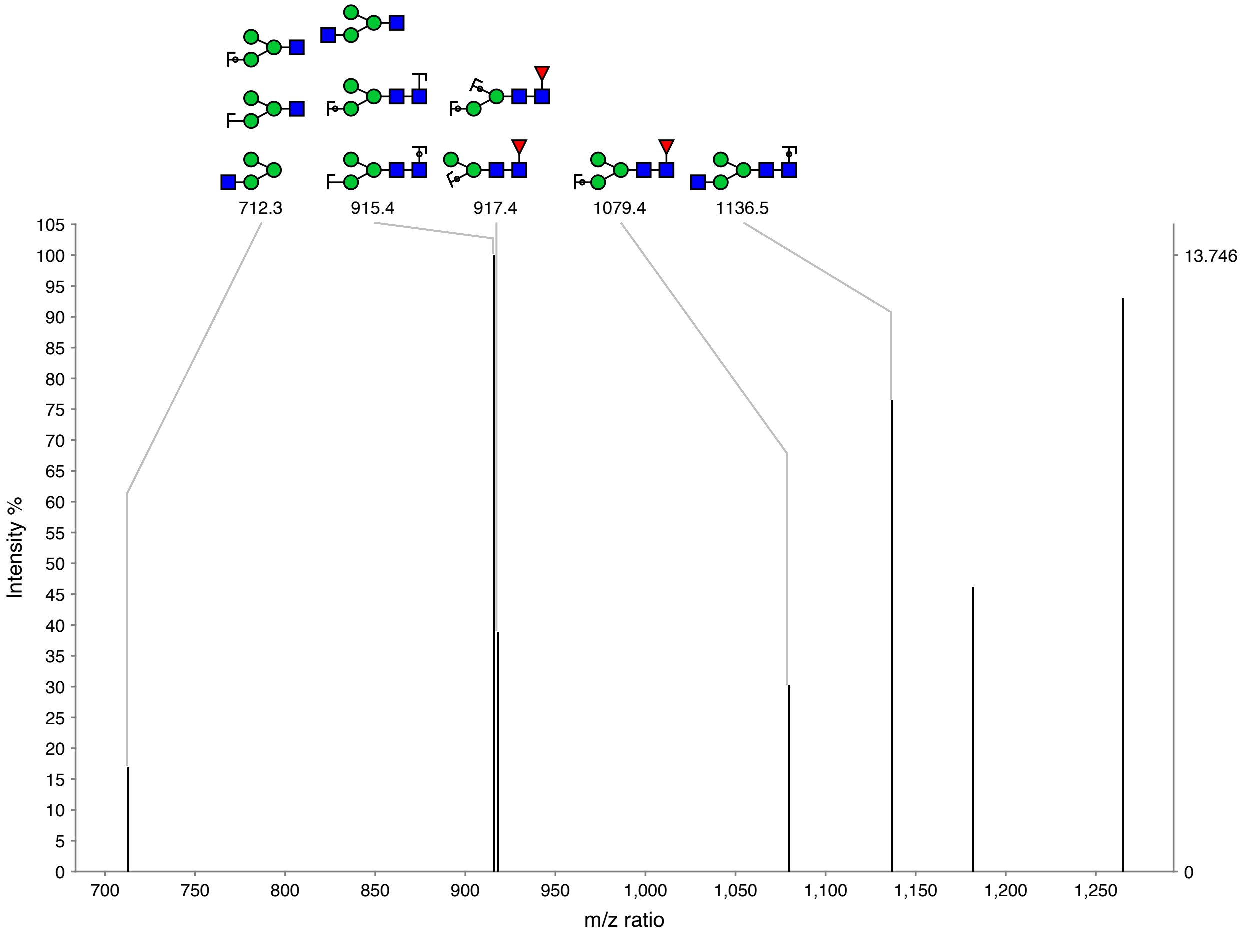

Supplemental Figure 8: Glycoworkbench generated annotation of 21T MALDI FTICR DDA fragmentation spectra of molecular ion m/z 1282.45432. Fragmentation spectra was annotated based on the most relevant glycoform as circled in red in Supplemental Table 12, which was identified from our data and additional literature review.

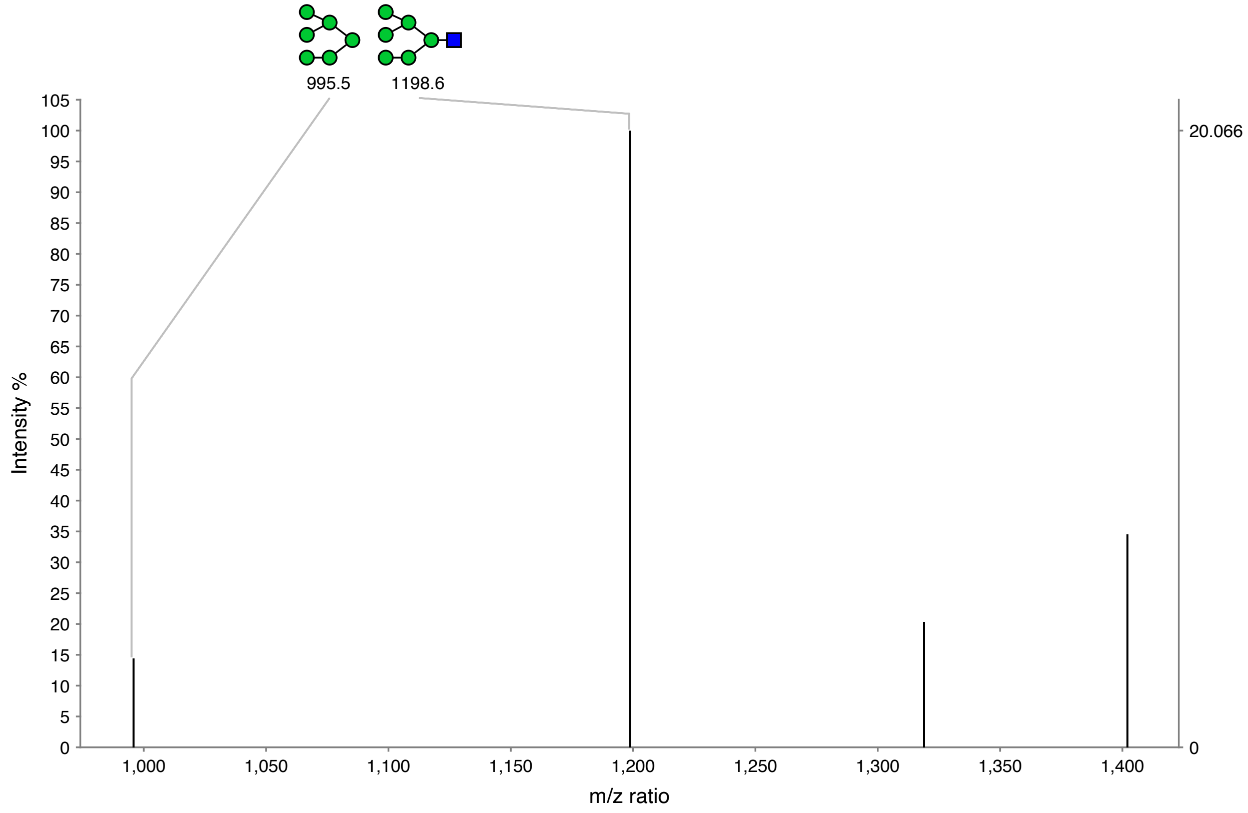

Supplemental Figure 9: Glycoworkbench generated annotation of 21T MALDI FTICR DDA fragmentation spectra of molecular ion m/z 1419.47530. Fragmentation spectra was annotated based on the most relevant glycoform as circled in red in Supplemental Table 13, which was identified from our data and additional literature review.

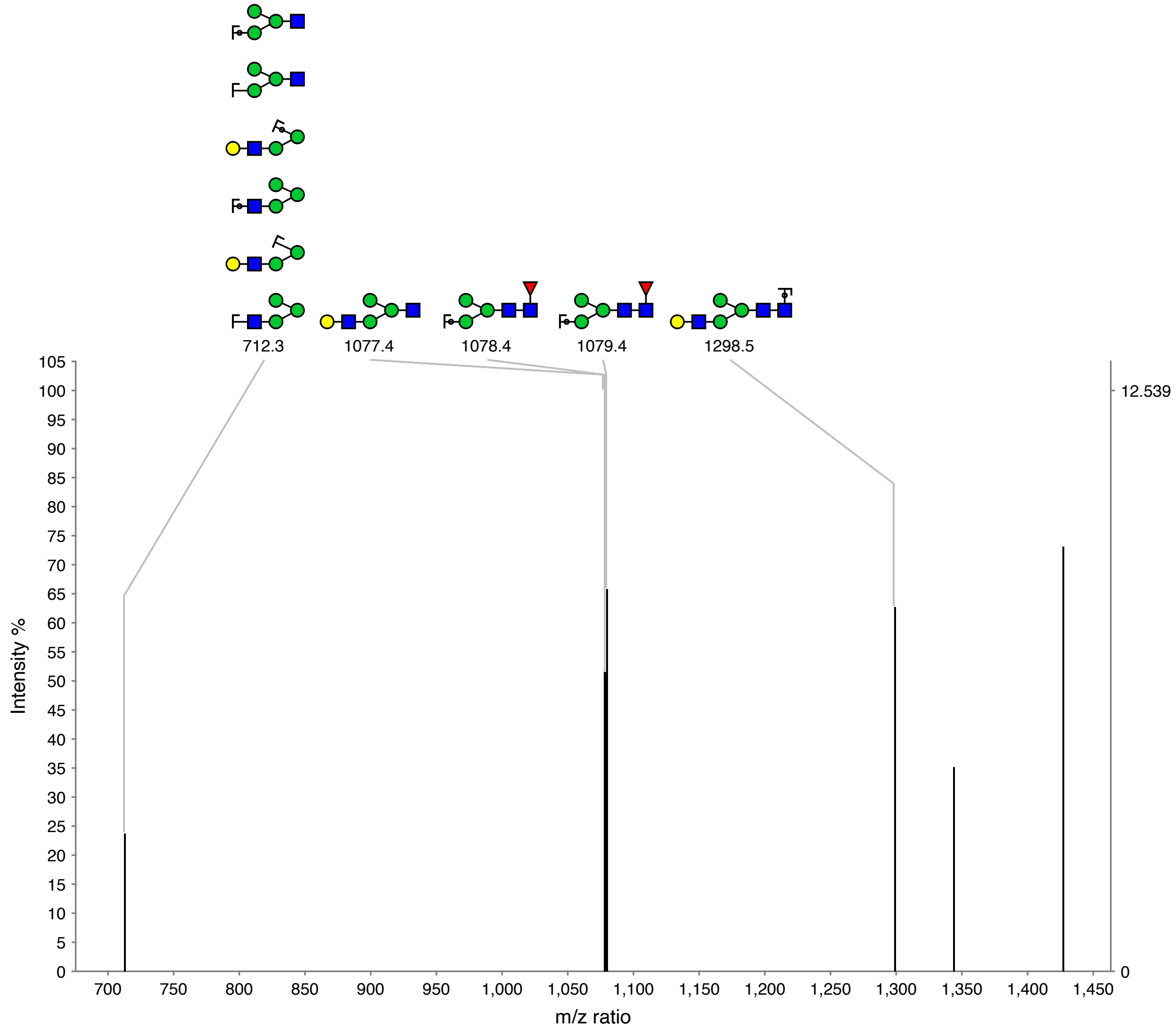

Supplemental Figure 10: Glycoworkbench generated annotation of 21T MALDI FTICR DDA fragmentation spectra of molecular ion m/z 1444.50788. Fragmentation spectra was annotated based on the most relevant glycoform as circled in red in Supplemental Table 14, which was identified from our data and additional literature review.

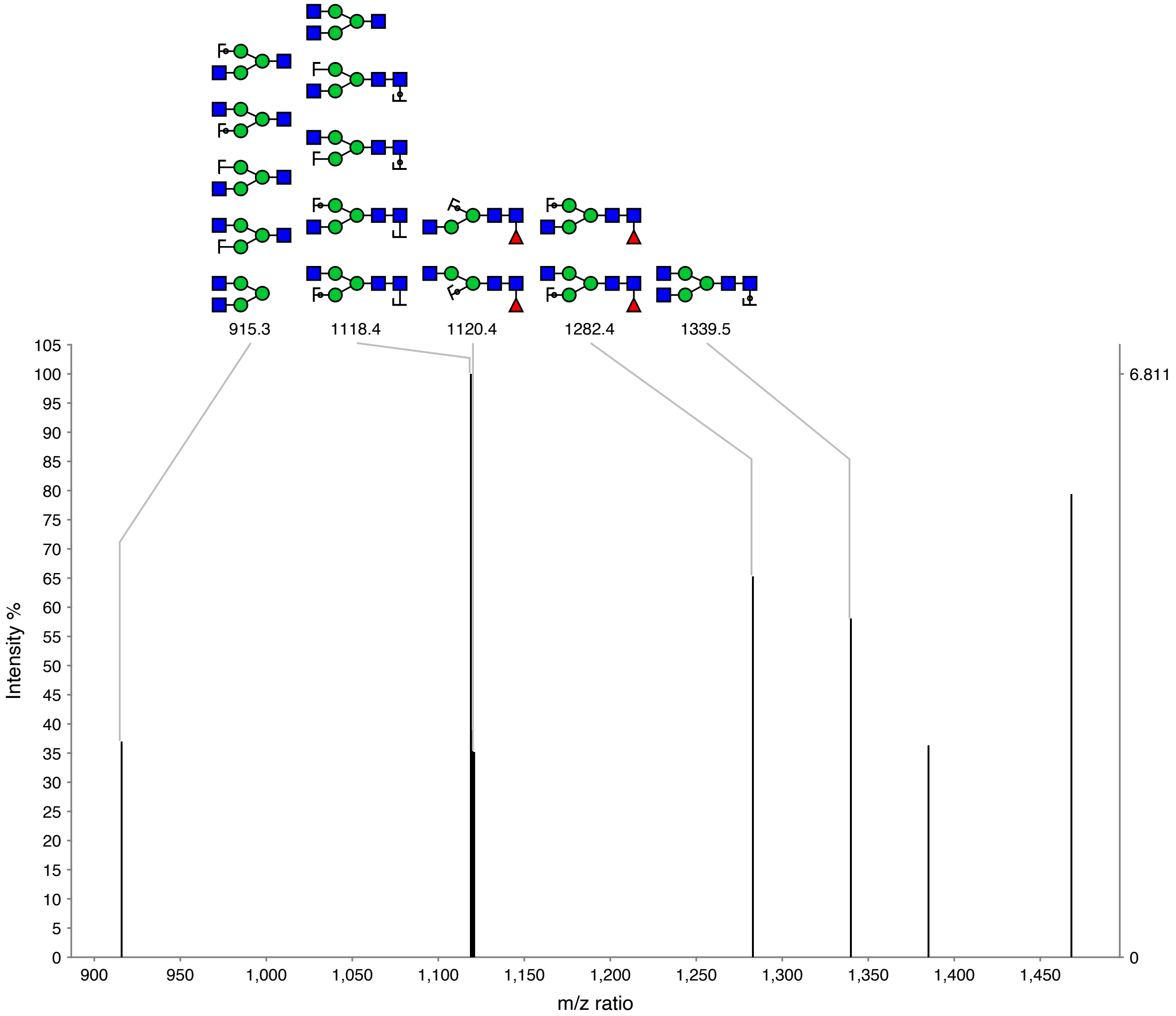

Supplemental Figure 11: Glycoworkbench generated annotation of 21T MALDI FTICR DDA fragmentation spectra of molecular ion m/z 1485.53418. Fragmentation spectra was annotated based on the most relevant glycoform as circled in red in Supplemental Table 15, which was identified from our data and additional literature review.

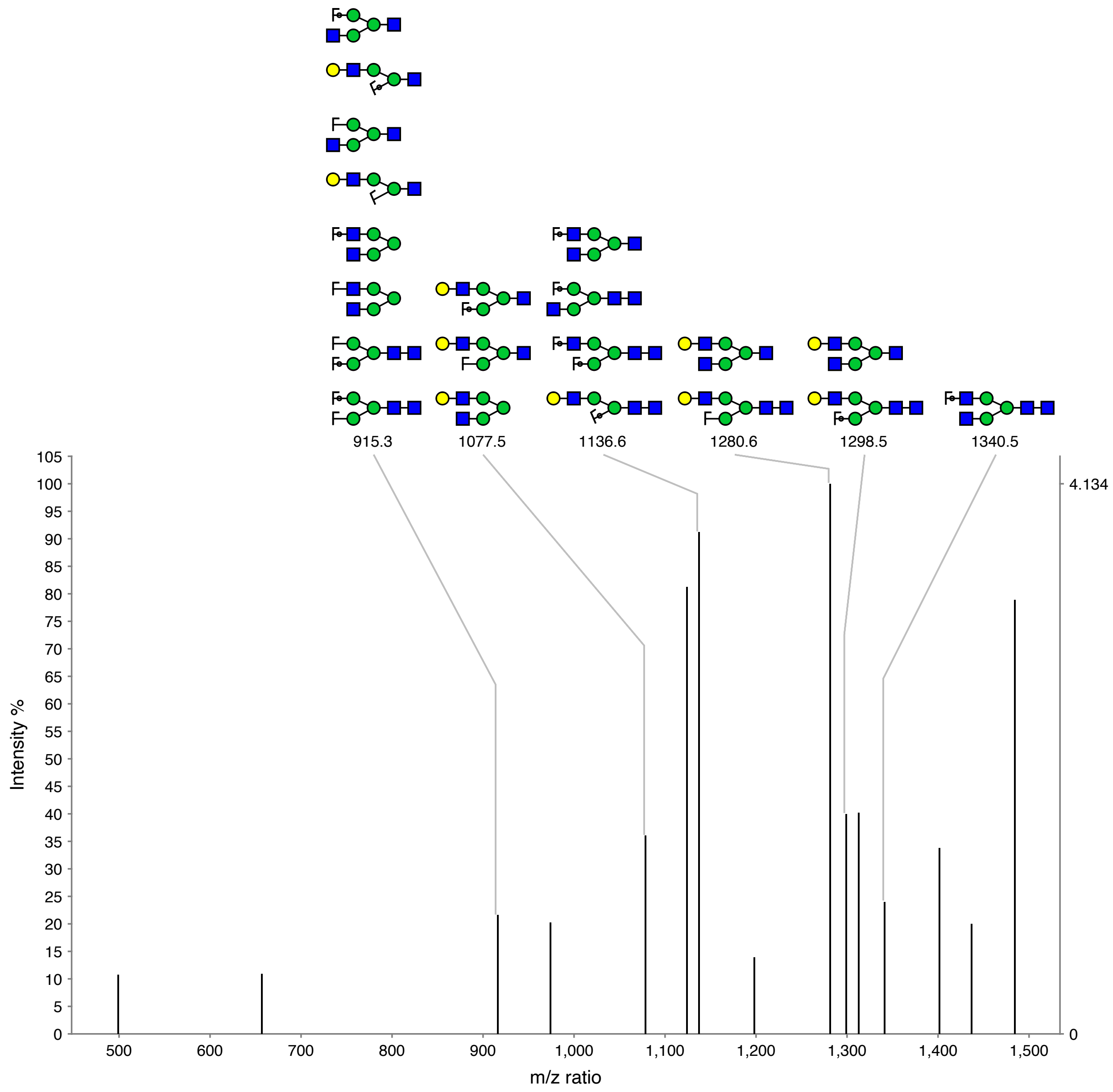

Supplemental Figure 12: Glycoworkbench generated annotation of 21T MALDI FTICR DDA fragmentation spectra of molecular ion m/z 1501.52837. Fragmentation spectra was annotated based on the most relevant glycoform as circled in red in Supplemental Table 16, which was identified from our data and additional literature review.

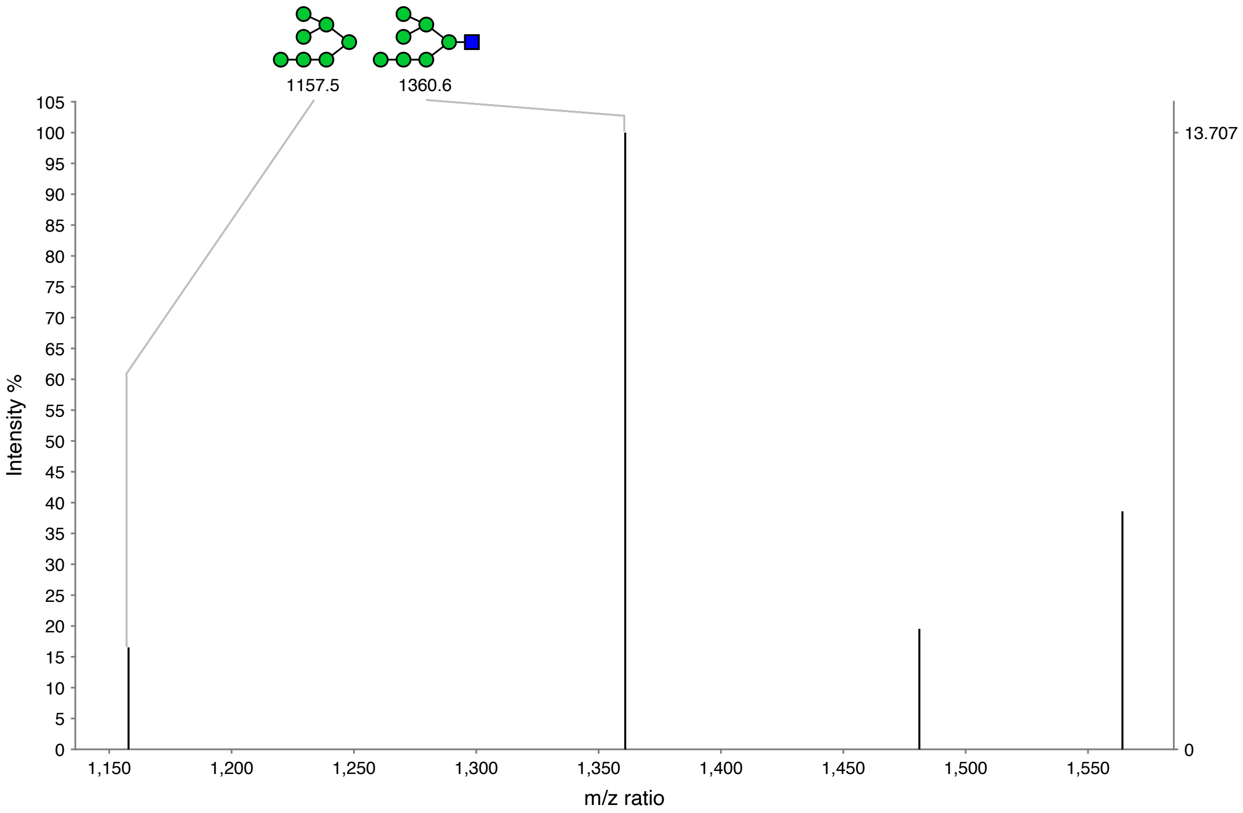

Supplemental Figure 13: Glycoworkbench generated annotation of 21T MALDI FTICR DDA fragmentation spectra of molecular ion m/z 1581.52821. Fragmentation spectra was annotated based on the most relevant glycoform as circled in red in Supplemental Table 17, which was identified from our data and additional literature review.

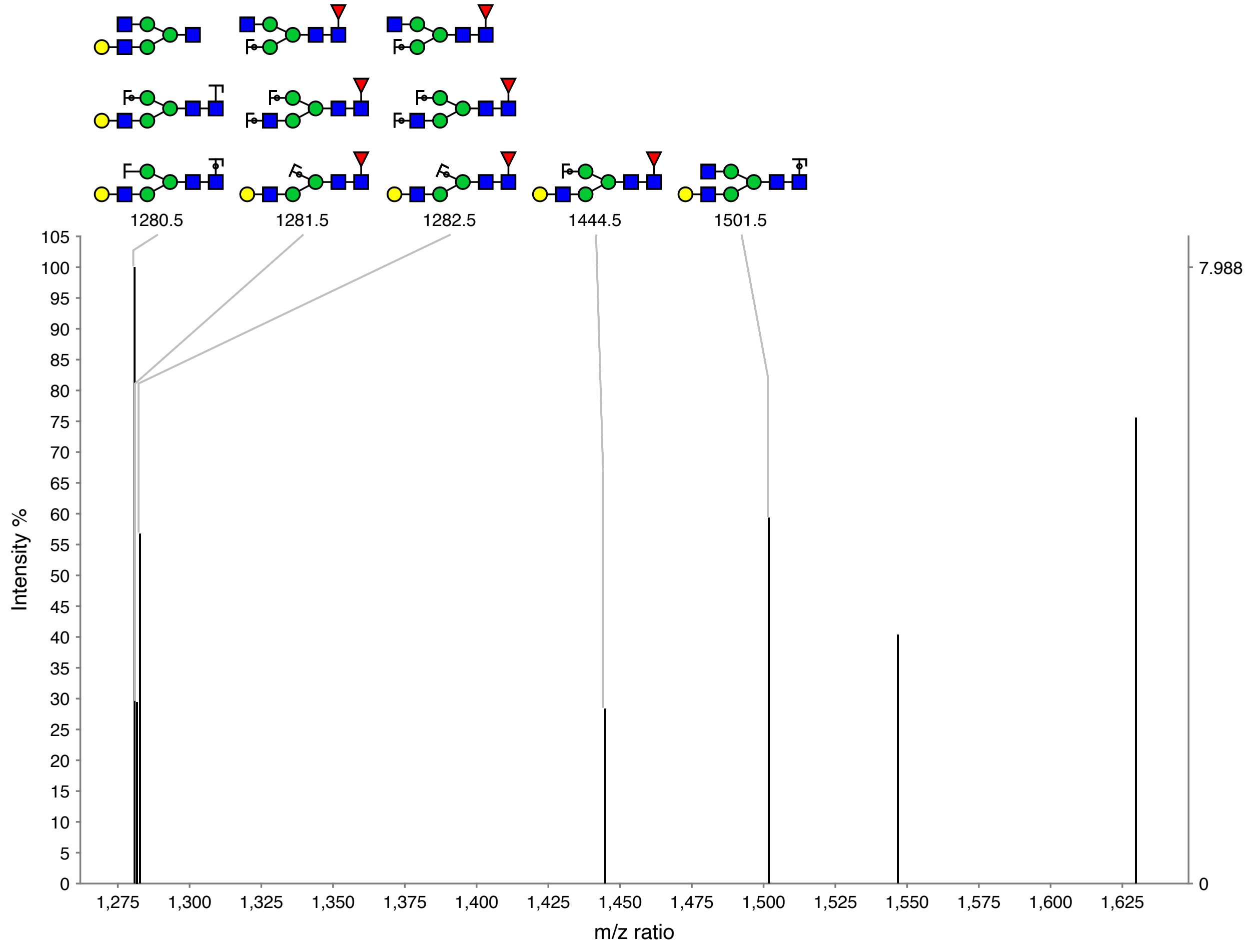

Supplemental Figure 14: Glycoworkbench generated annotation of 21T MALDI FTICR DDA fragmentation spectra of molecular ion m/z 1647.58700. Fragmentation spectra was annotated based on the most relevant glycoform as circled in red in Supplemental Table 18, which was identified from our data and additional literature review.

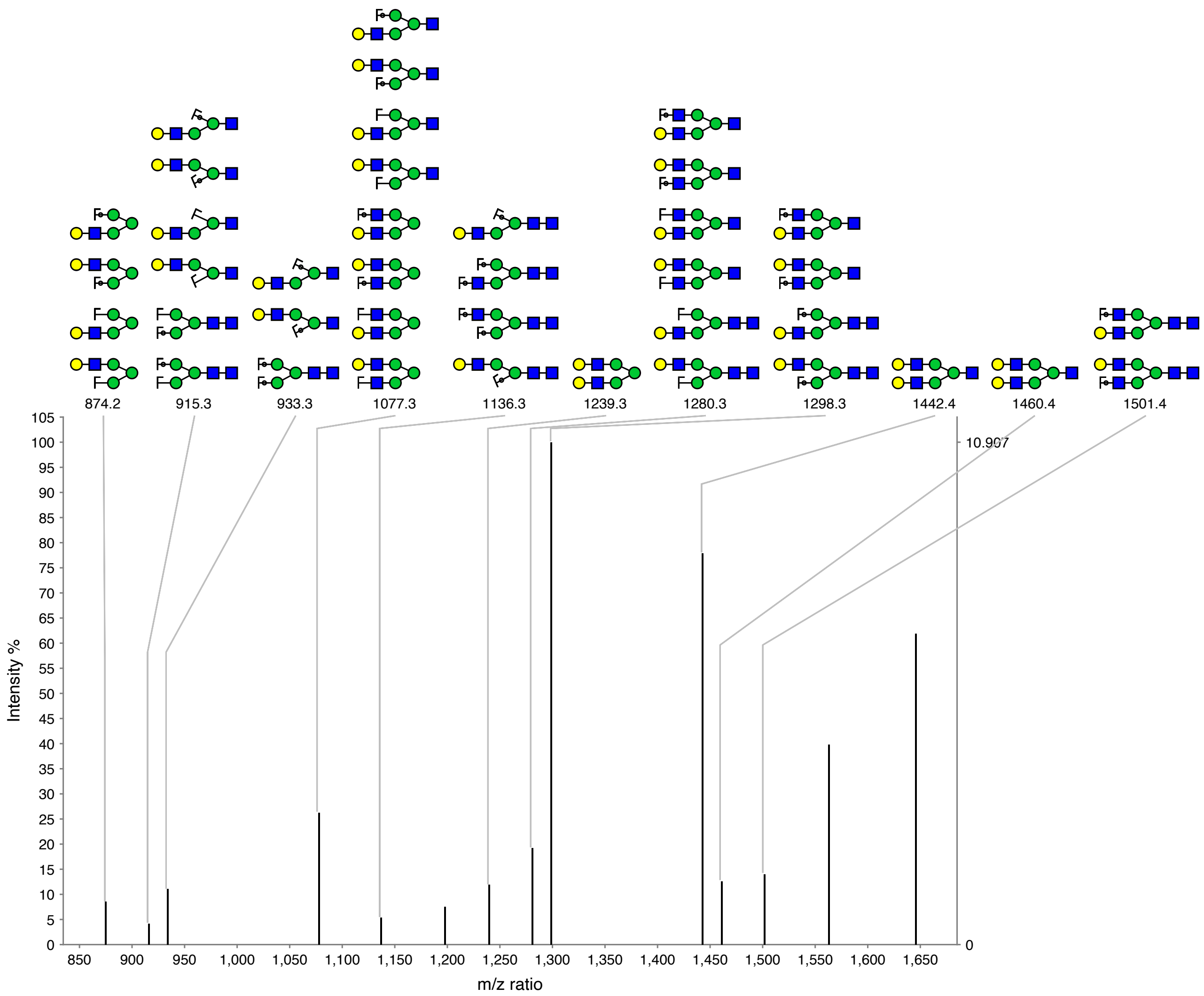

Supplemental Figure 15: Glycoworkbench generated annotation of 21T MALDI FTICR DDA fragmentation spectra of molecular ion m/z 1663.58113. Fragmentation spectra was annotated based on the most relevant glycoform as circled in red in Supplemental Table 19, which was identified from our data and additional literature review.

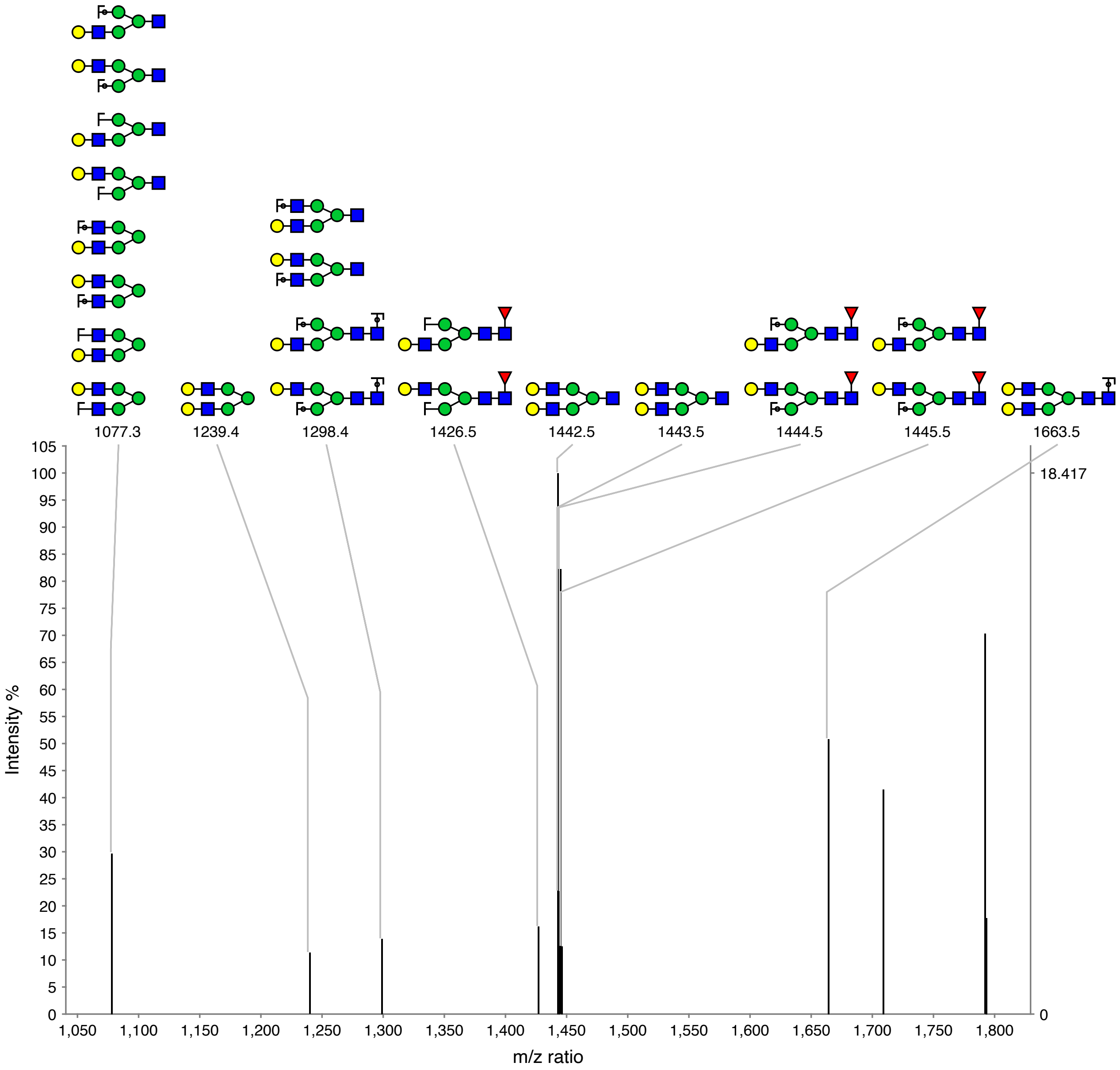

Supplemental Figure 16: Glycoworkbench generated annotation of 21T MALDI FTICR DDA fragmentation spectra of molecular ion m/z 1809.63931. Fragmentation spectra was annotated based on the most relevant glycoform as circled in red in Supplemental Table 20, which was identified from our data and additional literature review.

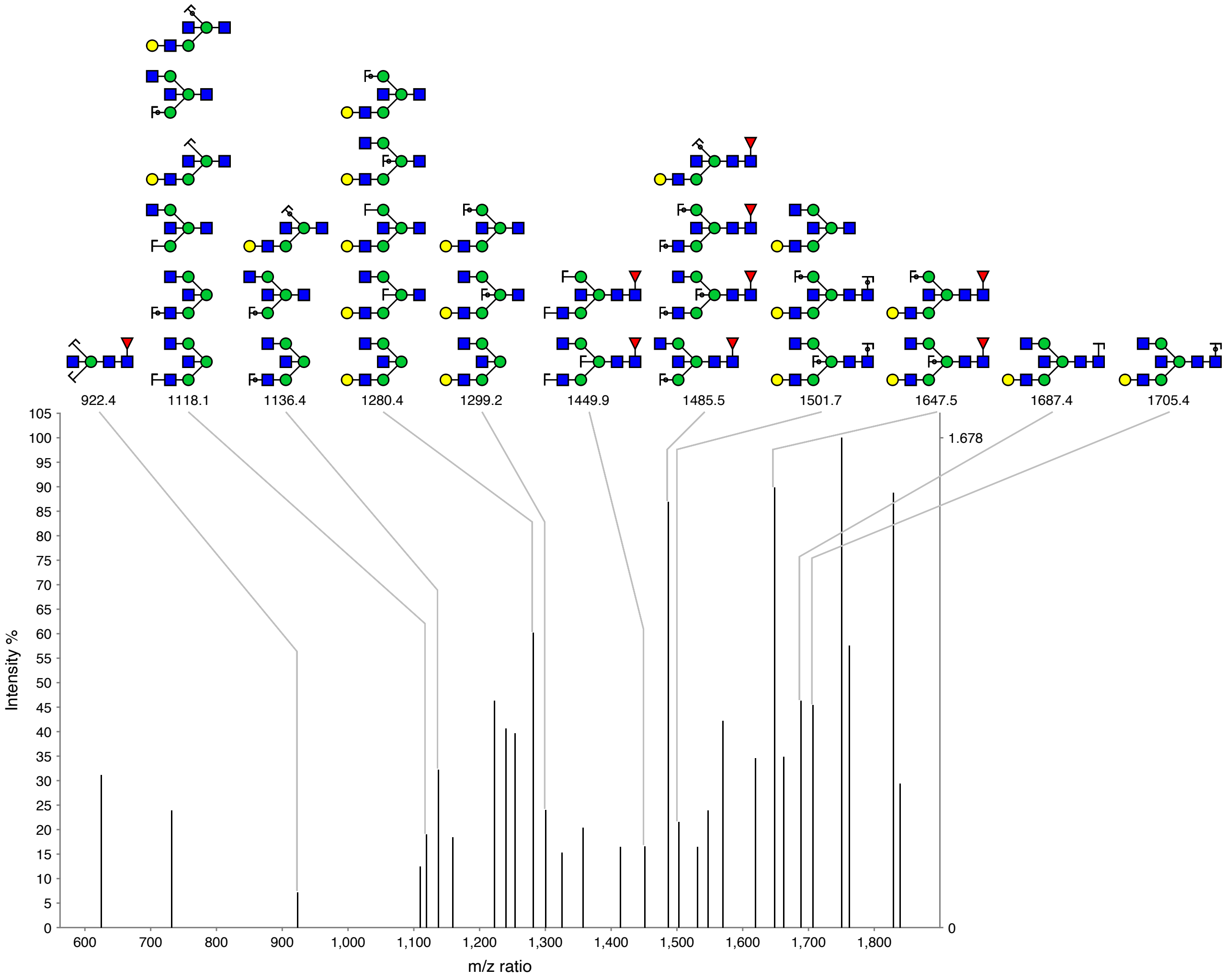

Supplemental Figure 17: Glycoworkbench generated annotation of 21T MALDI FTICR DDA fragmentation spectra of molecular ion m/z 1850.66465. Fragmentation spectra was annotated based on the most relevant glycoform as circled in red in Supplemental Table 21, which was identified from our data and additional literature review.

Supplemental Tables

Supplemental Table 1

Table 1: Frequency overview of tissue and annotation types present in the patient cohort

	Apoptotic Bodies	Cancer	Cancer mixed with Cancer-associated stroma	Cancer mixed with gliosis	Cancer mixed with lymphoplasmacytic infiltrate	Cancer mixed with meninges	Cancer mixed with stroma	DCIS mixed with lymphoplasmacytic infiltrate	Cancer-associated stroma	Necrosis	Normal	Stroma	Stroma (gliosis) adjacent to cancer	Stroma adjacent to cancer	Tumor infiltrating lymphocytes	Tumor stroma	Total TMAs for tissue type		Legend
Abdominal Lymph Node	2	3	3	0	0	0	0	0	2	2	3	1	0	1	0	0	**3**	Number of annotations	14
Adrenal Metastasis	0	5	6	0	0	0	0	0	3	2	5	1	0	1	0	0	**7**		
Adrenal Normal	0	1	1	0	0	0	0	0	1	0	10	1	0	0	0	0	**10**		
ANOTHER PATIENT Breast Primary	0	2	4	0	1	0	0	0	2	1	3	2	0	1	0	0	**4**		
Axillary Lymph Node	1	4	2	0	1	0	0	0	2	1	4	1	0	1	0	0	**4**		
Bone Metastasis	1	4	4	0	0	0	0	0	1	1	5	1	0	1	0	0	**5**		
Brain Metastasis	1	3	1	2	0	1	0	0	0	2	3	0	1	0	0	0	**3**		0
Breast Normal	0	2	1	0	1	0	0	0	1	1	14	11	0	0	0	0	**15**		
Breast Primary	1	12	13	0	6	0	0	0	10	6	10	4	0	3	1	0	**16**		
Diaphragm Metastasis	0	4	5	0	0	0	0	0	4	3	1	1	0	0	0	1	**5**		
Initial Lymph Node Metastasis	1	3	2	0	2	0	1	0	0	1	3	0	0	1	0	0	**3**		
Kidney Normal	0	0	0	0	0	0	0	0	0	0	5	1	0	0	0	0	**5**		
Liver Metastasis	1	9	12	0	0	0	0	0	8	6	13	3	0	2	0	0	**13**		
Liver Normal	0	0	1	0	0	0	0	0	0	0	8	0	0	0	0	0	**8**		
Lung Metastasis	4	10	9	0	0	0	0	0	8	6	11	2	0	2	0	0	**11**		
Lung Normal	0	2	1	0	0	0	0	0	1	2	3	0	0	0	0	0	**3**		
Mediastinal Lymph Node	2	4	4	0	0	0	0	0	3	4	2	0	0	1	0	0	**4**		
Omentum Metastasis	1	2	3	0	0	0	0	0	1	3	2	1	0	1	0	0	**3**		
Ovary Normal	0	0	0	0	0	0	0	0	0	0	4	1	0	0	0	0	**4**		
Pericardium Metastasis	0	2	2	0	0	0	0	0	1	1	0	0	0	1	0	0	**3**		
Pleura Metastasis	0	2	4	0	0	0	0	0	2	1	4	0	0	0	0	0	**4**		
Rib Metastasis	1	3	3	0	0	0	0	0	1	1	2	0	0	0	0	0	**3**		
Spleen Normal	0	0	0	0	0	0	0	0	0	0	4	0	0	0	0	0	**4**		
Vertebra Metastasis	1	2	3	0	1	0	0	0	2	0	3	0	0	0	0	0	**3**		

Frequency table reduced to annotation types present in most tissue types

	Apoptotic Bodies	Cancer	Cancer mixed with Cancer-associated stroma	Cancer-associated stroma	Necrosis	Normal	Stroma	Stroma adjacent to cancer	Total TMAs for tissue type
Abdominal Lymph Node	2	3	3	2	2	3	1	1	**3**
Adrenal Metastasis	0	5	6	3	2	5	1	1	**7**
Adrenal Normal	0	1	1	1	0	10	1	0	**10**
ANOTHER PATIENT Breast Primary	0	2	4	2	1	3	2	1	**4**
Axillary Lymph Node	1	4	2	2	1	4	1	1	**4**
Bone Metastasis	1	4	4	1	1	5	1	1	**5**
Brain Metastasis	1	3	1	0	2	3	0	0	**3**
Breast Normal	0	2	1	1	1	14	11	0	**15**
Breast Primary	1	12	13	10	6	10	4	3	**16**
Diaphragm Metastasis	0	4	5	4	3	1	1	0	**5**
Initial Lymph Node Metastasis	1	3	2	0	1	3	0	1	**3**
Kidney Normal	0	0	0	0	0	5	1	0	**5**
Liver Metastasis	1	9	12	8	6	13	3	2	**13**
Liver Normal	0	0	1	0	0	8	0	0	**8**
Lung Metastasis	4	10	9	8	6	11	2	2	**11**
Lung Normal	0	2	1	1	2	3	0	0	**3**
Mediastinal Lymph Node	2	4	4	3	4	2	0	1	**4**
Omentum Metastasis	1	2	3	1	3	2	1	1	**3**
Ovary Normal	0	0	0	0	0	4	1	0	**4**
Pericardium Metastasis	0	2	2	1	1	0	0	1	**3**
Pleura Metastasis	0	2	4	2	1	4	0	0	**4**
Rib Metastasis	1	3	3	1	1	2	0	0	**3**
Spleen Normal	0	0	0	0	0	4	0	0	**4**
Vertebra Metastasis	1	2	3	2	0	3	0	0	**3**

Supplemental Table 2

		Supplementary Table 2: Pairwise t-test complete list of results				
Structure	Name	Experimental mass Rapiflex (+/- 0.2 Da)	p-value	Class 1	Class 2	Class 1 increased expression
	Not identified	885.5	0.048	Breast Normal_Normal	Breast Primary_Cancer mixed with cancer-associated stroma	FALSE
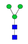	Hex3HexNAc2	933.4	0.046	Bone Metastasis_Cancer mixed with cancer-associated stroma	Liver Metastasis_Cancer mixed with cancer-associated stroma	FALSE
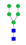	Hex5HexNAc2	1257.5	0.025	Bone Metastasis_Cancer	Liver Metastasis_Cancer mixed with cancer-associated stroma	FALSE
		1257.5	0.008	Bone Metastasis_Cancer mixed with cancer-associated stroma	Liver Metastasis_Cancer mixed with cancer-associated stroma	FALSE
		1257.5	0.019	Breast Normal_Normal	Lung Metastasis_Cancer	FALSE
		1257.5	0.012	Breast Normal_Normal	Lung Metastasis_Cancer mixed with cancer-associated stroma	FALSE
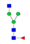	Hex3dHex1HexNAc3	1282.6	0.043	Bone Metastasis_Cancer mixed with cancer-associated stroma	Liver Metastasis_Cancer mixed with cancer-associated stroma	FALSE
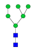	Hex6HexNAc2	1419.5	0.044	Bone Metastasis_Cancer	Liver Metastasis_Cancer mixed with cancer-associated stroma	FALSE
		1419.5	0.015	Bone Metastasis_Cancer mixed with cancer-associated stroma	Liver Metastasis_Cancer mixed with cancer-associated stroma	FALSE
		1419.5	0.008	Breast Normal_Normal	Breast Primary_Cancer mixed with cancer-associated stroma	FALSE
		1419.5	0.021	Breast Normal_Normal	Liver Metastasis_Cancer	FALSE
		1419.5	0.036	Breast Normal_Normal	Liver Metastasis_Cancer mixed with cancer-associated stroma	FALSE
		1419.5	0.012	Breast Normal_Normal	Lung Metastasis_Cancer	FALSE
		1419.5	0.012	Breast Normal_Normal	Lung Metastasis_Cancer mixed with cancer-associated stroma	FALSE
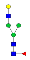	Hex4dHex1HexNAc3	1444.6	0.012	Bone Metastasis_Cancer mixed with cancer-associated stroma	Liver Metastasis_Cancer	TRUE
		1444.6	0.023	Bone Metastasis_Cancer mixed with cancer-associated stroma	Liver Metastasis_Cancer mixed with cancer-associated stroma	TRUE
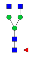	Hex3dHex1HexNAc4	1485.6	0.021	Breast Normal_Normal	Liver Metastasis_Cancer	FALSE
		1485.6	0.036	Breast Normal_Normal	Liver Metastasis_Cancer mixed with cancer-associated stroma	FALSE
		1485.6	0.023	Breast Normal_Normal	Lung Metastasis_Cancer	FALSE
		1485.6	0.026	Breast Normal_Normal	Lung Metastasis_Cancer mixed with cancer-associated stroma	FALSE
		1485.6	0.042	Breast Primary_Cancer	Lung Metastasis_Cancer	FALSE
		1485.6	0.018	Breast Primary_Cancer	Lung Metastasis_Cancer mixed with cancer-associated stroma	FALSE
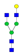	Hex4HexNAc4	1501.6	0.002	Bone Metastasis_Cancer mixed with cancer-associated stroma	Liver Metastasis_Cancer	TRUE
		1501.6	0.008	Bone Metastasis_Cancer mixed with cancer-associated stroma	Liver Metastasis_Cancer mixed with cancer-associated stroma	TRUE
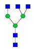	Hex3HexNAc5	1542.7	0.049	Bone Metastasis_Cancer mixed with cancer-associated stroma	Breast Normal_Normal	FALSE
		1542.7	0.042	Breast Primary_Cancer	Lung Metastasis_Cancer	FALSE
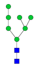	Hex7HexNAc2	1581.6	0.048	Bone Metastasis_Cancer	Liver Metastasis_Cancer mixed with cancer-associated stroma	FALSE
		1581.6	0.043	Bone Metastasis_Cancer mixed with cancer-associated stroma	Liver Metastasis_Cancer mixed with cancer-associated stroma	FALSE
		1581.6	0.015	Breast Normal_Normal	Breast Primary_Cancer mixed with cancer-associated stroma	FALSE
		1581.6	0.021	Breast Normal_Normal	Liver Metastasis_Cancer	FALSE
		1581.6	0.036	Breast Normal_Normal	Liver Metastasis_Cancer mixed with cancer-associated stroma	FALSE
		1581.6	0.014	Breast Normal_Normal	Lung Metastasis_Cancer	FALSE
		1581.6	0.012	Breast Normal_Normal	Lung Metastasis_Cancer mixed with cancer-associated stroma	FALSE
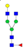	Hex4dHex1HexNAc4	1647.7	0.049	Bone Metastasis_Cancer mixed with cancer-associated stroma	Liver Metastasis_Cancer mixed with cancer-associated stroma	TRUE
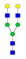	Hex5HexNAc4	1663.7	0.040	Bone Metastasis_Cancer mixed with cancer-associated stroma	Breast Normal_Normal	TRUE
		1663.7	0.019	Bone Metastasis_Cancer mixed with cancer-associated stroma	Breast Primary_Cancer mixed with cancer-associated stroma	TRUE
		1663.7	0.019	Bone Metastasis_Cancer mixed with cancer-associated stroma	Liver Metastasis_Cancer mixed with cancer-associated stroma	TRUE
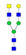	Hex4HexNAc5	1704.8	0.043	Breast Normal_Normal	Liver Metastasis_Cancer mixed with cancer-associated stroma	TRUE
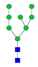	Hex8HexNAc2	1743.7	0.039	Bone Metastasis_Cancer mixed with cancer-associated stroma	Liver Metastasis_Cancer mixed with cancer-associated stroma	FALSE
		1743.7	0.021	Breast Normal_Normal	Breast Primary_Cancer mixed with cancer-associated stroma	FALSE
		1743.7	0.038	Breast Normal_Normal	Liver Metastasis_Cancer	FALSE
		1743.7	0.049	Breast Normal_Normal	Liver Metastasis_Cancer mixed with cancer-associated stroma	FALSE
		1743.7	0.012	Breast Normal_Normal	Lung Metastasis_Cancer	FALSE
		1743.7	0.012	Breast Normal_Normal	Lung Metastasis_Cancer mixed with cancer-associated stroma	FALSE
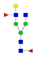	Hex4dHex2HexNAc4	1793.7	0.049	Bone Metastasis_Cancer mixed with cancer-associated stroma	Breast Normal_Normal	TRUE
		1793.7	0.014	Bone Metastasis_Cancer mixed with cancer-associated stroma	Liver Metastasis_Cancer	TRUE
		1793.7	0.008	Bone Metastasis_Cancer mixed with cancer-associated stroma	Liver Metastasis_Cancer mixed with cancer-associated stroma	TRUE
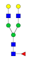	Hex5dHex1HexNAc4	1809.8	0.044	Bone Metastasis_Cancer	Liver Metastasis_Cancer mixed with cancer-associated stroma	TRUE
		1809.8	0.049	Bone Metastasis_Cancer mixed with cancer-associated stroma	Breast Normal_Normal	TRUE
		1809.8	0.001	Bone Metastasis_Cancer mixed with cancer-associated stroma	Liver Metastasis_Cancer	TRUE
		1809.8	0.008	Bone Metastasis_Cancer mixed with cancer-associated stroma	Liver Metastasis_Cancer mixed with cancer-associated stroma	TRUE
		1809.8	0.008	Breast Normal_Normal	Breast Primary_Cancer mixed with cancer-associated stroma	FALSE
	Not identified	1838.8	0.015	Bone Metastasis_Cancer mixed with cancer-associated stroma	Liver Metastasis_Cancer mixed with cancer-associated stroma	TRUE
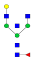	Hex4dHex1HexNAc5	1850.8	0.028	Breast Normal_Normal	Breast Primary_Cancer	TRUE
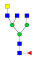	Hex3dHex1HexNAc6	1891.8	0.049	Bone Metastasis_Cancer mixed with cancer-associated stroma	Breast Normal_Normal	TRUE
		1891.8	0.046	Bone Metastasis_Cancer mixed with cancer-associated stroma	Liver Metastasis_Cancer mixed with cancer-associated stroma	TRUE
		1891.8	0.022	Breast Normal_Normal	Lung Metastasis_Cancer mixed with cancer-associated stroma	FALSE
		1891.8	0.042	Breast Primary_Cancer	Lung Metastasis_Cancer	FALSE
		1891.8	0.006	Breast Primary_Cancer	Lung Metastasis_Cancer mixed with cancer-associated stroma	FALSE
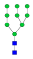	Hex9HexNAc2	1905.8	0.046	Bone Metastasis_Cancer mixed with cancer-associated stroma	Liver Metastasis_Cancer mixed with cancer-associated stroma	FALSE
		1905.8	0.046	Breast Normal_Normal	Breast Primary_Cancer mixed with cancer-associated stroma	FALSE
		1905.8	0.023	Breast Normal_Normal	Lung Metastasis_Cancer	FALSE
		1905.8	0.015	Breast Normal_Normal	Lung Metastasis_Cancer mixed with cancer-associated stroma	FALSE
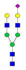	Hex5HexNAc4NeuAc1 + 2Na	1976.8	0.023	Breast Normal_Normal	Lung Metastasis_Cancer	FALSE
		1976.8	0.012	Breast Normal_Normal	Lung Metastasis_Cancer mixed with cancer-associated stroma	FALSE
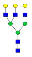	Hex6HexNAc5	2028.8	0.023	Breast Normal_Normal	Lung Metastasis_Cancer	FALSE
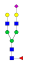	Hex5dHex1HexNAc4NeuAc1 + 2Na	2122.9	0.040	Breast Normal_Normal	Breast Primary_Cancer mixed with cancer-associated stroma	FALSE
	Not identified	2150.8	0.023	Breast Normal_Normal	Lung Metastasis_Cancer	FALSE
		2150.8	0.015	Breast Normal_Normal	Lung Metastasis_Cancer mixed with cancer-associated stroma	FALSE
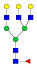	Hex6dHex1HexNAc5	2174.9	0.049	Bone Metastasis_Cancer mixed with cancer-associated stroma	Breast Normal_Normal	TRUE
		2174.9	0.014	Bone Metastasis_Cancer mixed with cancer-associated stroma	Breast Primary_Cancer	TRUE
		2174.9	0.029	Bone Metastasis_Cancer mixed with cancer-associated stroma	Liver Metastasis_Cancer mixed with cancer-associated stroma	TRUE
		2174.9	0.046	Breast Normal_Normal	Breast Primary_Cancer mixed with cancer-associated stroma	FALSE
		2174.9	0.046	Breast Normal_Normal	Lung Metastasis_Cancer mixed with cancer-associated stroma	FALSE
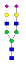	Hex5HexNAc4NeuAc2 + 3Na	2289.9	0.048	Breast Normal_Normal	Liver Metastasis_Cancer	FALSE
		2289.9	0.014	Breast Normal_Normal	Lung Metastasis_Cancer	FALSE
		2289.9	0.012	Breast Normal_Normal	Lung Metastasis_Cancer mixed with cancer-associated stroma	FALSE
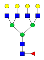	Hex7dHex1HexNAc6	2540.1	0.049	Bone Metastasis_Cancer mixed with cancer-associated stroma	Liver Metastasis_Cancer mixed with cancer-associated stroma	TRUE

Supplemental Table 3

Supplementary Table 3: Significant N-glycans in pairwise comparisons between bone and liver metastases

m/z	p-value	class 1	class 2
933.4	0.046	Bone Metastasis_Cancer mixed with cancer-associated stroma	Liver Metastasis_Cancer mixed with cancer-associated stroma
1257.5	0.008	Bone Metastasis_Cancer mixed with cancer-associated stroma	Liver Metastasis_Cancer mixed with cancer-associated stroma
1282.5	0.043	Bone Metastasis_Cancer mixed with cancer-associated stroma	Liver Metastasis_Cancer mixed with cancer-associated stroma
1419.6	0.015	Bone Metastasis_Cancer mixed with cancer-associated stroma	Liver Metastasis_Cancer mixed with cancer-associated stroma
1444.6	0.023	Bone Metastasis_Cancer mixed with cancer-associated stroma	Liver Metastasis_Cancer mixed with cancer-associated stroma
1501.6	0.008	Bone Metastasis_Cancer mixed with cancer-associated stroma	Liver Metastasis_Cancer mixed with cancer-associated stroma
1581.6	0.043	Bone Metastasis_Cancer mixed with cancer-associated stroma	Liver Metastasis_Cancer mixed with cancer-associated stroma
1647.7	0.049	Bone Metastasis_Cancer mixed with cancer-associated stroma	Liver Metastasis_Cancer mixed with cancer-associated stroma
1663.7	0.019	Bone Metastasis_Cancer mixed with cancer-associated stroma	Liver Metastasis_Cancer mixed with cancer-associated stroma
1743.6	0.039	Bone Metastasis_Cancer mixed with cancer-associated stroma	Liver Metastasis_Cancer mixed with cancer-associated stroma
1793.7	0.008	Bone Metastasis_Cancer mixed with cancer-associated stroma	Liver Metastasis_Cancer mixed with cancer-associated stroma
1809.7	0.008	Bone Metastasis_Cancer mixed with cancer-associated stroma	Liver Metastasis_Cancer mixed with cancer-associated stroma
1838.7	0.015	Bone Metastasis_Cancer mixed with cancer-associated stroma	Liver Metastasis_Cancer mixed with cancer-associated stroma
1891.8	0.046	Bone Metastasis_Cancer mixed with cancer-associated stroma	Liver Metastasis_Cancer mixed with cancer-associated stroma
1905.7	0.046	Bone Metastasis_Cancer mixed with cancer-associated stroma	Liver Metastasis_Cancer mixed with cancer-associated stroma
2174.9	0.029	Bone Metastasis_Cancer mixed with cancer-associated stroma	Liver Metastasis_Cancer mixed with cancer-associated stroma
2540.1	0.049	Bone Metastasis_Cancer mixed with cancer-associated stroma	Liver Metastasis_Cancer mixed with cancer-associated stroma

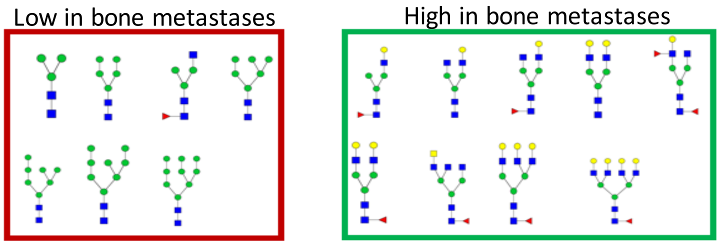

Supplemental Table 4

Supplementary Table 4: Surgical and therapeutic interventions of patients in cohort

Case number	1	2	3	4	5	6	7	8	9	10	11	12	13	14	15	16	17
**Surgical intervention**	prophylactic right and radical left mastectomy	lumpectomy	radical mastectomy with axillary ln dissection	partial mastectomy with axillary ln dissection	radical mastectomy	mastectomy, radiosurgery - gamma knife theraphy	lumpectomy	mastectomy	lumpectomy and axillary dissection	total mastectomies with left sentinel and axillary lumphadenectomy	N/A	N/A	bilateral mastectomy	lumpectomy and mastectomy	left mastectomy with axillary dissection, gamma knife radiosurgery	radical mastectomy	bilateral mastectomy
**Therapeutic intervention**	cyclophosphamide<br>methotrexate<br>fluorouracil	chemotherapy<br>radiotheraphy<br>chemotheraphy<br>oxygen<br>palliative radiotheraphy	adriamycin<br>cytoxan<br>tamoxifen<br>navelbine<br>zoladex<br>zolmetra<br>taxol<br>herceptin<br>radiation	taxotere<br>tamoxifen<br>radiotheraphy<br>arimidex<br>pamidronate<br>faslodex<br>zometa<br>doxil<br>radiation<br>gemzar	adriamycin<br>cytoxan<br>arimidex<br>tamoxifen<br>taxotere<br>xeloda<br>radiation<br>celecoxib<br>mirtazapine<br>oxycontin<br>phenytek<br>cetirizine<br>paroxetine<br>pantoprazole<br>verapamil<br>oxybutynin<br>albuterol inhaler<br>oxycodone	paclitaxel<br>carboplatin<br>docetaxel<br>tamoxifen<br>radiotherapy<br>tazobactam<br>bicarbonate<br>piperacillin	tamoxifen<br>radiation<br>arimidex<br>vaccine trial<br>faslodex<br>doxil<br>synthroid<br>oxycodone	tamofixen<br>lumpectomy<br>adriamycin<br>taxotere<br>radiation<br>zoladex<br>arimidex<br>tamoxifen<br>zometa<br>radiation<br>xeloda<br>taxotere	second lumpectomy<br>radiation<br>chemotheraphy	taxol<br>herceptin<br>declomycin<br>zosyn<br>linezolid<br>ciprofloxacin<br>flagyl<br>clindamycin	N/A	taxol<br>cyclophosphamide<br>methotrexate<br>fluorouracil<br>radiation	chemotheraphy<br>adriamycin<br>cytoxan<br>taxol<br>tamoxifen<br>radiation	anzemet<br>decadron<br>adriamycin<br>cytoxan<br>neulasta<br>taxol<br>avastin<br>navelbine<br>xeloda<br>oxycodone<br>fentanyl<br>lexapro<br>verapamil	taxotere<br>adriamycin<br>cytoxan<br>radiation<br>herceptin<br>xeloda<br>pertuzumab<br>trastuzumab<br>lapatinib<br>herceptin<br>tykerb<br>xeloda<br>doxil	adriamycin<br>cytoxan<br>cyclophosphamide<br>thiotepa<br>tamoxifen<br>femara<br>gemcitabine<br>taxol<br>avastin<br>radiation<br>oxycontin<br>oxycodone	tamoxifen<br>xeloda<br>paclitaxel<br>navelbine<br>paxil<br>taxol<br>avastin

Supplemental Table 5

Supplementary Table 5: Correlation analysis between levels of N-glycans and EpCAM in PT versus metastases

m/z value	Corrected p-value
1485.6	0.003
1257.5	0.013
1976.8	0.013
1647.7	0.04
1891.8	0.04
2028.8	0.04
2150.8	0.04

Supplemental Table 6

Supplementary Table 6: Resulting 876 DE genes encoding N-glycosylated proteins from TCGA dataset

This list displays the overlap between differentially-expressed genes in the TCGA breast cancer dataset and known N-glycosylated proteins from dbPTM.

gene	adj.P.Val
IBSP	7.21E-223
NTRK2	9.10E-192
MME	5.38E-154
SGCE	3.53E-138
TINAGL1	2.26E-137
MET	1.36E-133
SPARCL1	6.99E-127
PROS1	1.89E-124
SFRP1	2.34E-124
ADAMTS5	9.14E-119
SLC24A2	4.53E-112
PRNP	7.93E-107
JAM2	1.95E-106
EGFR	7.49E-106
PAMR1	1.64E-101
CHL1	6.23E-100
JAM3	1.07E-99
A2M	3.80E-96
LIFR	1.27E-94
PRIMA1	1.78E-93
CCL11	9.01E-93
ATP1B2	1.28E-91
SIRPA	4.03E-88
ABCA8	1.20E-87
FAM171A1	6.46E-85
FLRT2	3.78E-84
TF	1.41E-83
SMOC1	1.02E-82
OGN	4.78E-82
RELN	7.14E-82
LGR4	1.06E-78
FGFR1	2.51E-77
ABCA9	6.96E-77
CRYAB	8.12E-76
APOD	2.07E-75
SELP	7.25E-75
MMP13	1.60E-74
CD59	1.78E-74
CFI	2.48E-74
STEAP4	3.05E-74
PKD2	3.81E-72
NCAM1	4.27E-72
UBAP2L	8.90E-72
ABCA6	5.41E-71
PROC	7.17E-71
F3	1.54E-70
NLGN4X	1.62E-70
MMP1	7.45E-70
ATP6AP1	1.11E-69
ITGA6	1.80E-69
CLCN6	9.52E-69
CADM3	1.03E-68
LCAT	1.18E-68
DPP6	2.19E-68
COL6A6	1.39E-66
TFR2	1.66E-66
IL6R	6.66E-66
GLG1	8.91E-66
TFPI	2.29E-64
ITGA9	7.81E-64
SLC9A6	2.49E-63
LEPR	2.75E-63
MASP1	3.16E-63
HYAL1	5.05E-63
TRPC6	5.37E-62
CD36	8.61E-61
COL14A1	3.08E-59
H6PD	1.41E-58
TSPAN2	4.64E-58
MRAP2	5.23E-58
LMBRD1	5.32E-58
FAT2	2.13E-56
IGFBP6	1.54E-55
HM13	1.81E-55
BACE1	2.45E-55
MFAP4	2.51E-55
SULF1	9.12E-55
ABCG2	1.08E-54
FAT1	1.81E-54
DNAJB11	1.83E-54
S1PR1	1.92E-54
MAP1B	1.04E-53
C7	1.12E-53
TMEM117	1.47E-53
SAA4	1.57E-53
MMRN2	1.84E-53
EFNA5	4.54E-53
DCN	3.64E-52
SCPEP1	5.15E-52
EFNB2	6.75E-52
LYVE1	8.89E-52
F11R	1.49E-51
FCER1A	1.62E-51
CD34	1.63E-51
CALR	2.43E-51
IGF2	2.86E-51
CES2	2.90E-51
F10	4.64E-51
RPN1	4.79E-51
EPHA2	1.23E-50
MMRN1	1.34E-50

gene	adj.P.Val
GBA	1.91E-50
SOD3	2.06E-50
SLC2A4	2.53E-50
ABI3BP	3.31E-50
CES1	1.01E-49
ITM2B	1.13E-49
STAB2	1.44E-49
LAMA2	1.56E-49
BGN	2.92E-49
KCNE1	9.77E-49
GYPC	1.69E-48
IL21R	2.05E-48
F8	2.73E-48
LPL	4.41E-48
DNER	4.99E-48
CLEC3B	5.99E-48
PARM1	1.48E-47
MFGE8	1.74E-47
CA9	2.67E-47
ACE2	3.51E-47
NAALAD2	4.55E-47
FGF9	4.65E-47
TLR3	9.84E-47
CGA	1.18E-46
BMP2	3.62E-46
RPN2	7.66E-46
CDH13	1.15E-45
IL4I1	3.10E-45
SLC19A3	3.68E-45
COMP	3.90E-45
TSPAN8	1.12E-44
ACVR1	1.16E-44
P4HA1	1.39E-44
ITGA7	1.60E-44
MERTK	1.84E-44
FN1	1.86E-44
PRELP	3.84E-44
DNASE2	4.42E-44
TEK	5.02E-44
KIT	5.32E-44
ERGIC1	5.88E-44
AGTR1	6.17E-44
PTGDS	8.86E-44
FLT4	9.00E-44
ALPL	2.18E-43
CUBN	2.38E-43
LINGO1	3.70E-43
TSPAN13	4.18E-43
VIPR1	5.85E-43
ROBO4	6.65E-43
AOC3	1.15E-42
MYOC	1.41E-42
APOB	1.88E-42
CNTN4	2.39E-42
NEU1	2.43E-42
PTPRZ1	2.52E-42
CNTN3	4.40E-42
PIGT	9.04E-42
TLR4	3.09E-41
SERINC1	4.03E-41
FGL2	1.08E-40
CD80	1.48E-40
GRIA4	1.63E-40
SERPING1	1.98E-40
ICAM2	2.28E-40
CDH5	4.00E-40
PTGS2	4.29E-40
GPLD1	4.85E-40
EPDR1	4.87E-40
PIK3IP1	4.99E-40
DPEP1	6.41E-40
ERMP1	6.45E-40
TAPBP	6.70E-40
ADAMTSL4	1.01E-39
TNFSF4	1.89E-39
CNTN1	5.99E-39
DPP4	6.80E-39
CMA1	1.53E-38
PLAUR	2.57E-38
KDR	2.92E-38
IFNB1	2.95E-38
CD276	3.26E-38
CHST2	9.72E-38
FZD5	1.51E-37
VWF	1.88E-37
BCHE	2.28E-37
HBB	3.38E-37
DSG1	4.05E-37
ST3GAL6	5.44E-37
CHST7	6.53E-37
ACAN	1.92E-36
CLCA4	2.48E-36
SUN2	2.87E-36
PTPRM	3.26E-36
CLN3	3.71E-36
BSCL2	4.47E-36
SGCB	5.03E-36
ANPEP	5.96E-36
SLC13A5	7.45E-36
FOXRED2	8.60E-36
SLC9A9	1.60E-35

gene	adj.P.Val
ATP13A1	1.64E-35
GGH	1.86E-35
F12	2.32E-35
SIRPB1	2.32E-35
CHST11	5.54E-35
MCOLN3	6.94E-35
FBN2	8.24E-35
PZP	1.21E-34
YIPF3	1.40E-34
CLU	1.89E-34
SLC4A4	2.11E-34
ADAMTSL2	2.91E-34
STIM1	3.07E-34
ITPR1	3.30E-34
GLB1	5.06E-34
TFDP1	6.91E-34
ANO6	7.00E-34
LAMC1	7.91E-34
GAS6	8.67E-34
TMED9	1.09E-33
MOGS	1.31E-33
POSTN	1.99E-33
GRIN2A	2.00E-33
CLPTM1L	4.29E-33
FAS	6.53E-33
FKBP10	7.46E-33
TNFRSF21	7.95E-33
PNLIPRP2	8.27E-33
DNASE1	1.17E-32
TTYH3	2.06E-32
GRIN2B	2.57E-32
APLP1	3.67E-32
SSR2	4.86E-32
PLTP	6.84E-32
LRP6	7.62E-32
NCSTN	8.25E-32
TNN	9.96E-32
NFASC	2.65E-31
SEMA7A	3.41E-31
CFB	3.94E-31
VIM	4.43E-31
MMP27	5.34E-31
PECAM1	6.11E-31
CAPZA3	7.77E-31
C3	9.44E-31
CALHM1	1.22E-30
C4BPA	1.43E-30
LAMB2	2.02E-30
RHBG	2.10E-30
LRP1	2.26E-30
PCYOX1	2.79E-30
MCAM	2.92E-30
NOMO1	3.07E-30
MYC	4.70E-30
IGSF8	1.45E-29
FOXD3	1.46E-29
DLK1	2.90E-29
ABCC6	4.27E-29
LMAN2	5.36E-29
SLC19A1	5.98E-29
SIAE	6.41E-29
ELANE	6.96E-29
NUP210	1.43E-28
CEACAM5	4.17E-28
C1RL	4.21E-28
CD40	8.35E-28
CLDND1	1.54E-27
CTSD	1.64E-27
OXTR	1.64E-27
MPO	1.83E-27
OLFM1	1.95E-27
EFNA1	3.93E-27
RHBDF1	4.17E-27
GAL3ST1	4.49E-27
ITGB3	4.56E-27
IL6	7.27E-27
CLPTM1	7.79E-27
SPEN	8.61E-27
SYNPO	9.87E-27
ADAMDEC1	1.44E-26
LRIG1	1.84E-26
RET	1.88E-26
MRC1	2.02E-26
CD163L1	2.48E-26
HLA-E	2.90E-26
MAN2A2	3.20E-26
PTPRG	4.00E-26
FAP	4.29E-26
PMP22	5.64E-26
SLC23A1	5.93E-26
OLR1	6.76E-26
NT5E	8.04E-26
STS	1.05E-25
KCNA2	1.17E-25
TYRP1	1.42E-25
IL12B	1.63E-25
NRP1	1.65E-25
GOLM1	1.84E-25
RNF13	1.84E-25
CD1C	1.92E-25
ESAM	2.60E-25

gene	adj.P.Val
FAM3D	9.70E-25
ITIH3	1.05E-24
EMILIN2	1.21E-24
FSTL1	1.41E-24
IFNAR1	1.42E-24
ORM1	1.51E-24
SLC3A2	1.77E-24
CTSF	2.35E-24
SLC2A1	2.85E-24
VEGFC	3.27E-24
APOM	3.69E-24
ERLIN1	3.73E-24
ECE1	4.35E-24
SULF2	5.02E-24
MARCO	5.31E-24
SPP1	6.05E-24
SLC14A2	8.77E-24
HSD11B1	9.34E-24
GABRB3	9.57E-24
LILRB5	1.11E-23
ERAP1	1.19E-23
CNPY3	1.20E-23
CFH	1.28E-23
TFRC	1.36E-23
HEG1	1.87E-23
CLN5	2.90E-23
ITGA1	3.04E-23
NAGA	3.17E-23
C1R	3.47E-23
F2	4.81E-23
MAL2	5.49E-23
EPO	7.33E-23
FCGR1A	1.17E-22
RAET1G	1.29E-22
ANGPTL6	1.40E-22
BST1	1.49E-22
CDKN2AIP	1.56E-22
COL12A1	2.58E-22
C6	2.72E-22
ASPH	3.09E-22
IFNAR2	5.28E-22
PPIB	6.04E-22
HYOU1	6.81E-22
COL17A1	7.44E-22
HLA-C	7.45E-22
NRG1	8.14E-22
ACP2	1.24E-21
A1BG	1.25E-21
SPON1	1.38E-21
PPT2	1.53E-21
PPT1	1.69E-21
PI15	1.78E-21
IL1RAP	1.83E-21
HBEGF	2.32E-21
TIMP1	2.66E-21
AADAC	3.09E-21
HCFC1	4.22E-21
GANAB	4.37E-21
ABCA1	6.94E-21
RTN4R	7.84E-21
KCNH6	7.99E-21
CA11	8.68E-21
CEACAM6	9.24E-21
IGFBP3	1.06E-20
CBLN1	1.09E-20
PROZ	1.39E-20
MST1R	1.92E-20
C6orf120	2.04E-20
IGFALS	2.30E-20
BTN3A1	2.57E-20
GDF15	2.76E-20
FOLR2	3.05E-20
PIGR	3.12E-20
HPN	3.12E-20
GALNS	3.13E-20
FIP1L1	3.17E-20
CDH2	3.54E-20
SERPINF1	3.95E-20
ITIH2	4.37E-20
C1S	4.51E-20
SERPINH1	8.05E-20
TGFB1	8.37E-20
TSHR	8.90E-20
PLBD1	1.13E-19
ULBP2	1.34E-19
ENTPD6	1.45E-19
LTA	1.58E-19
PTTG1IP	1.61E-19
LAMA4	1.86E-19
ITGB1	1.91E-19
BSG	2.04E-19
GFRA3	3.01E-19
C5	3.05E-19
CPM	3.06E-19
OIT3	3.31E-19
PROCR	3.63E-19
PTPRS	4.56E-19
LY75	4.90E-19
KLK5	8.32E-19
CDH6	8.53E-19
MUC1	9.49E-19

gene	adj.P.Val
P2RX4	1.29E-18
CSF3	1.43E-18
NTNG2	1.57E-18
NPC1	2.23E-18
FOLR1	2.24E-18
CREG1	3.80E-18
ITFG1	2.82E-18
SYN1	3.24E-18
SLC29A1	3.65E-18
HSP90B1	4.17E-18
IDS	6.47E-18
PRG2	6.51E-18
NFRKB	7.23E-18
BST2	9.10E-18
AMY1A	9.58E-18
TYRO3	1.06E-17
TPST1	1.21E-17
SERPINF2	1.46E-17
RNASET2	1.73E-17
PLAT	2.62E-17
BTN3A3	2.82E-17
TOR1A	3.02E-17
PODN	3.42E-17
HTR1F	4.11E-17
TOR1AIP1	4.60E-17
TSPAN1	5.30E-17
CNTN2	5.35E-17
CRB3	7.42E-17
CD1A	9.88E-17
DCBLD1	1.20E-16
FCGR3A	1.33E-16
ANO7	1.66E-16
CXCR4	2.10E-16
FBLN1	2.40E-16
L1CAM	3.22E-16
GMEB2	4.36E-16
AFP	5.02E-16
CD300LF	5.09E-16
KLK6	5.25E-16
CA6	5.31E-16
CALU	6.75E-16
TXNDC15	7.20E-16
MAN2B1	7.41E-16
AEBP1	7.48E-16
PON3	8.69E-16
EPHB2	1.03E-15
IL17RA	1.03E-15
RNF130	1.53E-15
SLC1A4	1.55E-15
CD47	1.68E-15
CCR4	1.68E-15
TMED4	1.70E-15
SCTR	1.75E-15
CREB3L3	2.25E-15
GLP1R	2.26E-15
GRN	2.48E-15
SMPDL3A	2.68E-15
ADAMTSL1	2.75E-15
PTPRN	2.80E-15
ENPEP	2.87E-15
LAMA1	3.00E-15
ERGIC3	5.96E-15
GAA	7.13E-15
PRB3	8.40E-15
NOG	1.04E-14
CD244	1.15E-14
CP	1.16E-14
TPBG	1.40E-14
ASPN	1.44E-14
ST3GAL1	1.58E-14
GPNMB	1.60E-14
TLR1	1.79E-14
FCN2	1.95E-14
WNT2B	2.11E-14
ICAM5	2.13E-14
ITGAX	2.45E-14
SLC46A1	2.51E-14
KCNK1	2.54E-14
PRF1	2.69E-14
KNG1	2.85E-14
SLC44A4	3.09E-14
BTN2A1	3.45E-14
THY1	3.82E-14
DSG3	3.83E-14
ORM2	4.30E-14
MEGF8	5.87E-14
KLKB1	5.94E-14
TMX3	7.99E-14
PILRA	8.11E-14
LTF	8.21E-14
BTD	9.82E-14
SERPINC1	1.12E-13
SHBG	1.24E-13
SIGLEC7	1.35E-13
TSPAN4	1.37E-13
ERBB2	1.89E-13
CTSA	1.94E-13
INHA	3.01E-13
PRSS22	3.07E-13
CFP	3.88E-13

gene	adj.P.Val
RNASE1	3.92E-13
NPR3	4.06E-13
SCARB2	4.64E-13
FOLH1	5.44E-13
IL6ST	5.73E-13
MFAP5	6.25E-13
CPVL	1.03E-12
PTCRA	1.06E-12
NOMO3	1.17E-12
HLA-A	1.17E-12
FAM171A2	1.39E-12
C4BPB	1.96E-12
TMEFF2	2.10E-12
DDR1	2.20E-12
ITGB4	2.33E-12
FBLN2	2.46E-12
PLOD1	2.85E-12
TG	2.99E-12
ADAM22	3.13E-12
ABCB11	3.29E-12
FIBIN	3.37E-12
SLC5A1	3.64E-12
SLC26A3	4.35E-12
SLC5A3	4.68E-12
CFTR	4.95E-12
HHIP	5.04E-12
ST8SIA2	5.15E-12
OLFML3	5.94E-12
CPNE9	7.26E-12
MSR1	7.83E-12
RAMP3	8.50E-12
F2R	9.11E-12
LIF	9.75E-12
RCN3	1.09E-11
SIL1	1.10E-11
F13A1	1.65E-11
MINPP1	2.21E-11
PRG4	2.44E-11
ATRN	4.56E-11
RHCG	5.13E-11
HP	6.04E-11
MUC4	7.53E-11
RNASE2	7.83E-11
QPCT	8.85E-11
MFSD8	9.41E-11
ITGAL	1.04E-10
LIPA	1.06E-10
PTGIR	1.08E-10
APOC4	1.24E-10
IGF2R	1.31E-10
ITM2C	1.48E-10
EPCAM	1.55E-10
LAMP3	1.61E-10
SLC38A4	1.81E-10
GNS	1.86E-10
NOX1	1.91E-10
KLK3	2.05E-10
THPO	2.08E-10
DPP7	2.32E-10
SLC4A1	2.38E-10
QSOX1	2.54E-10
ENPP4	2.57E-10
TLR5	2.57E-10
FAM20C	2.85E-10
APP	3.22E-10
CTSB	3.34E-10
TCN1	3.67E-10
ENG	3.85E-10
GABBR1	4.39E-10
FREM2	4.97E-10
SLC6A1	4.97E-10
GLRA3	5.38E-10
SLC39A6	5.44E-10
DMBT1	6.31E-10
KCNH2	7.99E-10
SIRPG	8.19E-10
ST6GAL1	8.26E-10
PAPPA	8.27E-10
ITIH4	8.37E-10
STAB1	8.41E-10
SUMF2	9.70E-10
CD69	1.20E-09
CD14	1.26E-09
XDH	1.35E-09
CNNM4	1.71E-09
ST14	1.79E-09
SELL	1.92E-09
NEO1	2.30E-09
PRB2	2.30E-09
LALBA	2.91E-09
SLC39A14	2.98E-09
ECM1	3.11E-09
SCARB1	4.34E-09
ZP3	4.37E-09
KCNJ3	4.50E-09
MPZL2	4.61E-09
SERPIND1	4.63E-09
CD40LG	6.28E-09
HLA-B	6.69E-09
GCGR	6.85E-09
RXFP1	6.88E-09

gene	adj.P.Val
LBP	7.00E-09
LGALS3BP	7.96E-09
BPGM	8.04E-09
LPAR4	9.39E-09
F13B	1.23E-08
ACP5	1.24E-08
ORAI1	1.26E-08
INSR	1.36E-08
HPSE	1.36E-08
HGFAC	1.78E-08
TNFRSF11B	1.86E-08
THBS2	1.90E-08
CALCRL	1.92E-08
ENPP7	2.05E-08
PANX1	2.14E-08
SYPL1	2.55E-08
UGT2B7	2.71E-08
AP1AR	2.85E-08
TSPAN15	2.93E-08
PTPRJ	3.22E-08
SSR1	3.26E-08
FKBP9	3.66E-08
SLC29A2	3.84E-08
ALPP	4.02E-08
P2RX7	4.03E-08
LAMB1	4.50E-08
AGRN	5.32E-08
HSPG2	5.83E-08
VNN1	5.86E-08
FLRT3	6.20E-08
STT3A	6.38E-08
NPTX1	6.46E-08
FETUB	6.48E-08
EDEM3	6.65E-08
SEMA4D	6.93E-08
ABCA4	7.49E-08
CLEC2D	8.03E-08
PTK7	8.97E-08
LRG1	1.38E-07
CD6	1.46E-07
ZFR	1.57E-07
SLC5A5	1.70E-07
PDLIM5	1.98E-07
KLK1	1.99E-07
ERBB3	2.06E-07
SLC6A4	2.27E-07
LSAMP	2.42E-07
GUCY2C	2.94E-07
TMEM62	3.46E-07
PRLR	3.49E-07
HTR7	3.49E-07
GPC1	4.59E-07
LRRC32	4.82E-07
POMC	5.01E-07
SLC44A1	5.05E-07
UGT2B4	5.76E-07
BTN1A1	5.91E-07
CTLA4	5.92E-07
CHI3L1	6.03E-07
PRCP	6.73E-07
DSG2	6.79E-07
PLXDC2	6.81E-07
TNFSF13B	6.93E-07
CSF1R	6.96E-07
IL7R	7.40E-07
EPHA3	7.74E-07
SHH	7.92E-07
IGFBP5	8.07E-07
CSF3R	8.21E-07
IGSF11	9.14E-07
LNPEP	9.30E-07
DSC2	9.47E-07
TMEM30A	1.06E-06
PLA2G15	1.14E-06
SERPINE1	1.37E-06
CADM4	1.61E-06
MIA3	1.66E-06
MUC7	1.81E-06
PLXNB2	1.84E-06
HLA-DQB2	2.05E-06
ITGB2	2.06E-06
SEMA4B	3.02E-06
XCL1	3.08E-06
BMP1	3.21E-06
CEACAM8	3.63E-06
SLC9A1	3.66E-06
SLC22A5	4.03E-06
IFNG	4.14E-06
CD22	4.30E-06
CLEC2A	4.34E-06
CTSH	4.75E-06
IL1RL1	4.85E-06
ADAM10	5.24E-06
MR1	5.24E-06
G6PC2	6.15E-06
AMY2A	6.89E-06
PDGFRB	6.91E-06
LYPD3	7.21E-06
CSF2RA	7.44E-06
ACE	8.47E-06
MAG	9.10E-06

gene	adj.P.Val
PIP	9.66E-06
CPN2	1.16E-05
IGFBP4	1.17E-05
TM4SF4	1.23E-05
PCSK9	1.25E-05
GNPTG	1.45E-05
CD55	1.72E-05
FCN3	1.75E-05
CYP2W1	2.19E-05
CREG2	2.26E-05
KCNJ1	2.56E-05
FUCA2	2.60E-05
CD109	2.67E-05
LMO7	2.79E-05
TREM2	2.84E-05
ITGA3	3.03E-05
PHC3	3.36E-05
ITGA11	3.42E-05
ABCC1	3.58E-05
IDUA	3.68E-05
SERPINA5	4.35E-05
ICAM3	4.64E-05
P2RX1	4.67E-05
ZNF92	4.87E-05
STEAP3	5.62E-05
ABCG8	5.70E-05
LRRC26	6.44E-05
CSPG4	6.55E-05
TLR2	6.62E-05
CD58	6.64E-05
ERBB4	6.68E-05
SORT1	7.21E-05
KIAA0319L	7.56E-05
SLC44A2	7.85E-05
FCGBP	7.97E-05
CD46	8.53E-05
COL6A3	8.98E-05
SLC22A7	9.92E-05
DNAH10	0.0001023
ARSB	0.0001024
ELOVL4	0.000105
SLC31A1	0.000105
NPC1L1	0.0001195
GUSB	0.0001251
F2RL3	0.0001266
PTGFRN	0.0001325
C4A	0.0001466
KIR3DL1	0.0001907
FUT11	0.0002015
MAFK	0.0002205
IL1RN	0.0002245
SFTPD	0.0002336
ATG9A	0.0002461
CFHR1	0.0002555
ADAM17	0.0003809
APCS	0.000399
WNT3	0.0004553
TAS2R14	0.0004587
CD96	0.0004973
TM9SF3	0.0005342
LGR5	0.0005456
CEACAM1	0.0006992
NEFL	0.00074
MFSD2A	0.0008568
MPZL1	0.0009434
CWC27	0.0009618
LAIR1	0.0009766
SELE	0.0010199
CETP	0.001099
MAN2A1	0.0011677
PRB4	0.0011901
NCEH1	0.0012917
CD82	0.001365
VCAM1	0.0013766
LGMN	0.0017909
GFRA1	0.0018028
FCGR2A	0.0018887
SUSD1	0.0020223
CDK12	0.0020459
ARSA	0.0020591
PTPRK	0.0022898
HIVEP1	0.0024798
CSF2RB	0.0028006
NUP98	0.0028653
HPX	0.0028795
ATF6	0.003051
F5	0.0032202
PON2	0.0033019
CA14	0.0035729
M6PR	0.0035784
SERPINA3	0.0037721
APOE	0.0037822
CFHR3	0.0041097
IL2RG	0.0041938
NEFH	0.004194
SI	0.005069
IHH	0.0051783
GLA	0.005437
TSHB	0.0056146
PLXNA1	0.0061326
DNASE1L1	0.0063806

gene	adj.P.Val
LOXL2	0.007423
HLA-DRB1	0.007816
ITGA2	0.007847
NAAA	0.007875
NUP214	0.008115
ENTPD5	0.008721
SEL1L	0.009192
CD74	0.009528
GGT1	0.010843
CST7	0.010865
GPA33	0.01116
RCN1	0.012746
SUSD2	0.013931
CES3	0.014375
SLC4A7	0.014596
LDLR	0.015365
UMOD	0.01669
CRISP3	0.01765
UGT2B15	0.018802
PCDH1	0.019697
CPD	0.01977
LRPAP1	0.01977
POFUT1	0.020014
APOL1	0.020662
NPC2	0.020876
COL6A1	0.021883
CD7	0.022785
PTDSS2	0.023925
ASAH1	0.025894
NID2	0.032875
FBN1	0.034044
SERPINA6	0.034842
LCN2	0.034998
BPTF	0.035876
DNAJB14	0.038806
ATP1B3	0.039444
SLC20A2	0.040154
RHO	0.040646
REN	0.041292
C2	0.043023
PRTN3	0.043023
SORL1	0.049723
HTR2C	0.05267
KERA	0.057753
HGSNAT	0.059134
OPRK1	0.059297
C8B	0.061699
AFM	0.066797
FSHB	0.068699
TRPV6	0.073622
TTR	0.079558
AGT	0.080471
ANGPTL3	0.083604
CD1D	0.084154
OPTC	0.08439
EDIL3	0.084492
AGA	0.086655
IL23R	0.096529
IL2RB	0.098032
ICOSLG	0.101887
IL1R2	0.103465
SLC7A1	0.112208
LY96	0.117732
GRIA2	0.121093
COL4A2	0.121884
GDNF	0.127842
EMILIN1	0.136204
WFS1	0.144937

Supplemental Table 7

Supplementary Table 7: Functional annotation of the 876 DE genes encoding N-glycosylated proteins from TCGA dataset

	Term	Overlap	P-value	Adjusted P-value	Old P-value	Old Adjusted P-value	Odds Ratio	Combined Score
Gene Ontology (GO)	**extracellular matrix organization (GO:0030198)**	72/229	6.77E-42	3.46E-38	0	0	7.178321469	680.4754505
	**positive regulation of cell proliferation (GO:0008284)**	66/424	1.77E-19	1.13E-16	0	0	3.553889894	153.4600434
	**regulation of cell migration (GO:0030334)**	54/316	5.72E-18	2.24E-15	0	0	3.901508583	154.902106
	**cell-matrix adhesion (GO:0007160)**	24/90	5.12E-13	7.46E-11	0	0	6.088280061	172.3025223
	**regulation of cell proliferation (GO:0042127)**	78/740	5.25E-13	7.45E-11	0	0	2.406516105	68.04403989
	**extracellular matrix disassembly (GO:0022617)**	22/78	1.38E-12	1.76E-10	0	0	6.439526987	175.8703527
	**positive regulation of cell migration (GO:0030335)**	37/221	1.94E-12	2.41E-10	0	0	3.822393025	103.0912297
	**cell-cell adhesion via plasma-membrane adhesion molecules (GO:0098742)**	28/142	1.78E-11	1.93E-09	0	0	4.501897228	111.438742
	**homophilic cell adhesion via plasma membrane adhesion molecules (GO:0007156)**	15/64	7.95E-08	4.22E-06	0	0	5.351027397	87.47724248
	**heterotypic cell-cell adhesion (GO:0034113)**	9/25	6.17E-07	2.58E-05	0	0	8.219178082	117.5170183
	**negative regulation of cell adhesion (GO:0007162)**	14/65	6.55E-07	2.61E-05	0	0	4.917456972	70.01967743
	**regulation of focal adhesion assembly (GO:0051893)**	11/44	2.17E-06	7.54E-05	0	0	5.707762557	74.42487642
	**positive regulation of focal adhesion assembly (GO:0051894)**	7/17	3.99E-06	1.27E-04	0	0	9.401020682	116.8649546
	**negative regulation of cell motility (GO:2000146)**	16/97	4.55E-06	1.43E-04	0	0	3.765946429	46.32102334
	**angiogenesis involved in wound healing (GO:0060055)**	5/8	7.99E-06	2.38E-04	0	0	14.26940639	167.4810028
	**positive regulation of cell-matrix adhesion (GO:0001954)**	9/33	8.50E-06	2.49E-04	0	0	6.226650062	72.70190731
	**positive regulation of adherens junction organization (GO:1903393)**	7/19	9.58E-06	2.75E-04	0	0	8.411439558	97.20360476
	**positive regulation of epithelial cell migration (GO:0010634)**	12/74	8.30E-05	0.001586046	0	0	3.702332469	34.79024848
	**regulation of extracellular matrix organization (GO:1903053)**	4/7	1.15E-04	0.002017599	0	0	13.04631442	118.331476
WikiPathways (WP)	Focal Adhesion-PI3K-Akt-mTOR-signaling pathway WP3932	60/303	3.29E-23	3.89E-21	0	0	4.521000045	234.0412101
	Focal Adhesion WP306	31/198	6.79E-10	2.46E-08	0	0	3.574558369	75.46255924
	Integrin-mediated Cell Adhesion WP185	14/101	1.28E-04	0.001311146	0	0	3.164700032	28.37214194
	TGF-B Signaling in Thyroid Cells for Epithelial-Mesenchymal Transition WP3859	5/18	8.47E-04	0.007406884	0	0	6.341958397	44.85883056
	Matrix Metalloproteinases WP129	5/30	0.009128085	0.051909109	0	0	3.805175038	17.87062168
	Amplification and Expansion of Oncogenic Pathways as Metastatic Traits WP3678	3/17	0.03598745	0.164913364	0	0	4.029008864	13.39478247

Supplemental Table 8

Supplementary Table 8: Resulting 308 DE genes encoding N-glycosylated proteins in GSE26338 dataset

This list displays the overlap between differentially-expressed genes in the GSE26338 dataset and known N-glycosylated proteins from dbPTM.

Gene.symbol	adj.P.Val
C7	0.00021473
COL17A1	0.00032831
OXTR	0.00045779
RELN	0.00062775
FAM3D	0.000799
IGFBP6	0.00084894
PIGR	0.00109503
FCER1A	0.00161609
SLC6A1	0.00271445
FCGR1A	0.00312369
JAM2	0.00351703
NEFH	0.0039843
FCN3	0.00517982
CD1B	0.00566241
CYBB	0.00738655
IL4I1	0.00785827
MME	0.00795772
DLK1	0.00824349
ADAMTS5	0.00834761
CLU	0.00837689
LTF	0.00901516
JCHAIN	0.00952398
KIT	0.00999397
APOD	0.01122914
SMPD1	0.01241789
CHST11	0.01405047
CD1A	0.01581637
NTRK1	0.01593238
TNFSF13B	0.01613476
FN1	0.0170812
ACP5	0.01738137
SERPINA5	0.01956864
SFRP1	0.02115689
MYC	0.02410919
LY96	0.02637397
HCFC1	0.02646028
PTPRC	0.02707138
TCN1	0.02714003
VCAM1	0.02987589
PIGR	0.03053991
AOAH	0.03139933
GLA	0.03151257
HBB	0.03467566
LAMP3	0.03555362
PTPRC	0.0365266
CTSF	0.03657617
ADAMDEC1	0.03662282
PLXNB2	0.03662282
EFNB2	0.03729664
IL2RA	0.03855741
P4HA1	0.03872473
CLEC2D	0.03908168
IL18BP	0.03964618
CD33	0.03964618
GZMA	0.04111542
EPCAM	0.04139243
CBLN1	0.0415397
PLXND1	0.0415397
MAL2	0.04179288
CLEC3B	0.04194968
CPVL	0.0421194
ACKR1	0.04298696
C2	0.04352683
EPHA2	0.04588013
HLA-DMB	0.04787539
CD33	0.04817963
GAL3ST1	0.05085268
YTHDF3	0.05136267
LTBP1	0.0518538
CERS2	0.05240546
CD163	0.05354541
PLOD2	0.05446937
IGF2	0.05456526
F3	0.05458351
EMC10	0.05827802
C1S	0.05980578
OLFML3	0.06134661
GRN	0.06148578
TSPAN9	0.06218356
CP	0.06385236
KCNE1	0.06486243
NPTX1	0.0651879
MMP1	0.06618106
EDEM3	0.06711955
SEL1L	0.06749721
B3GNT2	0.06790647
SIGLEC7	0.06828863
FSTL3	0.06828863
CD59	0.06852069
CD74	0.06951933
CD300LF	0.0700461
HLA-C	0.07030561
DCN	0.07037481
ATP1B1	0.07063892
HLA-DRB5	0.07118308
CSF2RA	0.07146285
HLA-G	0.07181205
TMEM26	0.07203801
HLA-DRB1	0.07203801
CTSB	0.072515
HLA-DRB3	0.07290741

Gene.symbol	adj.P.Val
NUP214	0.07291294
IGSF11	0.07379834
LGR4	0.07391314
IKBIP	0.07482864
CD28	0.07574103
LALBA	0.07580181
OLFM1	0.0764059
NPC2	0.0765377
PTPRC	0.07667442
NPR3	0.0772577
HTR2C	0.07744703
HTR7	0.08028724
ENPP4	0.08037265
GGH	0.08075994
TNFRSF21	0.08105535
CD1B	0.08143671
SLC2A1	0.08205011
ITGA4	0.08220142
OPTC	0.08267688
CTBS	0.08523224
PROS1	0.08581285
ITGAL	0.08581285
CD3G	0.08602945
MAN2B1	0.08749123
SELE	0.08776969
HLA-DPA1	0.08863785
SULF2	0.08900559
CD48	0.08925463
DMBT1	0.08933714
GCGR	0.09087724
CADM4	0.09107363
TIMP1	0.09162626
KLK6	0.0946198
ITGB2	0.09517767
ADAMTSL4	0.0952361
CD80	0.09524821
SULF1	0.09524821
HP	0.09528021
CD83	0.09617571
CD244	0.0970441
DSG2	0.09764836
SAA4	0.09818756
HGSNAT	0.09842361
IL7R	0.09857093
DSC2	0.09882618
HLA-DRA	0.10051936
PPT1	0.10097342
F10	0.10160893
IGFBP4	0.10209807
SIAE	0.10262728
PLXNC1	0.10399168
LGALS3BP	0.10576008
HLA-A	0.1060094
CNTN1	0.10616418
HEG1	0.10616418
LRP1	0.10616418
COL6A1	0.1084542
ATP1B1	0.10813222
HIVEP1	0.10849819
SIT1	0.10877759
TG	0.10909493
SSR1	0.10909493
ACAN	0.10965237
DPP4	0.10993933
HSP90B1	0.11133507
ENPP7	0.11191161
USP9X	0.11193591
LRPAP1	0.11306918
FAM209B	0.11306918
BPIFA2	0.11316527
CTSA	0.11354125
POGLUT1	0.11354125
LBP	0.11370696
C3	0.11419148
CREB3L3	0.11484129
ICOSLG	0.11482638
TRPV6	0.11491244
PTK7	0.1151565
FIP1L1	0.11598467
CHST7	0.116163
LRIG1	0.11715651
SIRPA	0.11796554
B2M	0.1202161
STT3B	0.12056288
PTGIR	0.120585
ITGAX	0.12195725
STIM1	0.12177285
SLC14A2	0.12254411
ANPEP	0.1246027
CD14	0.12623751
CES1	0.12661855
BPTF	0.1266272
FCGR2A	0.12664867
MFSD2A	0.12695713
ST8SIA4	0.12861265
SLC4A7	0.12964471
KCNH6	0.13034256
KITLG	0.13101943
CSF1R	0.13128311
UMOD	0.13469094
PLXDC2	0.13560113

Gene.symbol	adj.P.Val
OGN	0.13599787
MFAP4	0.13768498
EMILIN2	0.13774519
FASLG	0.13774519
NCEH1	0.1408656
NPR3	0.14127643
IL1RN	0.14120773
NAGA	0.14149459
FGL2	0.14187972
PKD2	0.14258903
CD69	0.14258903
CD276	0.14352513
LPL	0.14394055
MAN2A2	0.14429294
CFP	0.14435221
TINAGL1	0.14435326
FOLR1	0.14605548
PTPRK	0.14660686
PTPRC	0.14647697
C1QA	0.14738122
TRPM8	0.14761953
SLC20A2	0.14883849
HEXA	0.14919919
IFNA14	0.15018937
DRD5	0.15044521
SLC4A4	0.15044521
PTPRC	0.15046371
LGR5	0.15102788
PRRC2C	0.15110715
CD52	0.15173987
CLN5	0.15184923
PAMR1	0.15226899
AZGP1	0.15251309
ADGRE5	0.15400052
AGA	0.15415969
LIFR	0.15518347
GNPTG	0.15650897
PRB4	0.1567411
CP	0.15785171
TEK	0.15941683
GC	0.16005128
IMPAD1	0.16036034
CD69	0.16070966
FCGR3A	0.16086515
PLXNB1	0.16117859
MAFK	0.16200912
VNN2	0.16266619
CD69	0.16374546
ITGA7	0.16536035
FSTL1	0.16546558
PTPRK	0.16584515
LAMP1	0.16612852
APOM	0.16613051
LAMB2	0.16757858
HSPG2	0.16953671
SAA4	0.17181065
CALU	0.17313706
SPP1	0.1736391
LAMA2	0.17429482
ATF6	0.17490383
TNFRSF14	0.1751816
CDH6	0.17654757
IGFBP7	0.17656222
JAM3	0.17778858
MST1R	0.17799033
POFUT1	0.17824051
PRNP	0.17848802
SIRPG	0.18035182
SPP1	0.18037749
CLEC12A	0.18140224
LAIR1	0.18184225
TXNDC15	0.18227661
PNLIPRP2	0.18269568
G6PC2	0.18282461
AGT	0.18282461
SEMA4B	0.18307833
ENPEP	0.18324374
SPP1	0.18546298
CD69	0.18565236
P2RX7	0.1860947
F7	0.18674083
ICAM3	0.18731227
RHO	0.1872057
COLGALT1	0.18768322
FKBP9	0.18860475
S100PBP	0.18922597
SPP1	0.18948869
CD69	0.18988816
SGCE	0.19011233
SPP1	0.19023851
TNFRSF11B	0.19023851
SPP1	0.19053247
WNT3	0.19058873
ERMP1	0.19069836
ARSD	0.19069836
ICAM1	0.19096054
FLRT2	0.1911438
GUSB	0.19307662
HLA-B	0.19384656
CD69	0.19422823
AOC3	0.19504846
HLA-DRB5	0.19520794
F12	0.19560954
HLA-A	0.19560954

Supplemental Table 9: Accurate masses from 21T MALDI-FTICR DDA data

Name	21T experimental mass	Theoretical mass	ppm error
Hex3HxNAc2	933.31688	933.317001	-0.13
Hex5HexNAc2	1257.42247	1257.422647	-0.14
Hex3dHex1HexNAc3	1282.45432	1282.454283	0.03
Hex6HexNAc2	1419.47530	1419.475470	-0.12
Hex4dHex1HexNAc3	1444.50788	1444.507106	0.54
Hex3dHex1HexNAc4	1485.53418	1485.533656	0.35
Hex4HexNAc4	1501.52837	1501.528570	-0.13
Hex7HexNAc2	1581.52821	1581.528293	-0.05
Hex4dHex1HexNAc4	1647.58700	1647.586479	0.32
Hex5HexNAc4	1663.58113	1663.581393	-0.16
Hex5dHex1HexNAc4	1809.63931	1809.639302	0.00
Hex4dHex1HexNAc5	1850.66465	1850.665852	-0.65

Supplemental Table 10: Glycoworkbench table for m/z 933.31688 (21T MALDI-FTICR DDA data). The N-glycan circled in red was selected as the most relevant glycoform detected based on additional literature review and shown as annotated spectra in Supplemental Figure 6.

Structure	Coverage	RMSD	RMSD PPM	Assigned	>10% assigned	>5% assigned
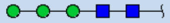	64.0979	0.0389	54.5255	7/11 (63.64%)	4/7 (57.14%)	7/11 (63.64%)
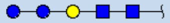	64.0979	0.0389	54.5255	7/11 (63.64%)	4/7 (57.14%)	7/11 (63.64%)
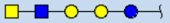	64.0979	0.0389	54.5255	7/11 (63.64%)	4/7 (57.14%)	7/11 (63.64%)
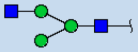	64.0979	0.0389	54.5255	7/11 (63.64%)	4/7 (57.14%)	7/11 (63.64%)
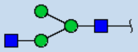	64.0979	0.0389	54.5255	7/11 (63.64%)	4/7 (57.14%)	7/11 (63.64%)
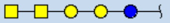	64.0979	0.0389	54.5255	7/11 (63.64%)	4/7 (57.14%)	7/11 (63.64%)
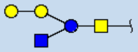	64.0979	0.0389	54.5255	7/11 (63.64%)	4/7 (57.14%)	7/11 (63.64%)
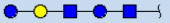	61.7049	0.0416	57.7674	6/11 (54.55%)	4/7 (57.14%)	6/11 (54.55%)
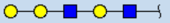	61.7049	0.0416	57.7674	6/11 (54.55%)	4/7 (57.14%)	6/11 (54.55%)
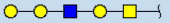	61.7049	0.0416	57.7674	6/11 (54.55%)	4/7 (57.14%)	6/11 (54.55%)
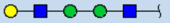	61.7049	0.0416	57.7674	6/11 (54.55%)	4/7 (57.14%)	6/11 (54.55%)
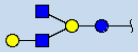	61.7049	0.0416	57.7674	6/11 (54.55%)	4/7 (57.14%)	6/11 (54.55%)
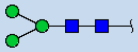	61.2430	0.0419	58.3262	6/11 (54.55%)	4/7 (57.14%)	6/11 (54.55%)
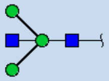	61.2430	0.0419	58.3262	6/11 (54.55%)	4/7 (57.14%)	6/11 (54.55%)
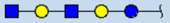	61.7049	0.0416	57.7674	6/11 (54.55%)	4/7 (57.14%)	6/11 (54.55%)
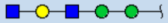	61.7049	0.0416	57.7674	6/11 (54.55%)	4/7 (57.14%)	6/11 (54.55%)
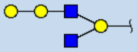	61.7049	0.0416	57.7674	6/11 (54.55%)	4/7 (57.14%)	6/11 (54.55%)
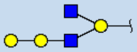	61.7049	0.0416	57.7674	6/11 (54.55%)	4/7 (57.14%)	6/11 (54.55%)
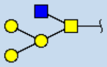	61.2430	0.0419	58.3262	6/11 (54.55%)	4/7 (57.14%)	6/11 (54.55%)
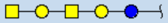	61.7049	0.0416	57.7674	6/11 (54.55%)	4/7 (57.14%)	6/11 (54.55%)
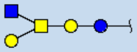	61.7049	0.0416	57.7674	6/11 (54.55%)	4/7 (57.14%)	6/11 (54.55%)
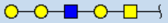	61.7049	0.0416	57.7674	6/11 (54.55%)	4/7 (57.14%)	6/11 (54.55%)
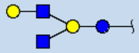	61.7049	0.0416	57.7674	6/11 (54.55%)	4/7 (57.14%)	6/11 (54.55%)
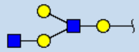	61.7049	0.0416	57.7674	6/11 (54.55%)	4/7 (57.14%)	6/11 (54.55%)
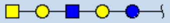	61.7049	0.0416	57.7674	6/11 (54.55%)	4/7 (57.14%)	6/11 (54.55%)

Supplemental Table 11: Glycoworkbench table for m/z 1257.42247 (21T MALDI-FTICR DDA data). The N-glycan circled in red was selected as the most relevant glycoform detected based on additional literature review and shown as annotated spectra in Supplemental Figure 7.

Structure	Coverage	RMSD	RMSD PPM	▽ Assigned	>10% assigned	>5% assigned
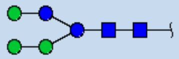	49.1446	0.1358	136.2377	5/12 (41.67%)	3/7 (42.86%)	5/12 (41.67%)
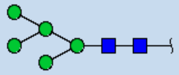	49.1446	0.1358	136.2377	5/12 (41.67%)	3/7 (42.86%)	5/12 (41.67%)
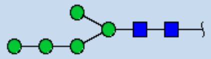	49.1446	0.1358	136.2377	5/12 (41.67%)	3/7 (42.86%)	5/12 (41.67%)
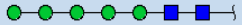	49.1446	0.1358	136.2377	5/12 (41.67%)	3/7 (42.86%)	5/12 (41.67%)
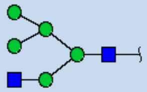	49.1446	0.1358	136.2377	5/12 (41.67%)	3/7 (42.86%)	5/12 (41.67%)
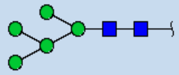	49.1446	0.1358	136.2377	5/12 (41.67%)	3/7 (42.86%)	5/12 (41.67%)
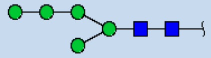	49.1446	0.1358	136.2377	5/12 (41.67%)	3/7 (42.86%)	5/12 (41.67%)
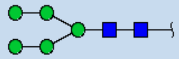	49.1446	0.1358	136.2377	5/12 (41.67%)	3/7 (42.86%)	5/12 (41.67%)
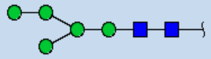	49.1446	0.1358	136.2377	5/12 (41.67%)	3/7 (42.86%)	5/12 (41.67%)
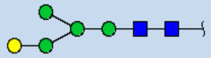	49.1446	0.1358	136.2377	5/12 (41.67%)	3/7 (42.86%)	5/12 (41.67%)
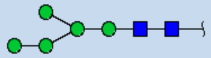	49.1446	0.1358	136.2377	5/12 (41.67%)	3/7 (42.86%)	5/12 (41.67%)
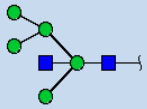	49.1446	0.1358	136.2377	5/12 (41.67%)	3/7 (42.86%)	5/12 (41.67%)
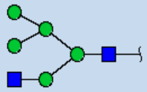	49.1446	0.1358	136.2377	5/12 (41.67%)	3/7 (42.86%)	5/12 (41.67%)
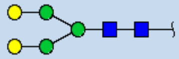	49.1446	0.1358	136.2377	5/12 (41.67%)	3/7 (42.86%)	5/12 (41.67%)
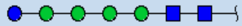	49.1446	0.1358	136.2377	5/12 (41.67%)	3/7 (42.86%)	5/12 (41.67%)
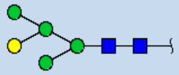	49.1446	0.1358	136.2377	5/12 (41.67%)	3/7 (42.86%)	5/12 (41.67%)
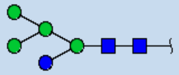	49.1446	0.1358	136.2377	5/12 (41.67%)	3/7 (42.86%)	5/12 (41.67%)

Supplemental Table 12: Glycoworkbench table for m/z 1282.45432 (21T MALDI-FTICR DDA data). The N-glycan circled in red was selected as the most relevant glycoform detected based on additional literature review and shown as annotated spectra in Supplemental Figure 8.

Structure	Coverage	RMSD	RMSD PPM	Assigned	>10% assigned	>5% assigned
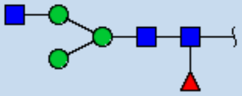	65.3292	0.0766	85.5373	5/7 (71.43%)	5/7 (71.43%)	5/7 (71.43%)
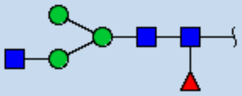	65.3292	0.0766	85.5373	5/7 (71.43%)	5/7 (71.43%)	5/7 (71.43%)
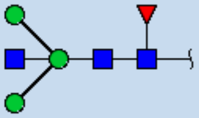	65.3292	0.0766	85.5373	5/7 (71.43%)	5/7 (71.43%)	5/7 (71.43%)
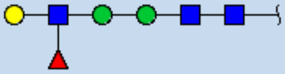	65.3292	0.0766	85.5373	5/7 (71.43%)	5/7 (71.43%)	5/7 (71.43%)
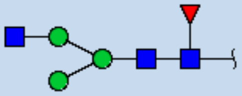	65.3292	0.0766	85.5373	5/7 (71.43%)	5/7 (71.43%)	5/7 (71.43%)
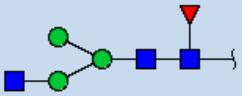	65.3292	0.0766	85.5373	5/7 (71.43%)	5/7 (71.43%)	5/7 (71.43%)
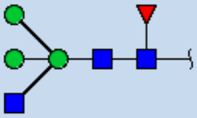	65.3292	0.0766	85.5373	5/7 (71.43%)	5/7 (71.43%)	5/7 (71.43%)

Supplemental Table 13: Glycoworkbench table for m/z 1419.47530 (21T MALDI-FTICR DDA data). The N-glycan circled in red was selected as the most relevant glycoform detected based on additional literature review and shown as annotated spectra in Supplemental Figure 9.

Structure	Coverage	RMSD	RMSD PPM	Assigned	>10% assigned	>5% assigned
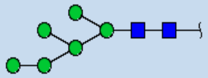	67.6042	0.1709	155.8453	2/4 (50.00%)	2/4 (50.00%)	2/4 (50.00%)
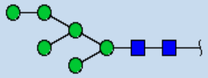	67.6042	0.1709	155.8453	2/4 (50.00%)	2/4 (50.00%)	2/4 (50.00%)
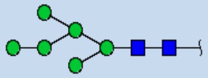	67.6042	0.1709	155.8453	2/4 (50.00%)	2/4 (50.00%)	2/4 (50.00%)
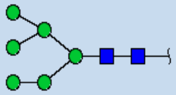	67.6042	0.1709	155.8453	2/4 (50.00%)	2/4 (50.00%)	2/4 (50.00%)
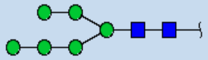	67.6042	0.1709	155.8453	2/4 (50.00%)	2/4 (50.00%)	2/4 (50.00%)
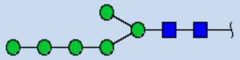	67.6042	0.1709	155.8453	2/4 (50.00%)	2/4 (50.00%)	2/4 (50.00%)
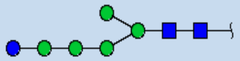	67.6042	0.1709	155.8453	2/4 (50.00%)	2/4 (50.00%)	2/4 (50.00%)
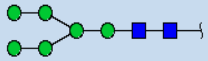	67.6042	0.1709	155.8453	2/4 (50.00%)	2/4 (50.00%)	2/4 (50.00%)
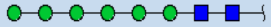	67.6042	0.1709	155.8453	2/4 (50.00%)	2/4 (50.00%)	2/4 (50.00%)
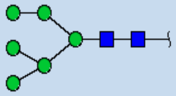	67.6042	0.1709	155.8453	2/4 (50.00%)	2/4 (50.00%)	2/4 (50.00%)
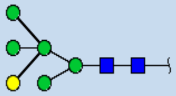	67.6042	0.1709	155.8453	2/4 (50.00%)	2/4 (50.00%)	2/4 (50.00%)
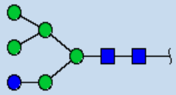	67.6042	0.1709	155.8453	2/4 (50.00%)	2/4 (50.00%)	2/4 (50.00%)
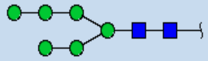	67.6042	0.1709	155.8453	2/4 (50.00%)	2/4 (50.00%)	2/4 (50.00%)
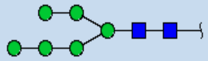	67.6042	0.1709	155.8453	2/4 (50.00%)	2/4 (50.00%)	2/4 (50.00%)
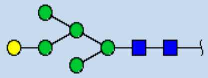	67.6042	0.1709	155.8453	2/4 (50.00%)	2/4 (50.00%)	2/4 (50.00%)
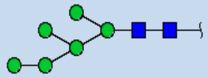	67.6042	0.1709	155.8453	2/4 (50.00%)	2/4 (50.00%)	2/4 (50.00%)

Supplemental Table 14: Glycoworkbench table for m/z 1444.50788 (21T MALDI-FTICR DDA data). The N-glycan circled in red was selected as the most relevant glycoform detected based on additional literature review and shown as annotated spectra in Supplemental Figure 10.

Structure	Coverage	RMSD	RMSD PPM	Assigned	>10% assigned	>5% assigned
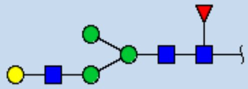	73.7158	0.4409	408.9515	5/7 (71.43%)	5/7 (71.43%)	5/7 (71.43%)
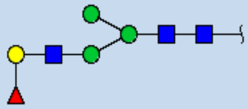	73.7158	0.4409	408.9515	5/7 (71.43%)	5/7 (71.43%)	5/7 (71.43%)
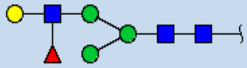	73.7158	0.4409	408.9515	5/7 (71.43%)	5/7 (71.43%)	5/7 (71.43%)
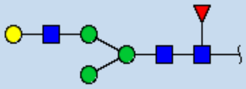	73.7158	0.4409	408.9515	5/7 (71.43%)	5/7 (71.43%)	5/7 (71.43%)
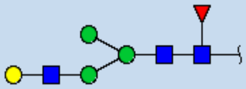	73.7158	0.4409	408.9515	5/7 (71.43%)	5/7 (71.43%)	5/7 (71.43%)
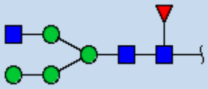	73.7158	0.4409	408.9515	5/7 (71.43%)	5/7 (71.43%)	5/7 (71.43%)
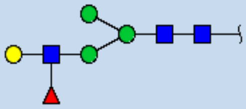	73.7158	0.4409	408.9515	5/7 (71.43%)	5/7 (71.43%)	5/7 (71.43%)
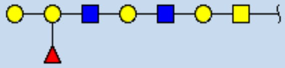	73.7158	0.4409	408.9515	5/7 (71.43%)	5/7 (71.43%)	5/7 (71.43%)
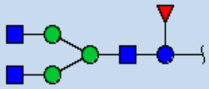	73.7158	0.4409	408.9515	5/7 (71.43%)	5/7 (71.43%)	5/7 (71.43%)
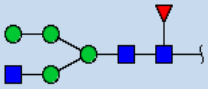	73.7158	0.4409	408.9515	5/7 (71.43%)	5/7 (71.43%)	5/7 (71.43%)
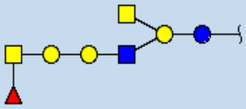	73.7158	0.4409	408.9515	5/7 (71.43%)	5/7 (71.43%)	5/7 (71.43%)
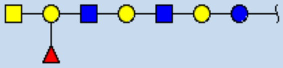	73.7158	0.4409	408.9515	5/7 (71.43%)	5/7 (71.43%)	5/7 (71.43%)
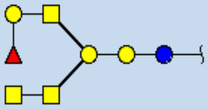	73.7158	0.4409	408.9515	5/7 (71.43%)	5/7 (71.43%)	5/7 (71.43%)

Supplemental Table 15: Glycoworkbench table for m/z 1485.53418 (21T MALDI-FTICR DDA data). The N-glycan circled in red was selected as the most relevant glycoform detected based on additional literature review and shown as annotated spectra in Supplemental Figure 11.

Structure	Coverage	RMSD	RMSD PPM	▿ Assigned	>10% assigned	>5% assigned
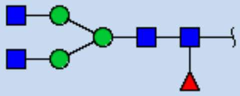	65.6390	0.0175	13.6311	5/8 (62.50%)	5/8 (62.50%)	5/8 (62.50%)
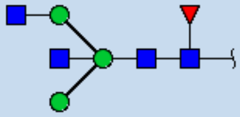	65.6390	0.0175	13.6311	5/8 (62.50%)	5/8 (62.50%)	5/8 (62.50%)
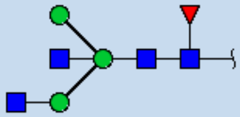	65.6390	0.0175	13.6311	5/8 (62.50%)	5/8 (62.50%)	5/8 (62.50%)
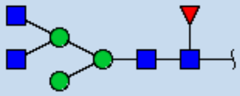	65.6390	0.0175	13.6311	5/8 (62.50%)	5/8 (62.50%)	5/8 (62.50%)
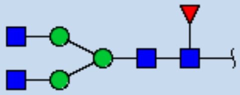	65.6390	0.0175	13.6311	5/8 (62.50%)	5/8 (62.50%)	5/8 (62.50%)
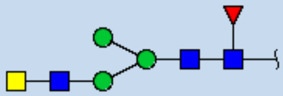	65.6390	0.0175	13.6311	5/8 (62.50%)	5/8 (62.50%)	5/8 (62.50%)
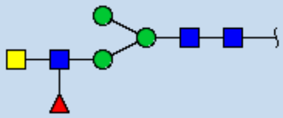	65.6390	0.0175	13.6311	5/8 (62.50%)	5/8 (62.50%)	5/8 (62.50%)
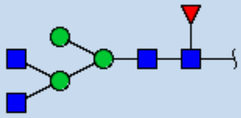	65.6390	0.0175	13.6311	5/8 (62.50%)	5/8 (62.50%)	5/8 (62.50%)

Supplemental Table 16: Glycoworkbench table for m/z 1501.52837 (21T MALDI-FTICR DDA data). The N-glycan circled in red was selected as the most relevant glycoform detected based on additional literature review and shown as annotated spectra in Supplemental Figure 12.

Structure	Coverage	RMSD	RMSD PPM	Assigned	>10% assigned	>5% assigned
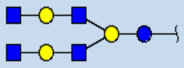	50.2272	0.4171	314.0458	6/15 (40.00%)	6/15 (40.00%)	6/15 (40.00%)
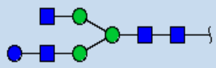	50.2272	0.4171	314.0458	6/15 (40.00%)	6/15 (40.00%)	6/15 (40.00%)
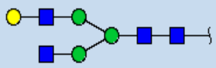	50.2272	0.4171	314.0458	6/15 (40.00%)	6/15 (40.00%)	6/15 (40.00%)
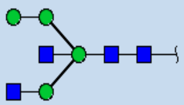	50.2272	0.4171	314.0458	6/15 (40.00%)	6/15 (40.00%)	6/15 (40.00%)
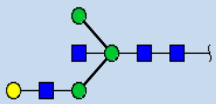	50.2272	0.4171	314.0458	6/15 (40.00%)	6/15 (40.00%)	6/15 (40.00%)
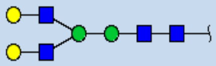	50.2272	0.4171	314.0458	6/15 (40.00%)	6/15 (40.00%)	6/15 (40.00%)
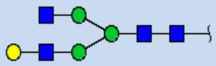	50.2272	0.4171	314.0458	6/15 (40.00%)	6/15 (40.00%)	6/15 (40.00%)
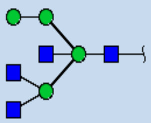	50.2272	0.4171	314.0458	6/15 (40.00%)	6/15 (40.00%)	6/15 (40.00%)
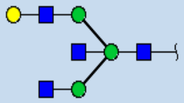	50.2272	0.4171	314.0458	6/15 (40.00%)	6/15 (40.00%)	6/15 (40.00%)
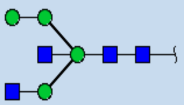	50.2272	0.4171	314.0458	6/15 (40.00%)	6/15 (40.00%)	6/15 (40.00%)
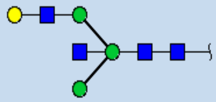	50.2272	0.4171	314.0458	6/15 (40.00%)	6/15 (40.00%)	6/15 (40.00%)
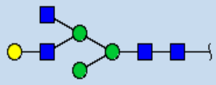	50.2272	0.4171	314.0458	6/15 (40.00%)	6/15 (40.00%)	6/15 (40.00%)
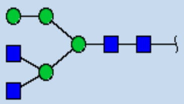	50.2272	0.4171	314.0458	6/15 (40.00%)	6/15 (40.00%)	6/15 (40.00%)
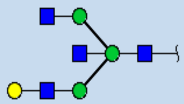	50.2272	0.4171	314.0458	6/15 (40.00%)	6/15 (40.00%)	6/15 (40.00%)

Supplemental Table 17: Glycoworkbench table for m/z 1581.52821 (21T MALDI-FTICR DDA data). The N-glycan circled in red was selected as the most relevant glycoform detected based on additional literature review and shown as annotated spectra in Supplemental Figure 13.

Structure	Coverage	RMSD	RMSD PPM	Assigned	>10% assigned	>5% assigned
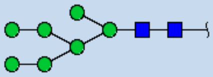	66.7536	0.1553	128.4401	2/4 (50.00%)	2/4 (50.00%)	2/4 (50.00%)
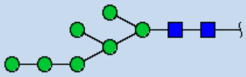	66.7536	0.1553	128.4401	2/4 (50.00%)	2/4 (50.00%)	2/4 (50.00%)
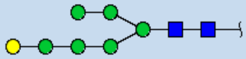	66.7536	0.1553	128.4401	2/4 (50.00%)	2/4 (50.00%)	2/4 (50.00%)
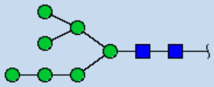	66.7536	0.1553	128.4401	2/4 (50.00%)	2/4 (50.00%)	2/4 (50.00%)
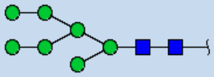	66.7536	0.1553	128.4401	2/4 (50.00%)	2/4 (50.00%)	2/4 (50.00%)
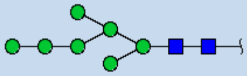	66.7536	0.1553	128.4401	2/4 (50.00%)	2/4 (50.00%)	2/4 (50.00%)
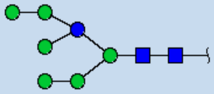	66.7536	0.1553	128.4401	2/4 (50.00%)	2/4 (50.00%)	2/4 (50.00%)
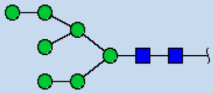	66.7536	0.1553	128.4401	2/4 (50.00%)	2/4 (50.00%)	2/4 (50.00%)
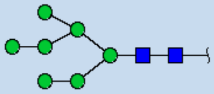	66.7536	0.1553	128.4401	2/4 (50.00%)	2/4 (50.00%)	2/4 (50.00%)
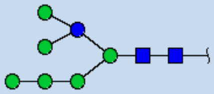	66.7536	0.1553	128.4401	2/4 (50.00%)	2/4 (50.00%)	2/4 (50.00%)
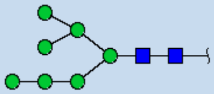	66.7536	0.1553	128.4401	2/4 (50.00%)	2/4 (50.00%)	2/4 (50.00%)
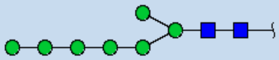	66.7536	0.1553	128.4401	2/4 (50.00%)	2/4 (50.00%)	2/4 (50.00%)
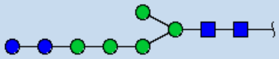	66.7536	0.1553	128.4401	2/4 (50.00%)	2/4 (50.00%)	2/4 (50.00%)
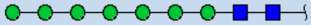	66.7536	0.1553	128.4401	2/4 (50.00%)	2/4 (50.00%)	2/4 (50.00%)
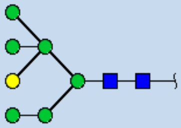	66.7536	0.1553	128.4401	2/4 (50.00%)	2/4 (50.00%)	2/4 (50.00%)

Structure	Coverage	RMSD	RMSD PPM	Assigned	>10% assigned	>5% assigned
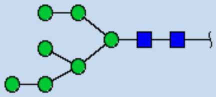	66.7536	0.1553	128.4401	2/4 (50.00%)	2/4 (50.00%)	2/4 (50.00%)
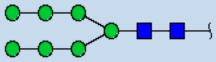	66.7536	0.1553	128.4401	2/4 (50.00%)	2/4 (50.00%)	2/4 (50.00%)
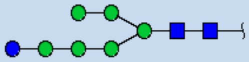	66.7536	0.1553	128.4401	2/4 (50.00%)	2/4 (50.00%)	2/4 (50.00%)
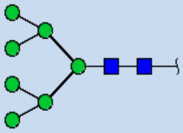	66.7536	0.1553	128.4401	2/4 (50.00%)	2/4 (50.00%)	2/4 (50.00%)
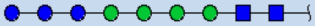	66.7536	0.1553	128.4401	2/4 (50.00%)	2/4 (50.00%)	2/4 (50.00%)
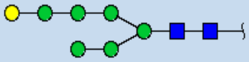	66.7536	0.1553	128.4401	2/4 (50.00%)	2/4 (50.00%)	2/4 (50.00%)
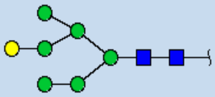	66.7536	0.1553	128.4401	2/4 (50.00%)	2/4 (50.00%)	2/4 (50.00%)
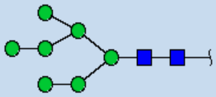	66.7536	0.1553	128.4401	2/4 (50.00%)	2/4 (50.00%)	2/4 (50.00%)
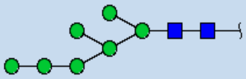	66.7536	0.1553	128.4401	2/4 (50.00%)	2/4 (50.00%)	2/4 (50.00%)
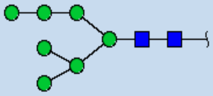	66.7536	0.1553	128.4401	2/4 (50.00%)	2/4 (50.00%)	2/4 (50.00%)
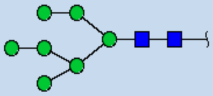	66.7536	0.1553	128.4401	2/4 (50.00%)	2/4 (50.00%)	2/4 (50.00%)
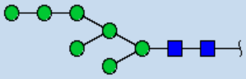	66.7536	0.1553	128.4401	2/4 (50.00%)	2/4 (50.00%)	2/4 (50.00%)

Supplemental Table 18: Glycoworkbench table for m/z 1647.58700 (21T MALDI-FTICR DDA data). The N-glycan circled in red was selected as the most relevant glycoform detected based on additional literature review and shown as annotated spectra in Supplemental Figure 14.

Structure	Coverage	RMSD	RMSD PPM	Assigned	>10% assigned	>5% assigned
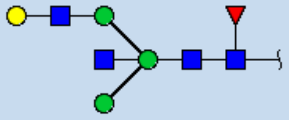	70.2555	0.4387	342.2293	5/7 (71.43%)	5/7 (71.43%)	5/7 (71.43%)
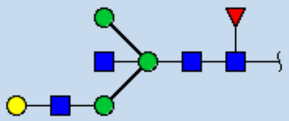	70.2555	0.4387	342.2293	5/7 (71.43%)	5/7 (71.43%)	5/7 (71.43%)
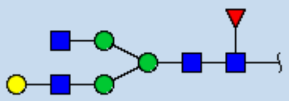	70.2555	0.4387	342.2293	5/7 (71.43%)	5/7 (71.43%)	5/7 (71.43%)
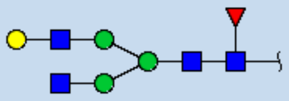	70.2555	0.4387	342.2293	5/7 (71.43%)	5/7 (71.43%)	5/7 (71.43%)
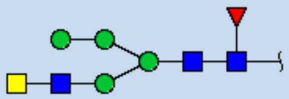	70.2555	0.4387	342.2293	5/7 (71.43%)	5/7 (71.43%)	5/7 (71.43%)
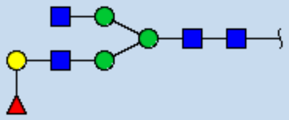	70.2555	0.4387	342.2293	5/7 (71.43%)	5/7 (71.43%)	5/7 (71.43%)
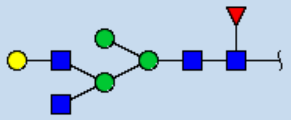	70.2555	0.4387	342.2293	5/7 (71.43%)	5/7 (71.43%)	5/7 (71.43%)
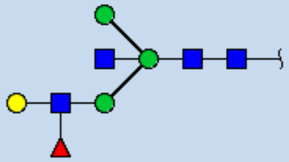	70.2555	0.4387	342.2293	5/7 (71.43%)	5/7 (71.43%)	5/7 (71.43%)

Supplemental Table 19: Glycoworkbench table for m/z 1663.58113 (21T MALDI-FTICR DDA data). The N-glycan circled in red was selected as the most relevant glycoform detected based on additional literature review and shown as annotated spectra in Supplemental Figure 15.

Structure	Coverage	RMSD	RMSD PPM	Assigned	>10% assigned	>5% assigned
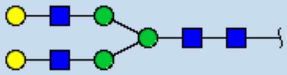	72.7077	0.1107	86.1643	11/14 (78.57%)	8/10 (80.00%)	10/13 (76.92%)
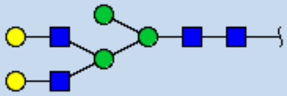	72.7077	0.1107	86.1643	11/14 (78.57%)	8/10 (80.00%)	10/13 (76.92%)
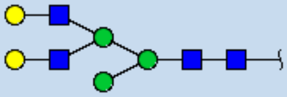	72.7077	0.1107	86.1643	11/14 (78.57%)	8/10 (80.00%)	10/13 (76.92%)
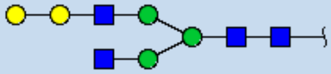	72.7077	0.1107	86.1643	11/14 (78.57%)	8/10 (80.00%)	10/13 (76.92%)
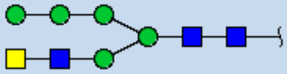	72.7077	0.1107	86.1643	11/14 (78.57%)	8/10 (80.00%)	10/13 (76.92%)
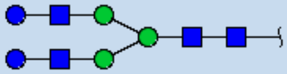	72.7077	0.1107	86.1643	11/14 (78.57%)	8/10 (80.00%)	10/13 (76.92%)
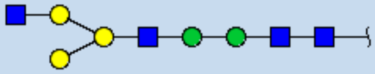	72.7077	0.1107	86.1643	11/14 (78.57%)	8/10 (80.00%)	10/13 (76.92%)

Supplemental Table 20: Glycoworkbench table for m/z 1809.63931 (21T MALDI-FTICR DDA data). The N-glycan circled in red was selected as the most relevant glycoform detected based on additional literature review and shown as annotated spectra in Supplemental Figure 16.

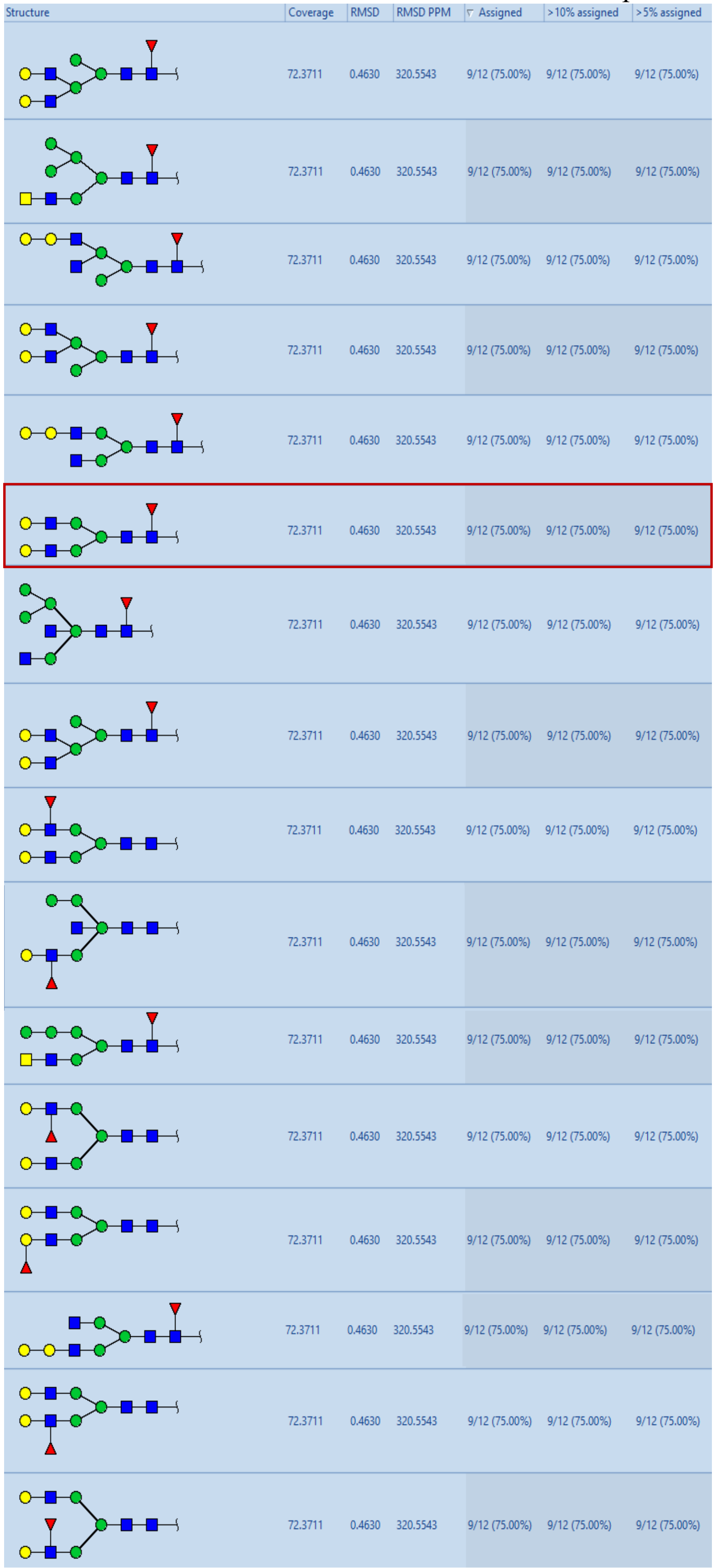

Structure	Coverage	RMSD	RMSD PPM	Assigned	>10% assigned	>5% assigned
	72.3711	0.4630	320.5543	9/12 (75.00%)	9/12 (75.00%)	9/12 (75.00%)
	72.3711	0.4630	320.5543	9/12 (75.00%)	9/12 (75.00%)	9/12 (75.00%)
	72.3711	0.4630	320.5543	9/12 (75.00%)	9/12 (75.00%)	9/12 (75.00%)
	72.3711	0.4630	320.5543	9/12 (75.00%)	9/12 (75.00%)	9/12 (75.00%)
	72.3711	0.4630	320.5543	9/12 (75.00%)	9/12 (75.00%)	9/12 (75.00%)
	72.3711	0.4630	320.5543	9/12 (75.00%)	9/12 (75.00%)	9/12 (75.00%)
	72.3711	0.4630	320.5543	9/12 (75.00%)	9/12 (75.00%)	9/12 (75.00%)
	72.3711	0.4630	320.5543	9/12 (75.00%)	9/12 (75.00%)	9/12 (75.00%)
	72.3711	0.4630	320.5543	9/12 (75.00%)	9/12 (75.00%)	9/12 (75.00%)
	72.3711	0.4630	320.5543	9/12 (75.00%)	9/12 (75.00%)	9/12 (75.00%)
	72.3711	0.4630	320.5543	9/12 (75.00%)	9/12 (75.00%)	9/12 (75.00%)
	72.3711	0.4630	320.5543	9/12 (75.00%)	9/12 (75.00%)	9/12 (75.00%)
	72.3711	0.4630	320.5543	9/12 (75.00%)	9/12 (75.00%)	9/12 (75.00%)
	72.3711	0.4630	320.5543	9/12 (75.00%)	9/12 (75.00%)	9/12 (75.00%)
	72.3711	0.4630	320.5543	9/12 (75.00%)	9/12 (75.00%)	9/12 (75.00%)
	72.3711	0.4630	320.5543	9/12 (75.00%)	9/12 (75.00%)	9/12 (75.00%)

Supplemental Table 21: Glycoworkbench table for m/z 1850.66465 (21T MALDI-FTICR DDA data). The N-glycan circled in red was selected as the most relevant glycoform detected based on additional literature review and shown as annotated spectra in Supplemental Figure 17.

Structure	Coverage	RMSD	RMSD PPM	Assigned	>10% assigned	>5% assigned
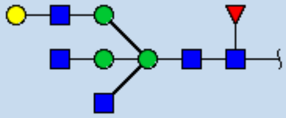	39.3507	0.4594	303.6927	11/30 (36.67%)	10/29 (34.48%)	11/30 (36.67%)
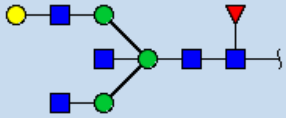	39.3507	0.4594	303.6927	11/30 (36.67%)	10/29 (34.48%)	11/30 (36.67%)
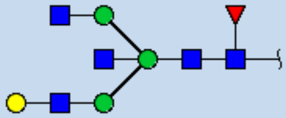	39.3507	0.4594	303.6927	11/30 (36.67%)	10/29 (34.48%)	11/30 (36.67%)
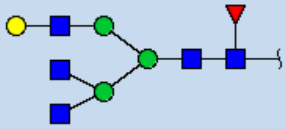	40.8122	0.5290	470.1836	11/30 (36.67%)	11/29 (37.93%)	11/30 (36.67%)
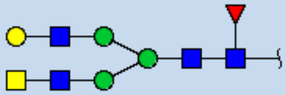	40.8122	0.5290	470.1836	11/30 (36.67%)	11/29 (37.93%)	11/30 (36.67%)
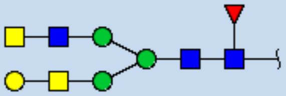	40.8122	0.5290	470.1836	11/30 (36.67%)	11/29 (37.93%)	11/30 (36.67%)
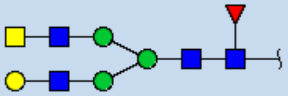	40.8122	0.5290	470.1836	11/30 (36.67%)	11/29 (37.93%)	11/30 (36.67%)
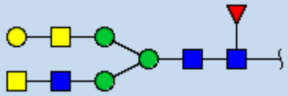	40.8122	0.5290	470.1836	11/30 (36.67%)	11/29 (37.93%)	11/30 (36.67%)
